# Semihydrogenation of Alkynes Catalyzed by a Pyridone Borane Complex: Frustrated Lewis Pair Reactivity and Boron–Ligand Cooperation in Concert

**DOI:** 10.1002/chem.202001276

**Published:** 2020-09-18

**Authors:** Felix Wech, Max Hasenbeck, Urs Gellrich

**Affiliations:** ^1^ Institut für Organische Chemie Justus-Liebig-Universität Gießen Heinrich-Buff-Ring 17 35392 Gießen Germany

**Keywords:** alkynes, boron–ligand cooperation, density functional calculations, frustrated Lewis pair, hydrogenation

## Abstract

The metal‐free *cis* selective hydrogenation of alkynes catalyzed by a boroxypyridine is reported. A variety of internal alkynes are hydrogenated at 80 °C under 5 bar H_2_ with good yields and stereoselectivity. Furthermore, the catalyst described herein enables the first metal‐free semihydrogenation of terminal alkynes. Mechanistic investigations, substantiated by DFT computations, reveal that the mode of action by which the boroxypyridine activates H_2_ is reminiscent of the reactivity of an intramolecular frustrated Lewis pair. However, it is the change in the coordination mode of the boroxypyridine upon H_2_ activation that allows the dissociation of the formed pyridone borane complex and subsequent hydroboration of an alkyne. This change in the coordination mode upon bond activation is described by the term boron‐ligand cooperation.

## Graphical abstract

**A case of important cooperation**: The semihydrogenation of alkynes catalyzed by a pyridonate borane complex is reported. While the catalyst can be described as an intramolecular frustrated Lewis pair, it is the change in the coordination mode of the pyridonate substituent upon H_2_ bond activation that enables dissociation of the formed pyridone borane complex and hydroboration as the first C−H bond‐forming step. This mode of action is referred to as boron‐ligand cooperation.

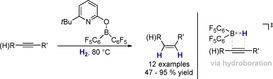

## Introduction

**Scheme 1:**
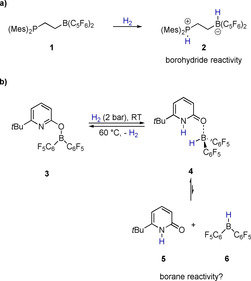
A classic intramolecular FLP that displays borohydride reactivity and reversible H_2_ activation by the boroxypyridine **3** that might display borane reactivity upon H_2_ activation and dissociation.

The seminal finding that specific combinations of sterically encumbered Lewis bases and Lewis acids, named „frustrated Lewis pairs“ (FLPs), can activate hydrogen, stimulated the development of catalytic metal‐free hydrogenations.^1^ Early examples included the hydrogenation of (di)imines, nitriles, aziridines, silyl enol ethers, and enamines, but the scope of FLP catalyzed hydrogenations was extended to heterocycles, alkenes, allenes, and aromatic hydrocarbons.^2^, ^3^ The heterolytic hydrogen cleavage by the FLP yields a tetravalent borohydride species. Therefore, hydrogenations by FLPs consist of a hydride and a subsequent proton transfer step (or *vice versa*) and require activated alkenes.^3^ A notable exception is the semihydrogenation of alkynes catalyzed by an intramolecular FLP that was reported by Repo et al.^4^ In that case, mechanistic investigations showed that the protolysis of the FLP under the reaction conditions yields an amine‐hydroborane that initiates the catalytic cycle by hydroboration of the alkyne.^5^ A protodeborylation of the alkenylborane yields then, in a highly stereoselective reaction, the *cis*‐alkene.^6^ We recently reported reversible H_2_ activation by the boroxypyridine **3**.^7^ A distinguishing feature of this system is that the H_2_ activation is associated with a transition of the covalently bound oxypyridine substituent to a neutral pyridone donor ligand (Scheme 1). This mode of action was, in analogy to the concept of metal‐ligand cooperation, termed boron‐ligand cooperation. The change in the coordination mode of the pyridone substituent might enable the dissociation of the pyridone borane complex **4** in the ligand 6‐*tert*‐butylpyridone **5** and Piers borane **6**. Piers borane has been shown to display the typical reactivity of a trivalent borane, for example, it effects the hydroboration of alkenes and alkynes. Such dissociation is not possible for classic FLPs that, as aforementioned, therefore rather display borohydride reactivity upon H_2_ activation (Scheme 1).

## Results and Discussion

**Scheme 2:**
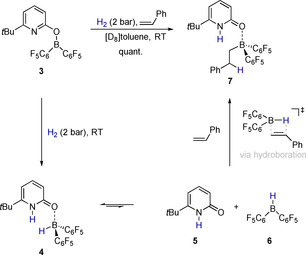
Hydroboration of styrene upon H_2_ activation by **3**.

We envisioned the hydroboration of an alkene to be a valid test reaction to elucidate whether **3** displays borane reactivity upon hydrogen activation, since hydroboration requires the presence of a trivalent borane. Indeed, when **3** was reacted with one equivalent of styrene under moderate H_2_‐pressure at RT, the formation of the alkyl borane **7** was observed (Scheme 2). The alkylborane **7** is also formed when styrene is reacted with the pyridone borane **4**, which supports the assumption that **4** is an intermediate in the formation of **7** starting from **3**.

**Scheme 3:**
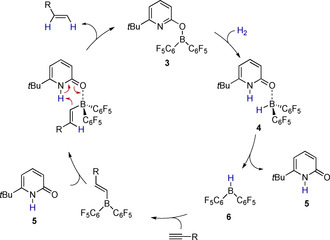
Envisioned mechanism of the hydrogenation of alkynes catalyzed by **3**: H_2_ activation yields the pyridone borane complex **4** that undergoes a dissociation. Piers borane **6** hydroborates an alkyne, formation of the pyridone alkenylborane complex and its protolysis are closing the catalytic cycle.

The alkylborane **7** does not undergo a protodeborylation. However, we envisioned that an analogous alkenylborane, originating from a reaction sequence consisting of H_2_ activation and hydroboration of an alkyne might succumb to protonolysis. This reaction would regenerate the boroxypyridine **3** and close a catalytic cycle for the hydrogenation of alkynes that consists of H_2_ activation by **3**, hydroboration of an alkyne and protonolysis of the alkenylborane (Scheme 3).

**Scheme 4:**
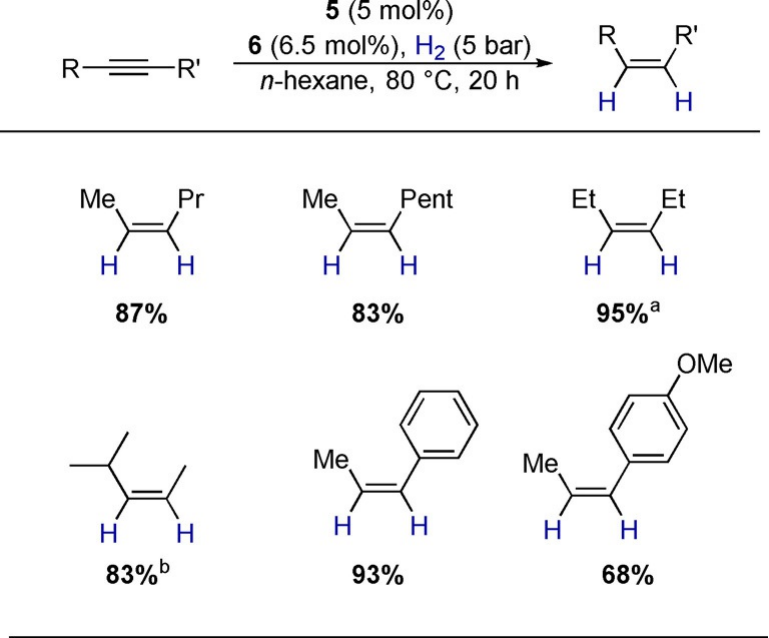
Substrate scope of the semihydrogenation of internal alkynes. Yields were determined by ^1^H NMR with trimethoxybenzene as internal standard and are given as the average of two runs a) 8 h reaction time; b) 16 h reaction time.

Indeed, 2‐hexyne was stereoselectively converted to *cis*‐2‐hexene in 87 % yield in the presence of catalytic amounts of **4** at 80 °C under 5 bar H_2_ pressure (Scheme 4). The catalyst **4** was generated in situ by coordination of **5** to Piers borane **6**. An initial screening of reaction conditions showed that a slight excess of Piers borane **6** (1.3 equivalents with respect to **5**) is beneficial to obtain reproducible good yields. Under the same conditions, *cis*‐2‐octene is obtained in very good yields from the hydrogenation of 2‐octyne. Likewise, *cis*‐3‐hexene is formed upon hydrogenation of 3‐hexyne in excellent yield after only 8 h reaction time. The hydrogenation of 4‐methyl‐2‐pentyne leads to the corresponding *cis* alkene in a very good yield after 16 h reaction time. Upon hydrogenation of the respective alkyne, 1‐phenyl‐1‐propene is obtained in an excellent yield of 93 %. Ethers are suitable substrates, as proven by the successful hydrogenation of 1‐(*para*‐methoxyphenyl)‐propyne.

**Scheme 5:**
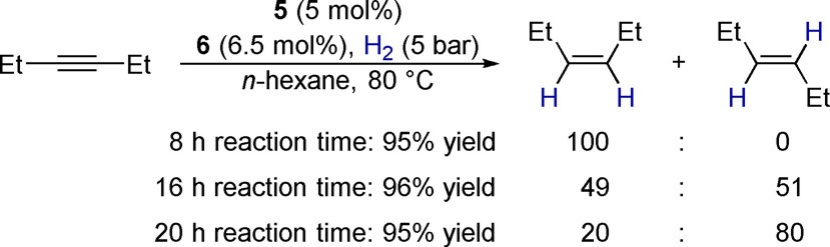
Stereoselectivity of the hydrogenation of 3‐hexyne in dependence of the reaction time.

While 3‐hexyne is obtained after 8 h exclusively as *cis* isomer, a prolonged reaction time of 16 h led to a 1:1 mixture of the *cis* and the *trans* isomer (Scheme 5). After 20 h, the *trans* isomer is the major product. Liu et al. reported that Piers borane can isomerize *cis*‐alkenes via reversible hydroboration.^5^ We, therefore, assume that the catalytic reaction yields first *cis*‐3‐hexene that is then subsequently isomerized by the Piers borane **6** that is present in the reaction mixture. Thus, both stereoisomers are accessible with the catalytic protocol described herein.

**Scheme 6:**
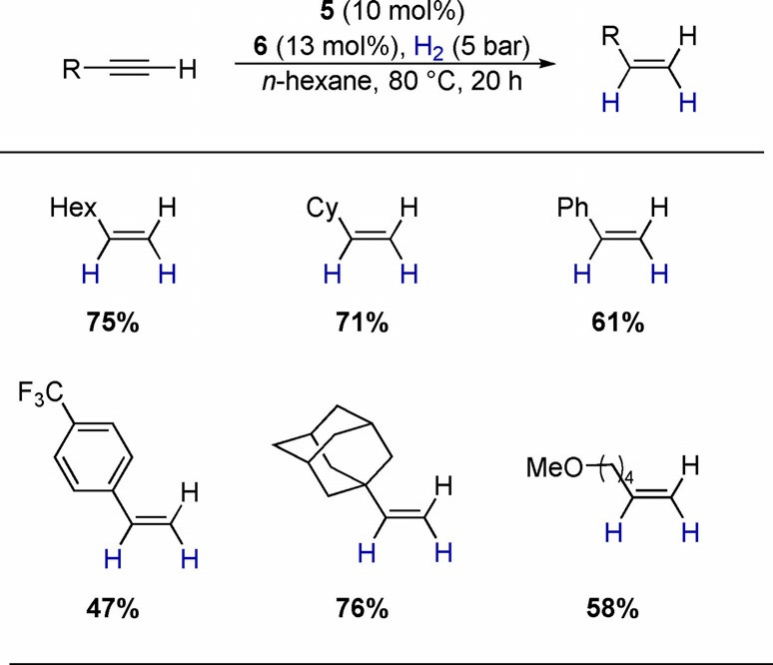
Substrate scope of the semihydrogenation of terminal alkynes. Yields were determined by ^1^H NMR with trimethoxybenzene as internal standard and are given as the average of two runs.

The known metal‐free protocols for the hydrogenation of alkynes are limited to internal alkynes. We were pleased to find that the catalyst described herein is capable to hydrogenate 1‐octyne in good yield with a catalyst loading of 10 mol % (Scheme 6). The catalytic protocol can also be used for the hydrogenation of other aliphatic alkynes such as cyclohexyl‐ and adamantly acetylene. While aromatic rings are tolerated, the hydrogenation of phenylacetylene and *para*‐(trifluoromethyl)phenylacetylene yielded the corresponding alkenes in lower yields. Again, ethers are suitable substrates, as demonstrated by the hydrogenation of 6‐methoxy‐1‐hexylacetylene.

**Scheme 7:**
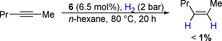
Attempted hydrogenation with Piers borane **6** as the catalyst.

With these results in hand, we aimed for a mechanistic understanding of the catalytic reaction. To verify that the pyridone **5** is indeed vital for the reaction, we attempted the hydrogenation of 2‐hexyne only with Piers borane **6** as catalyst (Scheme 7). Less than 1 % product was formed under reaction conditions that are identical to those reported in Scheme 4, clearly indicating that the presence of the pyridone **5** is essential for the reaction outcome.

**Scheme 8:**
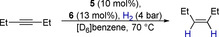
NMR monitoring of the catalytic hydrogenation of 3‐hexyne.

We then focused on the identification of the resting state of the catalytic reaction. For this purpose, the catalytic hydrogenation of 3‐hexyne was monitored by NMR (Scheme 8). Under 4 bar H_2_‐pressure, rapid formation of *cis*‐3‐hexene was observed at 70 °C in [D_6_]benzene, which implies that the observations made by this experiment are meaningful regarding the catalytic transformation.

**Figure 1:**
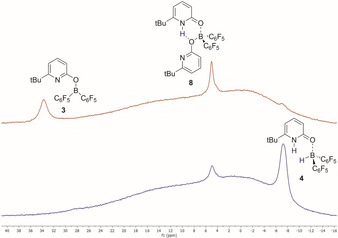
^11^B NMR spectra (193 MHz, [D_6_]benzene) obtained by monitoring of the catalytic reaction (Scheme 8) before heating (blue) and after 15 h at 60 °C (red).

The bispyridone complex **8** that was previously described and characterized in detail was observed by ^1^H NMR as the resting state of the catalytic reaction (Figure 1).^8^ Furthermore, ^1^H and ^11^B NMR proved formation of boroxypyridine **3** with progressing reaction and hydrogen consumption. This finding strongly supports the assumption that **3** is part of the catalytic cycle.^9^

**Scheme 9:**
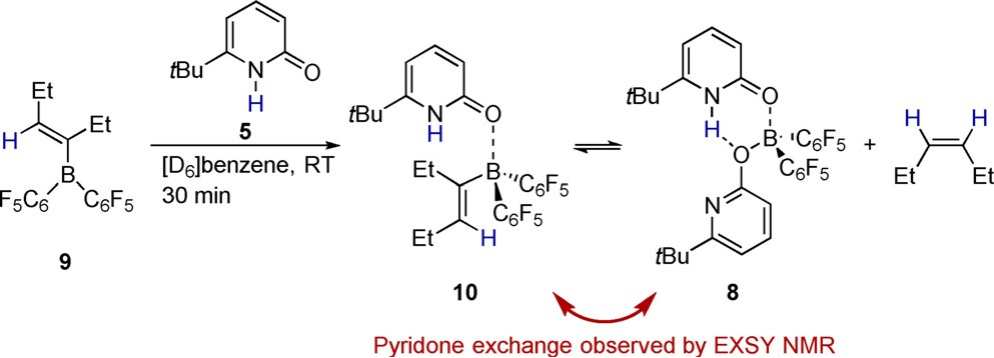
Stoichiometric reaction of the alkenylborane **9** with the *tert*‐butylpyridone **5**.

To elucidate whether the envisioned protonolysis of the alkenylborane can be assumed to be part of the catalytic reaction, **5** was added to the borane **9**, derived from the reaction of Piers borane **6** and 3‐hexyne. The reaction progress at RT was monitored by NMR spectroscopy (Scheme 9). Within 30 minutes, the formation of the expected pyridone alkenylborane complex **10** was observed. Furthermore, signals that were assigned to *cis*‐3‐hexene, the product of the protonolysis, were detected. The presence of *cis*‐3‐hexene implies that boroxypyridine **3**, originating from the protonolysis must be present. Indeed, the formation of the bispyridone complex **8** that contains one equivalent of **3** was observed.

**Figure 2:**
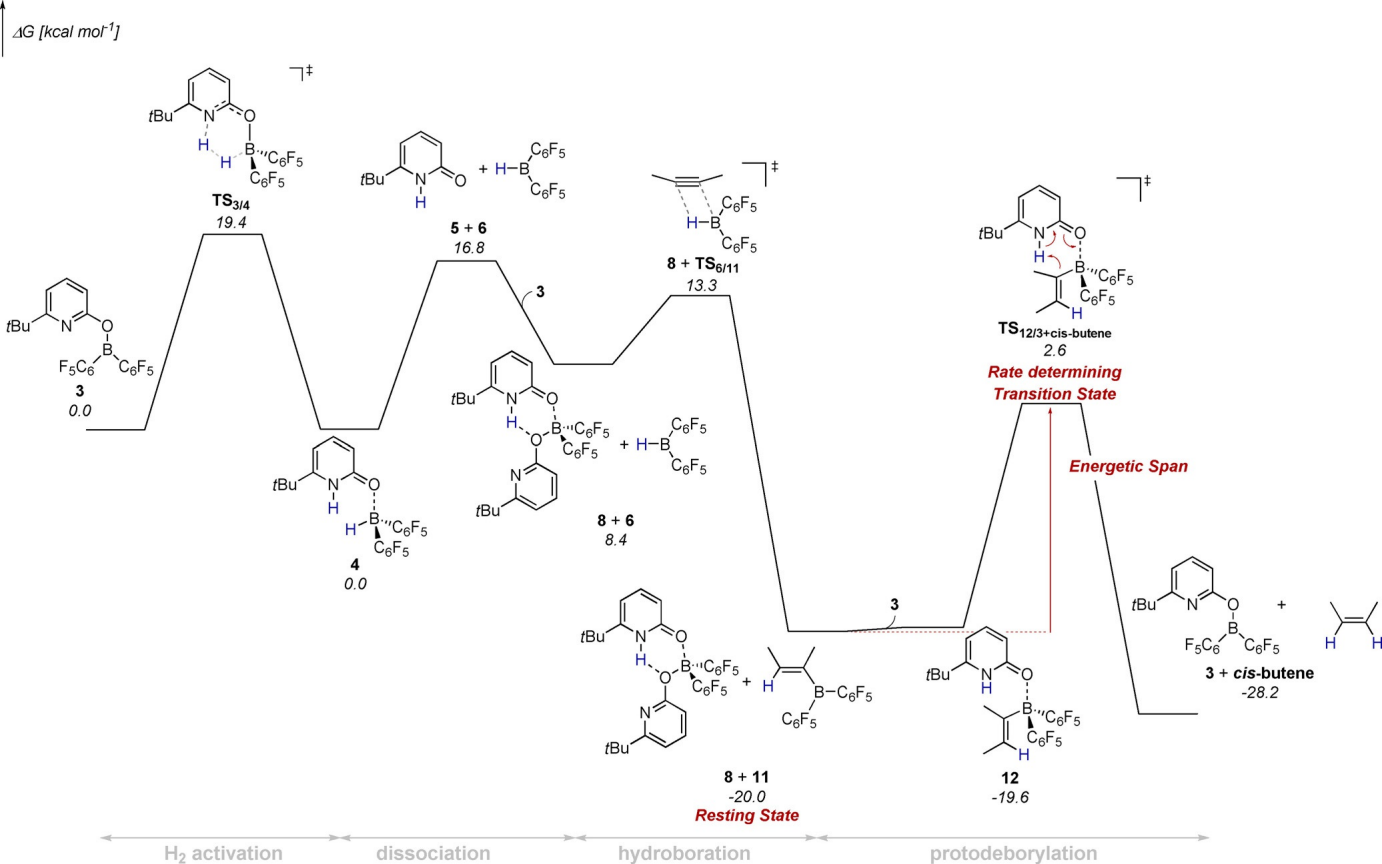
Gibbs free energy profile for the hydrogen activation by **3** computed at revDSD‐PBEP86‐D4/def2‐QZVPP//PBEh‐3c. Bulk solvation was considered implicitly with the SMD model for hexane.

EXSY NMR spectroscopy shows an exchange of the pyridone **5** between **10** and **8** at RT, which further supports that **8** is not an unreactive, irreversibly formed species but rather a resting state. The mechanism of the catalytic reaction was further investigated computationally at revDSD‐PBEP86‐D4/def2‐QZVPP//PBEh‐3c (Figure 2).^10^, ^11^ The SMD model for *n*‐hexane was used to implicitly account for solvent effects.^12^ The hydrogen activation by **3** requires a free activation energy of 19.4 kcal mol^−1^. This elementary step is according to our computations thermoneutral, which agrees with the previously observed facile reversibility of the hydrogen activation.^7^ The free energy change that is associated with the dissociation of **4** into Piers borane **6** and the pyridone **5** is 16.8 kcal mol^−1^. Relaxed potential energy surface scans indicate that the dissociation is barrierless. As the experimental results indicate that the bispyridone complex **8** is the resting state of the transformation, we considered the coordination of the free pyridone **5** to the boroxypyridine **3**. Indeed, the formation of **8** is according to the computations exergonic. The hydroboration of the model substrate 2‐butyne requires a moderate activation energy of 4.9 kcal mol^−1^ and yields the alkenylborane **11**. The bispyridone complex **8** together with **11** is the resting state of the catalytic transformation.^13^ The pyridone **5**, that is bound in complex **8**, coordinates than to **11** forming the pyridone alkenylborane complex **12**.

**Scheme 10:**
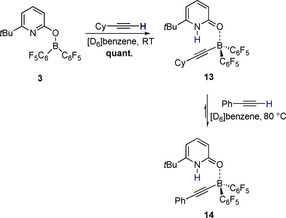
C_sp_−H cleavage of cyclohexylacetylene by **3** and exchange with phenylacetylene upon heating to 80 °C.

Note that pyridone exchange between **8** and the pyridone alkenylborane complex **10** was observed experimentally by EXSY NMR. The activation barrier for the protodeborylation is 22.2 kcal mol^−1^, which corresponds to a half‐life time of **12** of 35.8 minutes at 25 °C.^14^ This agrees with the experimental observation that the protodeborylation takes place at RT (Scheme 9). The „Energetic Span“, that is the kinetic barrier of the catalytic transformation, is between the resting state (**8** and **11**) and the transition state of the protodeborylation.^15^ Classic FLP type catalysts are not suitable for the hydrogenation of terminal alkynes, presumably because they are deactivated by an irreversible C_sp_−H cleavage.^3^ To understand why the catalyst system described herein tolerates terminal alkynes, **3** was reacted with cyclohexylacetylene at RT. As previously reported, this reaction led to the formation of the alkynylborane complex **13** (Scheme 10).^16^ Upon addition of phenylacetylene and heating to 80 °C, **13** was partially converted to the phenylalkynylborane complex **14**.

**Figure 3:**
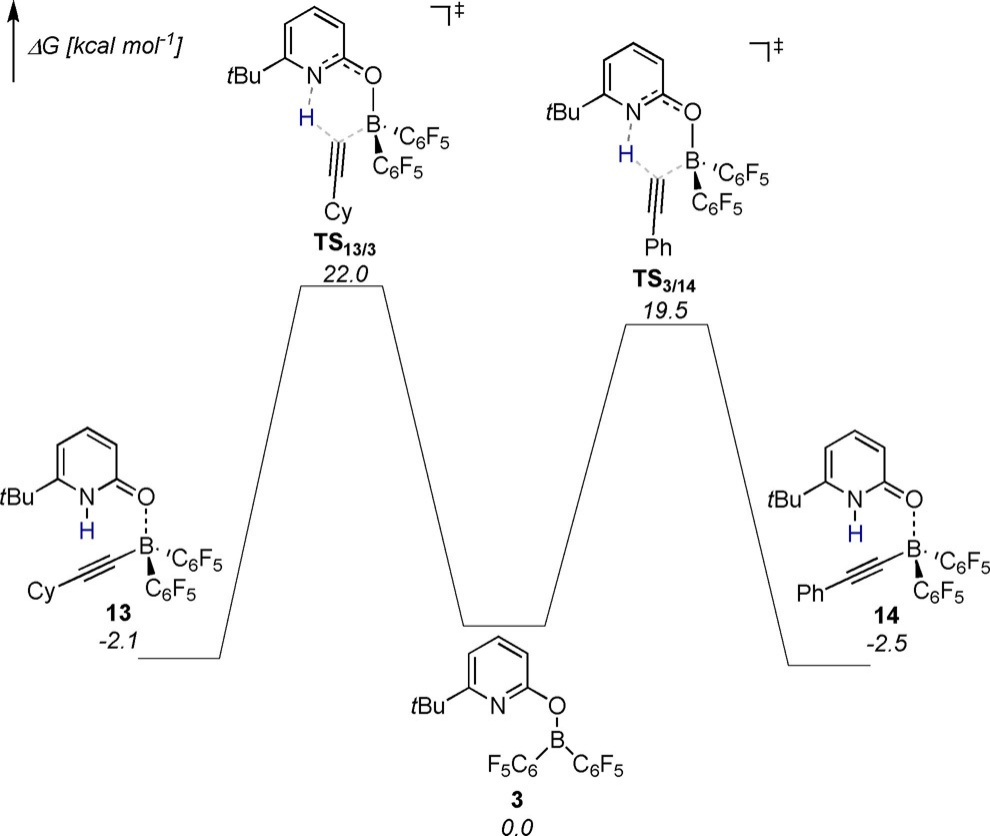
Gibbs free energy profile for the C_sp_−H activation of cyclohxylacetylene and phenylacetylene by **3** computed at revDSD‐PBEP86‐D4/def2‐QZVPP//PBEh‐3c. Bulk solvation was considered implicitly with the SMD model for hexane.

After 1 h at 80 °C, the ratio of **14** to **13** was 4:1. This experiment indicates that the C_sp_−H cleavage is reversible under the reaction conditions. The assumption that the formation of the alkynylborane is reversible is further supported by DFT computations (Figure 3). According to the computations, the liberation of cyclohexyacetylene from **13** requires a free Gibbs activation energy of 24.1 kcal mol^−1^, which corresponds to a half‐life time of 79 seconds at 80 °C. The formation of the phenyl alkynyl borane complex **14** is kinetically and thermodynamically favored.

The computed Gibbs free energy difference of 0.4 kcal mol^−1^ corresponds to a ratio of 2:1, which is in reasonable agreement with the experimentally observed proportion of the two alkynyl borane complexes. It is certainly the reversibility of the C_sp_−H cleavage that allows H_2_ activation in the presence of terminal alkynes and thus the first metal‐free hydrogenation of terminal alkynes.

## Conclusions

We have documented the efficient semihydrogenation of internal and terminal alkynes by a boroxypyridine that displays frustrated Lewis pair reactivity and is, therefore, able to activate hydrogen. However, the change in the coordination mode of the pyridonate substituent enables hydroboration as the initial step of the hydrogenation and is thus vital for the catalytic reaction. We expect this finding to pave the way for novel metal‐free catalytic reactions that rely on this mode of action.

## Experimental Section

**General Procedure for hydrogenation of alkynes**: Piers borane **6** (13.5 mg, 0.039 mmol) and 6‐*tert*‐butyl‐2‐pyridone **5** (4.5 mg, 0.030 mmol) were dissolved in *n*‐hexane (5 mL) in a Fisher‐Porter type 150 mL reaction vessel equipped with a stirring bar. The respective alkyne (0.60 mmol or 0.30 mmol) was added. The reaction vessel was closed and connected to an H_2_ bomb with a gas hose. The hose was rinsed with H_2_ several times and the reaction vessel pressurized with H_2_ (5 bar). The reaction vessel was placed inside an 80 °C preheated oil bath and stirred at 1000 rpm. After 20 h, the reaction mixture was cooled to room temperature and the excess H_2_ gas was released. An aliquot was taken, and the yield determined by ^1^H NMR using 1,3,5‐trimethoxybenzene as internal standard.

## Conflict of interest

The authors declare no conflict of interest.

## Supporting information

As a service to our authors and readers, this journal provides supporting information supplied by the authors. Such materials are peer reviewed and may be re‐organized for online delivery, but are not copy‐edited or typeset. Technical support issues arising from supporting information (other than missing files) should be addressed to the authors.

Supplementary
