# Supplementary material for: Semihydrogenation of Alkynes Catalyzed by a Pyridone Borane Complex: Frustrated Lewis Pair Reactivity and Boron–Ligand Cooperation in Concert
Source: Chemistry. 2020 Sep 18;26(59):13445–50. doi: 10.1002/chem.202001276 (PMC7693047; doi:10.1002/chem.202001276)
Supplement: Supplementary file 1 — Supplementary [file CHEM-26-13445-s001.pdf]

Chemistry–A European Journal

Supporting Information

Semihydrogenation of Alkynes Catalyzed by a Pyridone Borane Complex: Frustrated Lewis Pair Reactivity and Boron–Ligand Cooperation in Concert

Felix Wech, Max Hasenbeck, and Urs Gellrich*[a]

Table of contents

1 General Specifications 2

2 General procedure for the hydrogenation of alkynes 3

3 Mechanistic investigations 4

3.1 Consecutive H_2 activation and hydroboration of styrene starting from boroxypyridine **3** 4

3.2 Hydroboration of styrene starting from pyridone borane **4** 10

3.3 Monitoring of the hydrogenation of 3-hexyne via NMR spectroscopy 16

3.4 Reaction of (*Z*)-hex-3-en-3-ylbis(perfluorophenyl)borane with pyridone **5** 26

3.5 Independent synthesis and characterization of the hydroboration product of 3-hexyne with Piers borane, (*Z*)-hex-3-en-3-ylbis(pentafluorophenyl)borane **9** 44

3.5.1 Additional NMR spectra 45

3.6 Characterization of (*Z*)-hex-3-en-3-ylbis(perfluorophenyl)borane pyridone complex **10** 52

3.6.1 Additional NMR spectra 53

3.7 Independent synthesis of the hydroboration product of styrene and Piers borane 55

3.7.1 Additional NMR spectra 56

3.8 Synthesis of the hydroborated styrene's 6-*tert*-2-pyridone complex **7** 59

3.8.1 Additional NMR spectra 60

3.9 Investigation of the C–H Activation of terminal alkynes by the boroxypyridine **3** 64

4 NMR spectra of the catalysis experiments 67

5 Tuning the *E*/*Z* by changing the reaction time 94

5.1 Additional NMR spectra 95

6 Computational details 99

7 References 118

1 General Specifications

All manipulations of air and moisture sensitive compounds were carried out under a nitrogen atmosphere using standard Schlenk and glovebox techniques.

Pyridone **5** was synthesized from 3,3'-dimethyl-2-butanone according to a literature procedure.[1]

$B(C_6F_5)_3$ was synthesized from boron trifluoride etherate according to a literature procedure.[2]

Piers borane **6** was synthesized from $B(C_6F_5)_3$ and Et_3SiH according to a modified literature procedure.[3] $B(C_6F_5)_3$ (2.0 g, 3.9 mmol) and Et_3SiH (623 µL, 3.9 mmol) were dissolved in benzene (20 mL) in a reactor bomb and stirred for 4-5 d at 60 °C. The reaction mixture was cooled to r.t., causing precipitation of the product. The supernatant was removed, and the residue was washed 2-3 times with 3 mL benzene and two times with 2-3 mL *n*-pentane.

6-Methoxy-1-hexyne was synthesized according to a literature procedure.[4]

1-(*para*-methoxyphenyl)-1-propyne was synthesized from *p*-methoxyphenylacetylene according to a literature procedure.[5]

Ad-C≡CH was synthesized according to a modified literature known procedure where DMSO was used as a solvent and the solid was sublimated for purification at 60-100 °C under high vacuum.[6]

All dry, non-deuterated solvents were, if commercially available, purchased from Acros Organics or Sigma Aldrich in a sealed bottle with a septum and stored (except benzene) over molecular sieves.

Deuterated solvents were distilled under inert conditions and kept in the glovebox over 4 Å molecular sieves.

The internal alkynes were commercially purchased and, if liquid, distilled under inert conditions or subjected to three freeze-pump-thaw cycles, and stored at least one day over molecular sieves in a glovebox.

NMR spectra were recorded on a Bruker Avance II 200 MHz, Bruker Avance III HD 400 MHz, Bruker Avance II 400 MHz and Bruker Avance III HD 600 MHz spectrometers. ^{1}H and ^{13}C NMR chemical shifts are referenced to residual solvent resonance peaks.

2 General procedure for the hydrogenation of alkynes

Piers borane **6** (13.5 mg, 0.039 mmol) and pyridone **5** (4.5 mg, 0.030 mmol) were dissolved in *n*-hexane (5 ml) in a Fisher-Porter type 150 mL reaction vessel equipped with a stirring bar. The respective alkyne (0.60 mmol internal alkyne or 0.30 mmol terminal alkyne) was added. In the case of the blind experiment, the reaction was set up using Piers borane **6** only. The reaction vessel was connected to an H_2 bomb with a gas hose. The hose was rinsed with H_2 several times and the reaction vessel was pressurized with H_2 (5 bar). It was placed inside an oil bath preheated to 80 °C and stirred for 20 h unless stated otherwise at 1000 rpm. After the reaction the vessel was cooled to room temperature, the excess pressure was released and 1,3,5-trimethoxybenzene (8.4 mg, 0.05 mmol) was added. An aliquot was transferred to an NMR tube containing a closed glass capillary filled with benzene-d_6 and a ^{1}H NMR spectrum was recorded. The amount of alkene was quantified by comparing the olefinic signals to the signals of 1,3,5-trimethoxybenzene.

3 Mechanistic investigations

Benzene-d_6 was used for mechanistic NMR experiments. Therefore 2-hexyne was also hydrogenated under standard conditions with benzene as solvent to demonstrate comparability (Figure SI 61 and Figure SI 62).

3.1 Consecutive H_2 activation and hydroboration of styrene starting from boroxypyridine 3

To show that after H_2 activation of boroxypyridine **3**, pyridone borane **4** exhibits borane reactivity the H_2 activation of **3** in the presence of styrene was investigated.

Pyridone **5** (4.5 mg, 0.03 mmol) and Piers borane **6** (10.4 mg, 0.03 mmol) were dissolved in benzene-d_6 (0.4 ml), transferred to an NMR tube with J Young valve, freeze-pump-thawed three times, and heated to 60 °C overnight under passive vacuum. The ^1H NMR spectrum shows the formation of **3** (Figure SI 1).

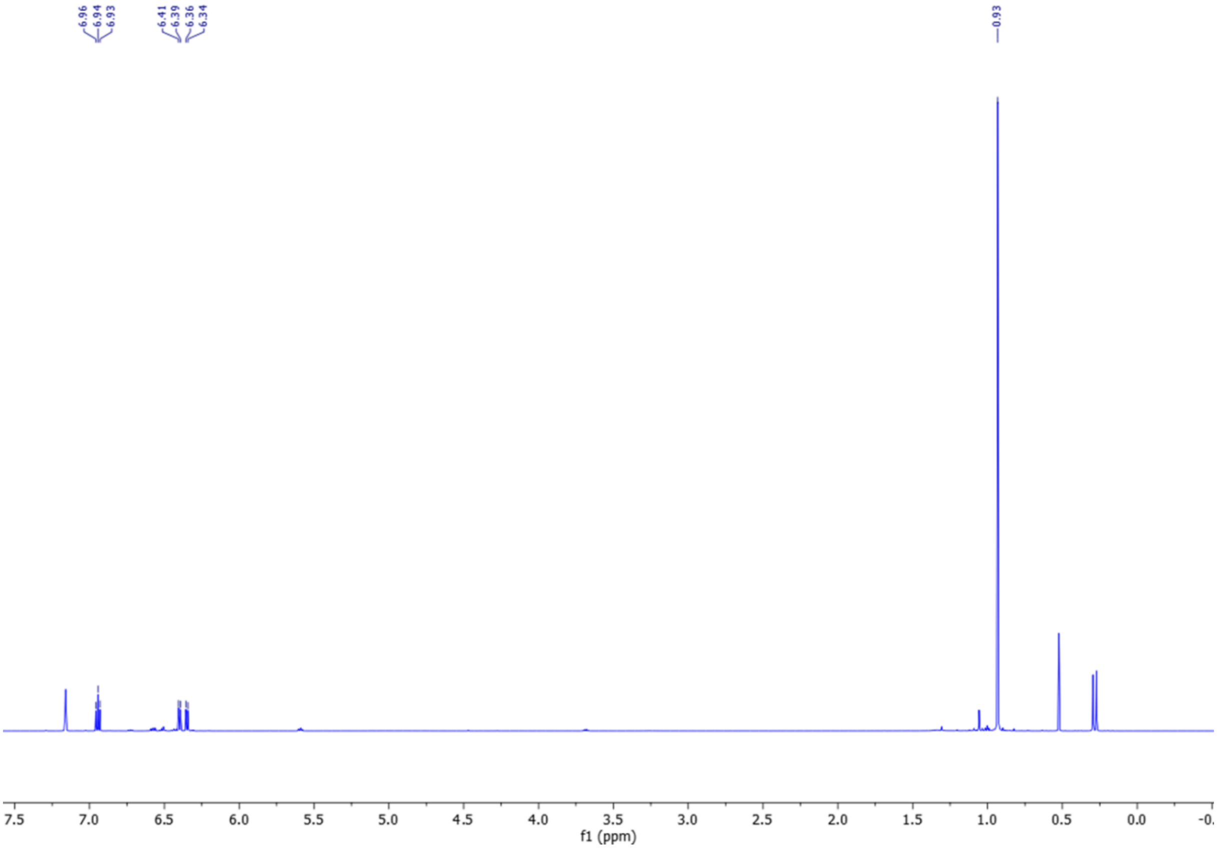

Figure SI 1: ^1H NMR spectrum of boroxypyridine **3** (red triangle) (600 MHz, benzene-d_6).

Styrene (3.4 µL, 0.03 mmol) was added (Figure SI 2 and Figure SI 3).

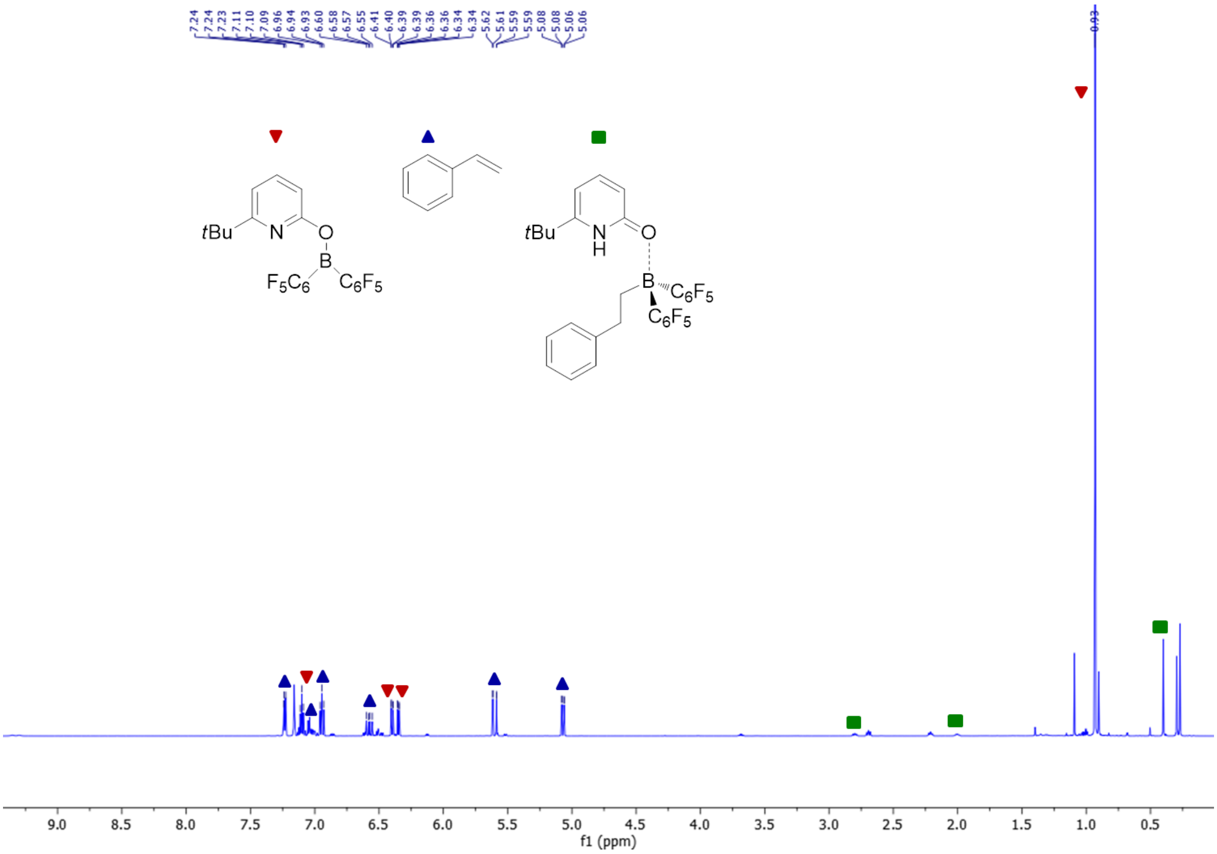

Figure SI 2: ^{1}H NMR spectrum after the addition of styrene to boroxypyridine **3** (600 MHz, benzene-d_6).

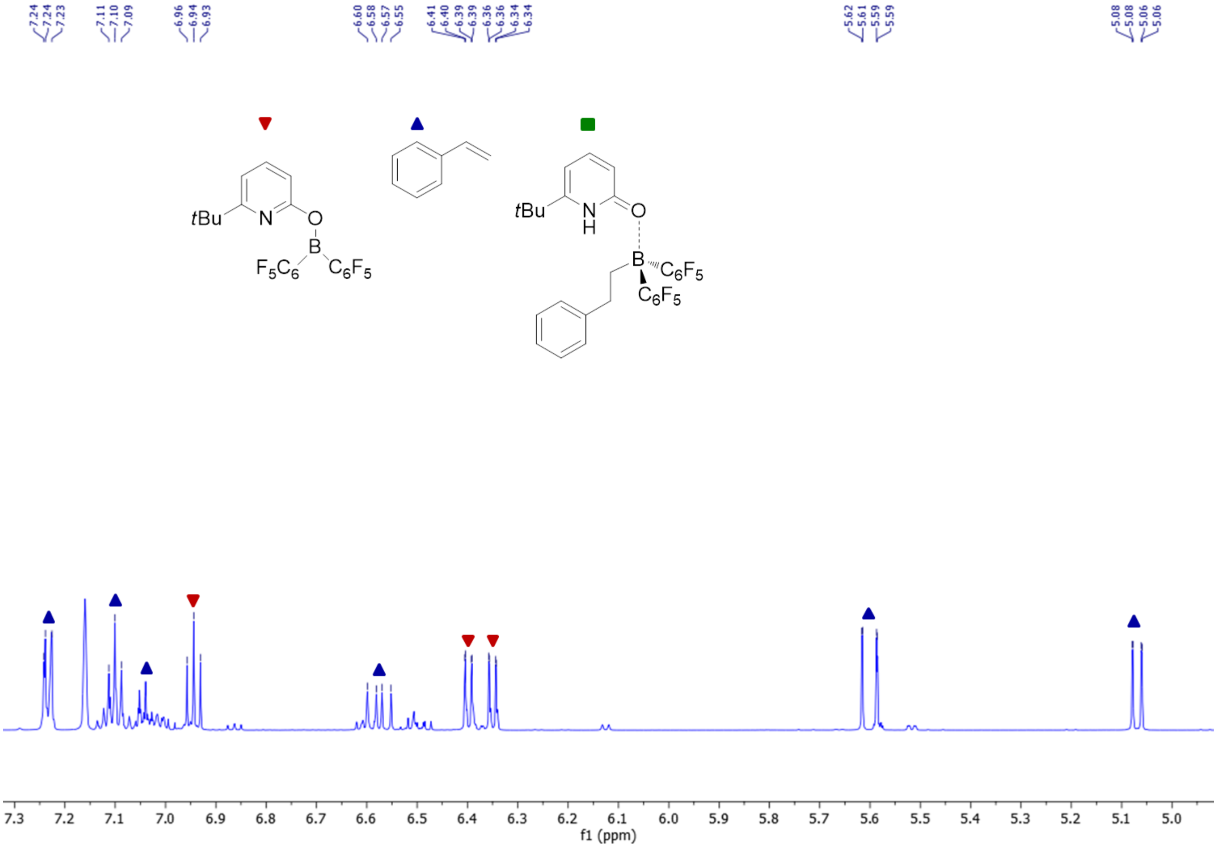

Figure SI 3: Excerpt of the ^{1}H NMR spectrum after the addition of styrene to boroxypyridine **3** (600 MHz, benzene-*d*$_6$).

The NMR tube was subject to three freeze-pump-thaw cycles and subsequently pressurized with H_2 (2.5 bar). The solution was kept at room temperature overnight and ^{1}H and ^{11}B NMR spectra were measured (Figure SI 4, Figure SI 5 and Figure SI 6).

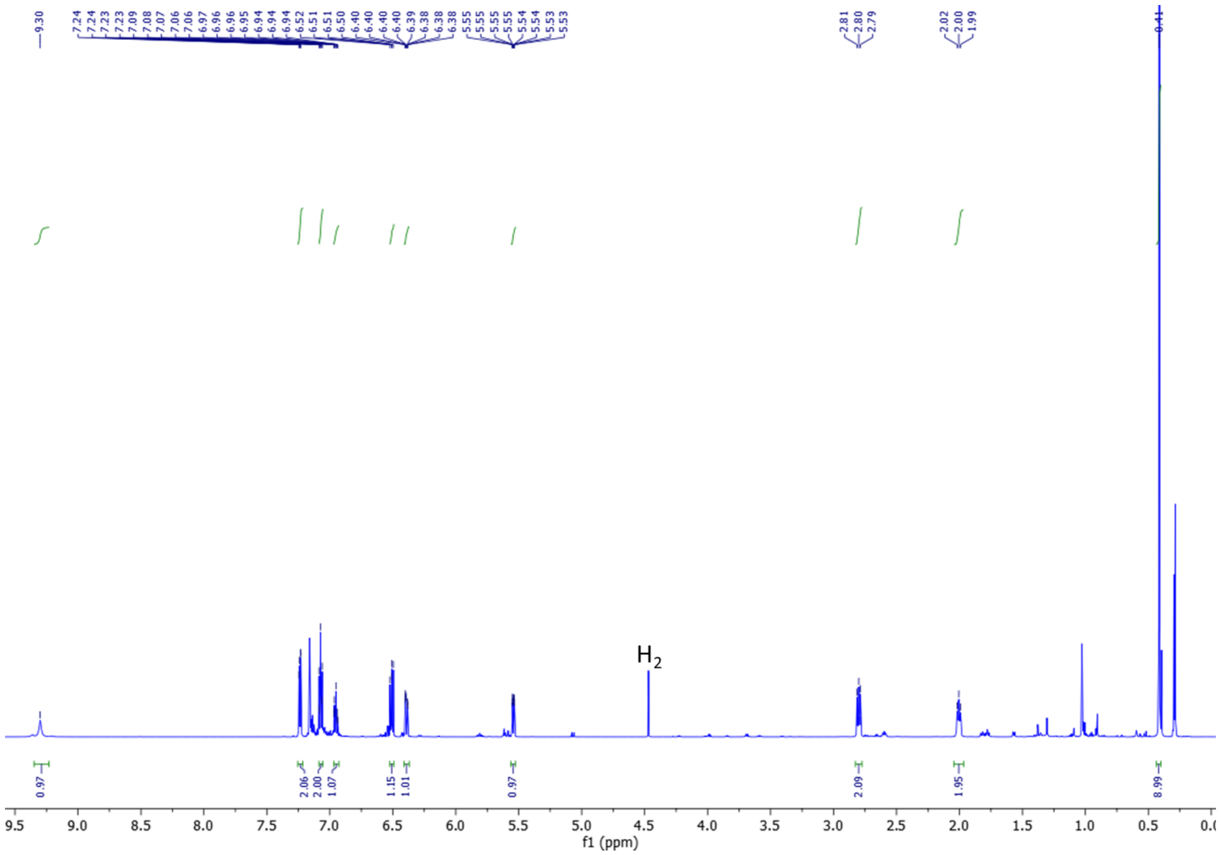

Figure SI 4: ^{1}H NMR spectrum after the solution of styrene and boroxypyridine **3** was pressurized with 2.5 bar of H_2 (600 MHz, benzene-d_6).

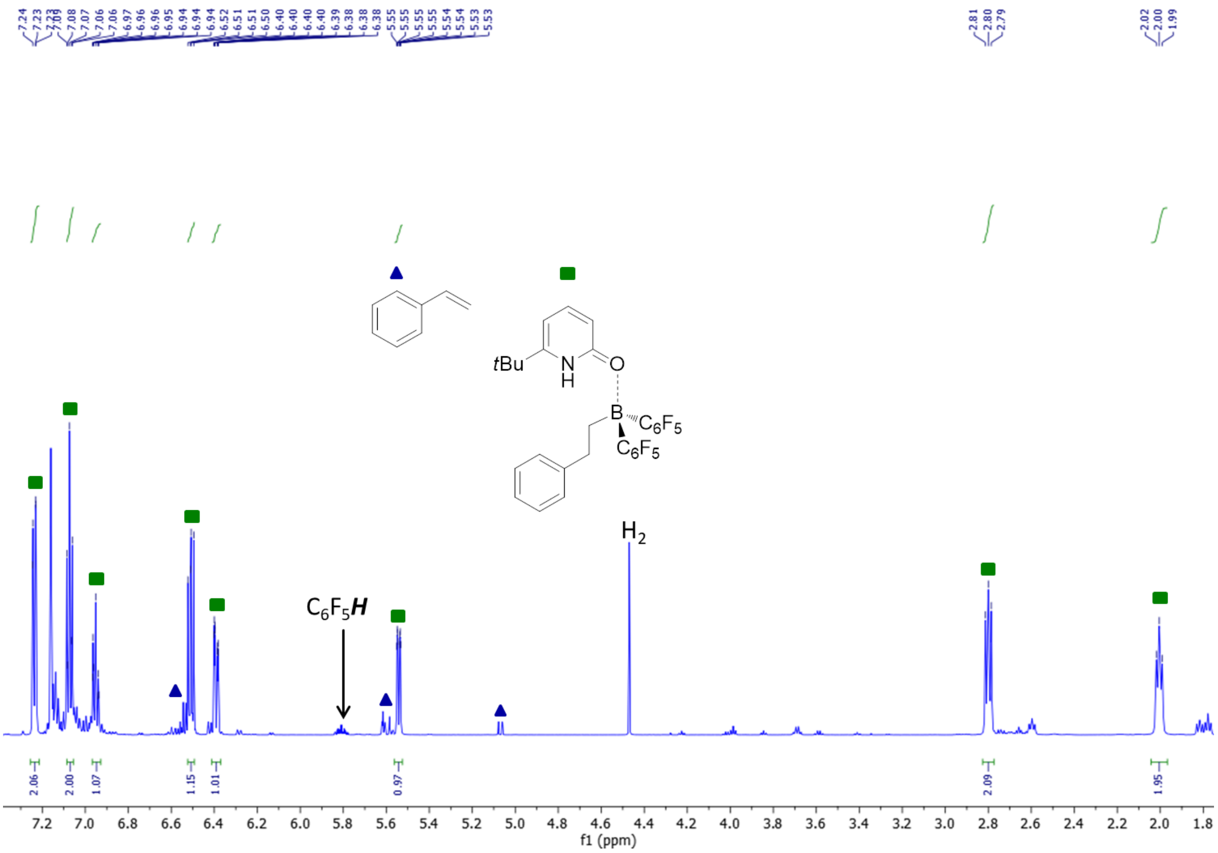

Figure SI 5: Excerpt of the ^{1}H NMR spectrum after the solution of styrene and boroxypyridine **3** was pressurized with 2.5 bar of H_2 (600 MHz, benzene-d_6).

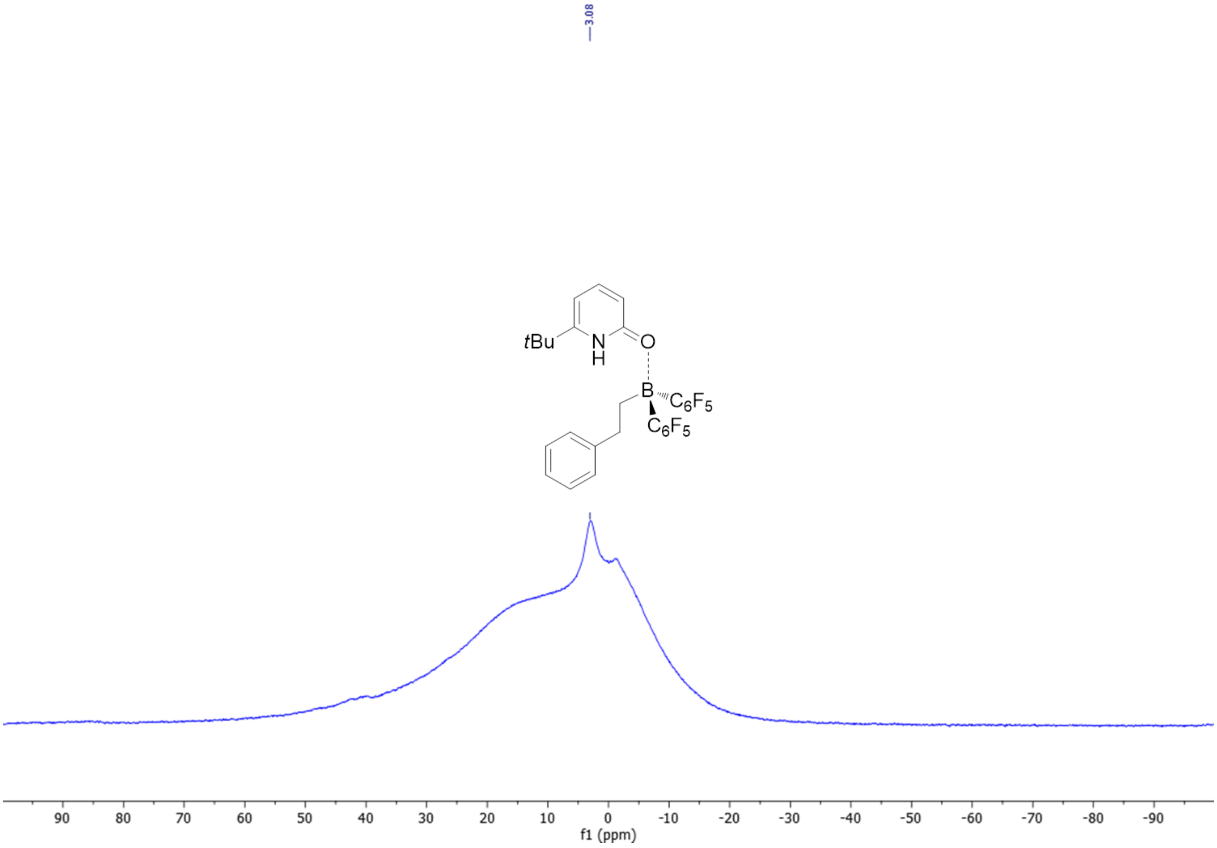

Figure SI 6: ^{11}B NMR spectrum after the solution of styrene and boroxypyridine **3** was pressurized with 2.5 bar of H_2 (193 MHz, benzene-d_6).

The main component in the reaction mixture is alkyl borane pyridone complex **7** (Figure SI 5 and Figure SI 6). The assignment is strongly supported by the independent synthesis of **7** (see chapter 3.8).

3.2 Hydroboration of styrene starting from pyridone borane 4

Pyridone **5** (4.5 mg, 0.03 mmol), Piers borane **6** (10.4 mg, 0.03 mmol), and styrene (3.43 µL, 0.03 mmol) were dissolved in toluene-d_8 (0.4 ml) in an NMR tube with J Young valve. The tube was freeze-pump-thawed three times and pressurized with H_2 (2.5 bar). After keeping the reaction solution at room temperature overnight ^{1}H and ^{11}B NMR spectra were measured (Figure SI 7, Figure SI 8, Figure SI 9, and Figure SI 10). The ^{1}H NMR spectra show three main components: styrene, bispyridone complex **8** and the alkyl borane pyridone complex **7**. The integrals of styrene and complex **7** have a ratio of 0.4 to 1 showing that about 71 % of styrene was hydroborated and is bound in complex **7**. The corresponding ^{11}B NMR spectrum shows two signals at 4.9 ppm and 2.9 ppm which were tentatively assigned to complex **8** and **7**.

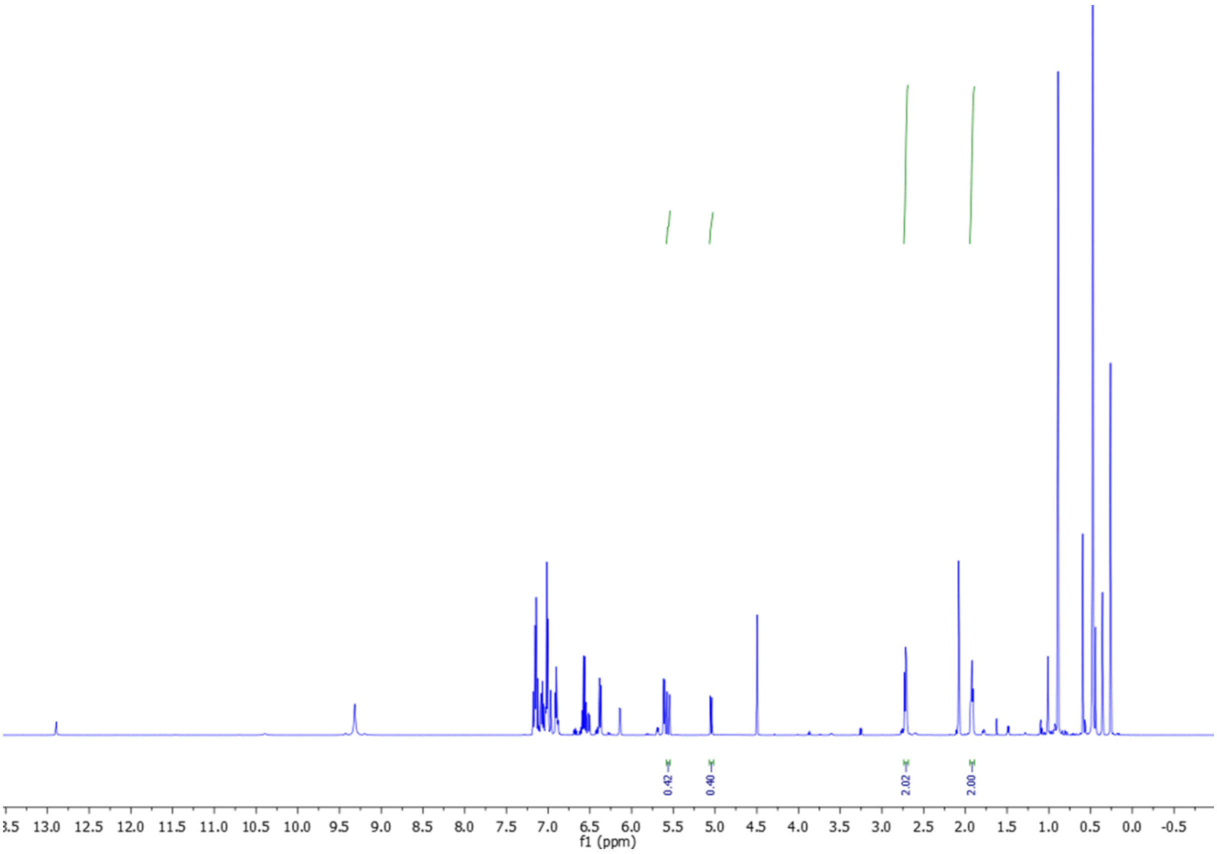

Figure SI 7: ^{1}H NMR spectrum of the reaction of pyridone borane **4** with styrene under H_2 pressure (2.5 bar) after overnight at room temperature (600 MHz, toluene-d_8).

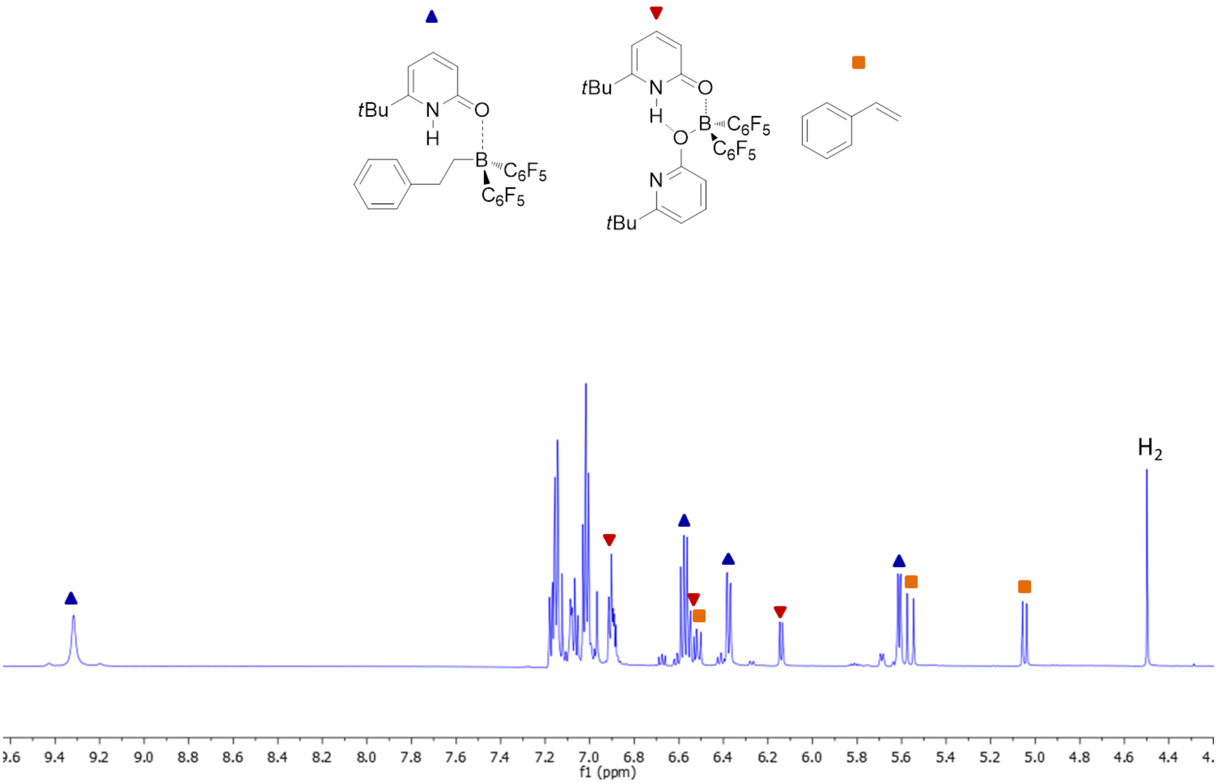

Figure SI 8: Low field excerpt of the ^1H NMR spectrum of the reaction of pyridone borane **4** with styrene under H_2 pressure (2.5 bar) after overnight at room temperature (600 MHz, toluene-*d*$_8$).

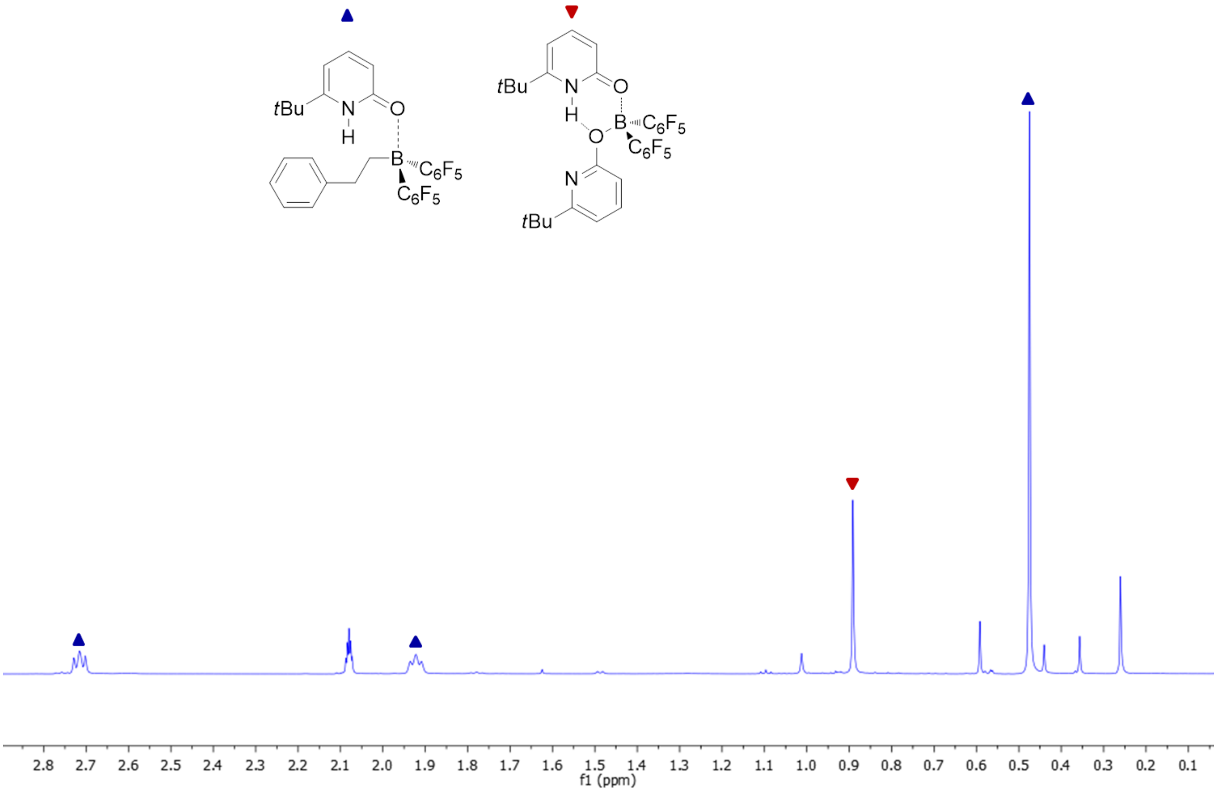

Figure SI 9: High field excerpt of the ^{1}H NMR spectrum of the reaction of pyridone borane **4** with styrene under H_2 pressure (2.5 bar) after overnight at room temperature (600 MHz, toluene-d_8).

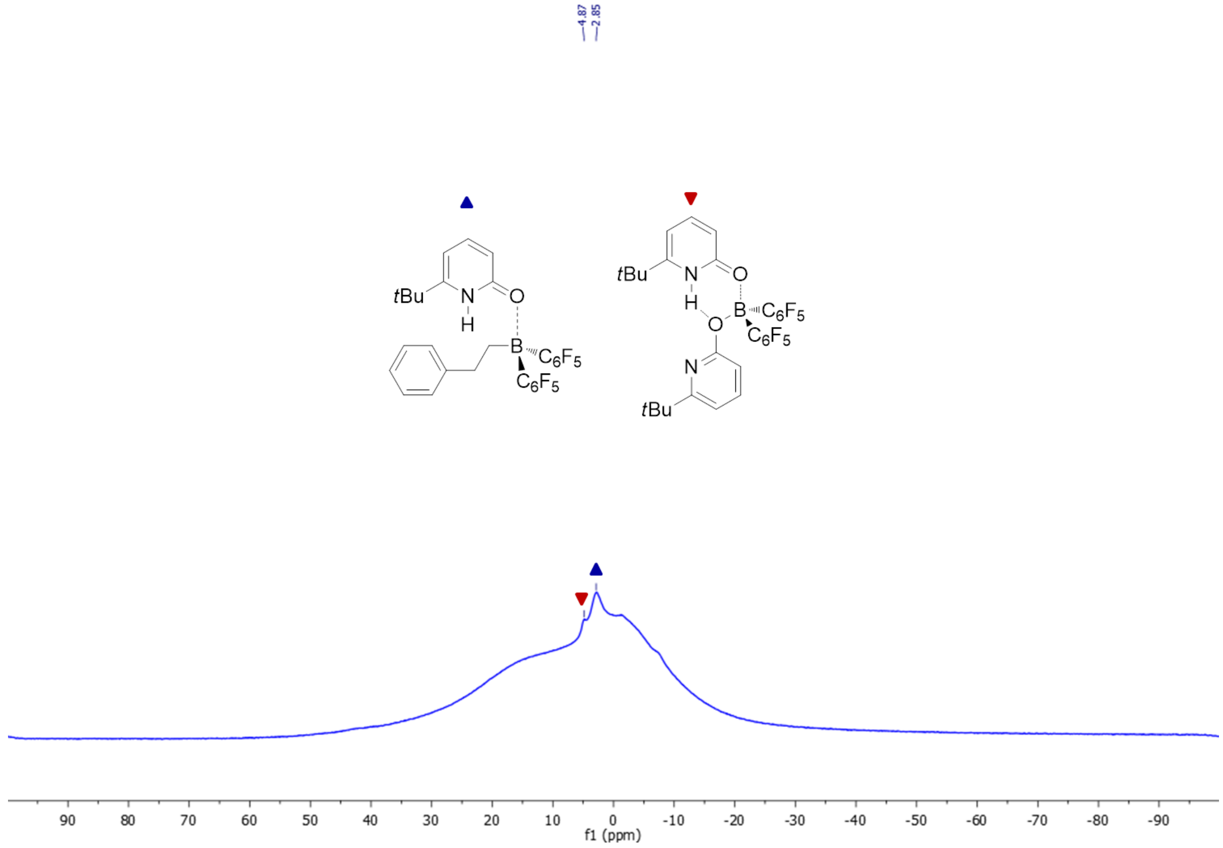

Figure SI 10: ^{11}B NMR spectrum of the reaction of pyridone borane **4** with styrene under H_2 pressure (2.5 bar) after overnight at room temperature (193 MHz, toluene-d_8).

After heating the reaction mixture to 120 °C for 4 h the formation of the hydrogenation product of styrene (ethylbenzene) was not observed, but rather the formation of pentafluorobenzene (Figure SI 11). The ^{11}B NMR spectrum shows a new signal at 43.2 ppm which was tentatively assigned to the analogue of complex **7** after pentafluorobenzene was eliminated (Figure SI 12).

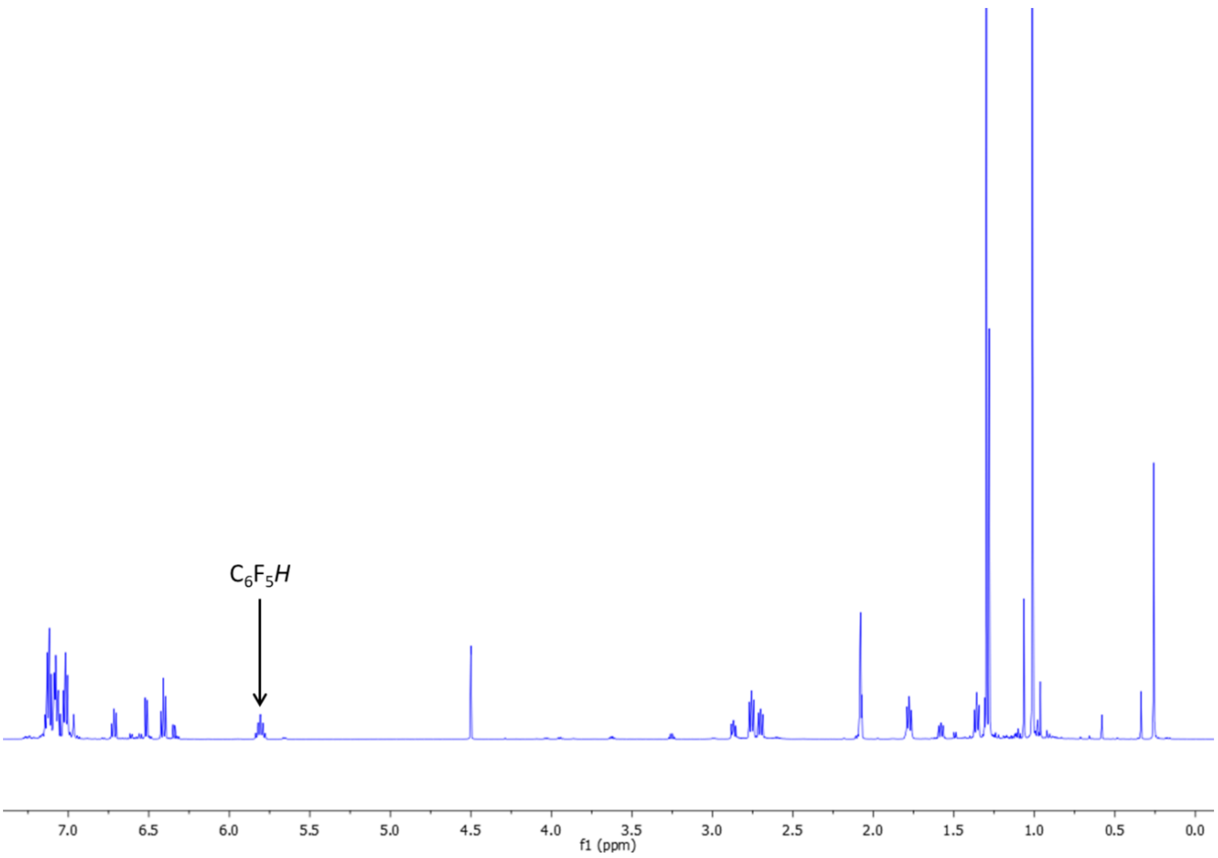

Figure SI 11: ^{1}H NMR spectrum of pyridone borane **4** with styrene under H_2 pressure after being heated to 120 °C for 4 h (600 MHz, toluene-d_8)

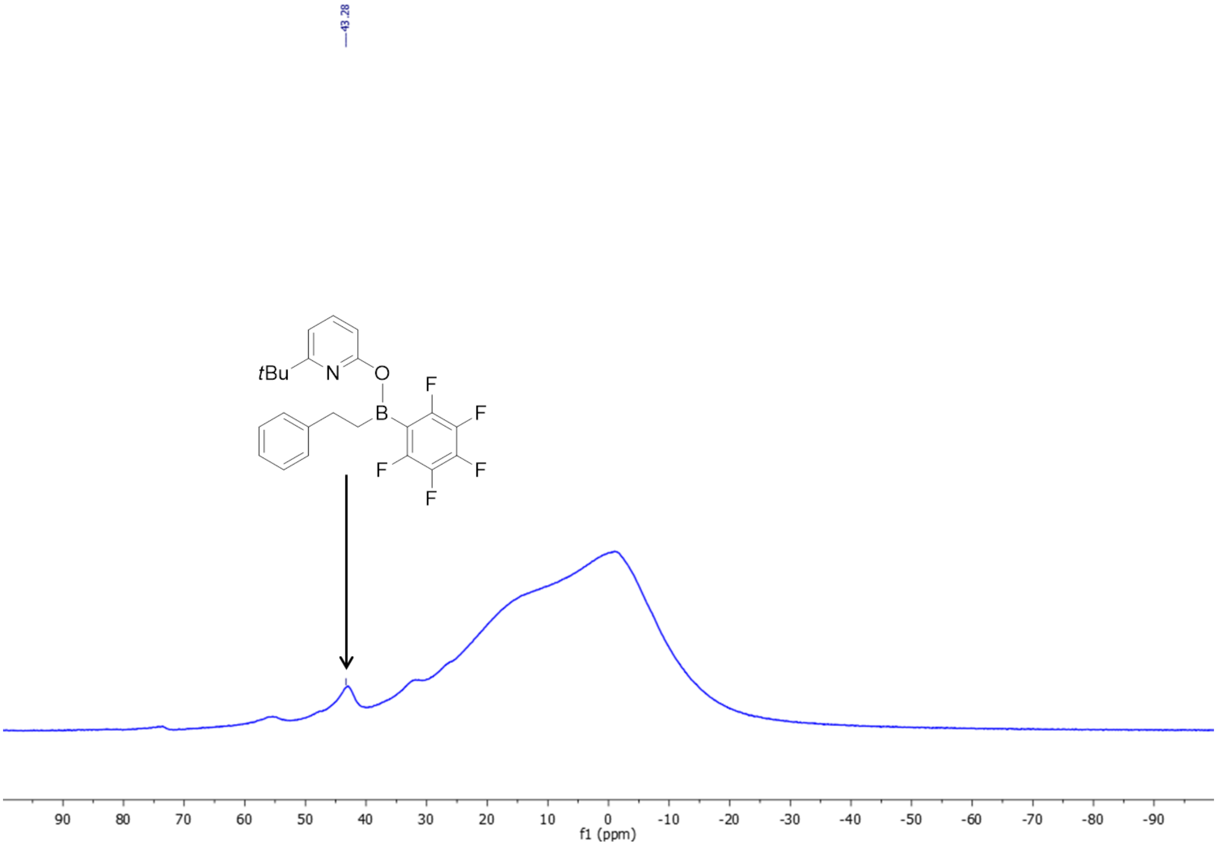

Figure SI 12: ^{11}B NMR spectrum of pyridone borane **4** with styrene under H_2 pressure after being heated to 120 °C for 4 h (193 MHz, toluene-d_8)

3.3 Monitoring of the hydrogenation of 3-hexyne via NMR spectroscopy

In an NMR tube with J Young valve pyridone **5** (4.5 mg, 0.030 mmol) and Piers borane **6** (13.5 mg, 0.039 mmol) were dissolved in benzene-d_6 (0.4 ml). After 10 min 3-hexyne (34.2 µL, 0.300 mmol) was added and the tube was subject to three freeze-pump-thaw cycles. The tube was subsequently pressurized with H_2 (4 bar) and ^1H and ^{11}B NMR spectra were recorded at room temperature.

The ^1H NMR spectrum shows four components: pyridone borane **4** (blue up-pointing triangle), the bispyridone complex **8** (red down-pointing triangle), the hydroboration product of 3-hexyne with Piers borane (grey dot) (in a 1:0.13:0.42 ratio), and 3-hexyne (orange square) (Figure SI 13, Figure SI 14, and Figure SI 15).

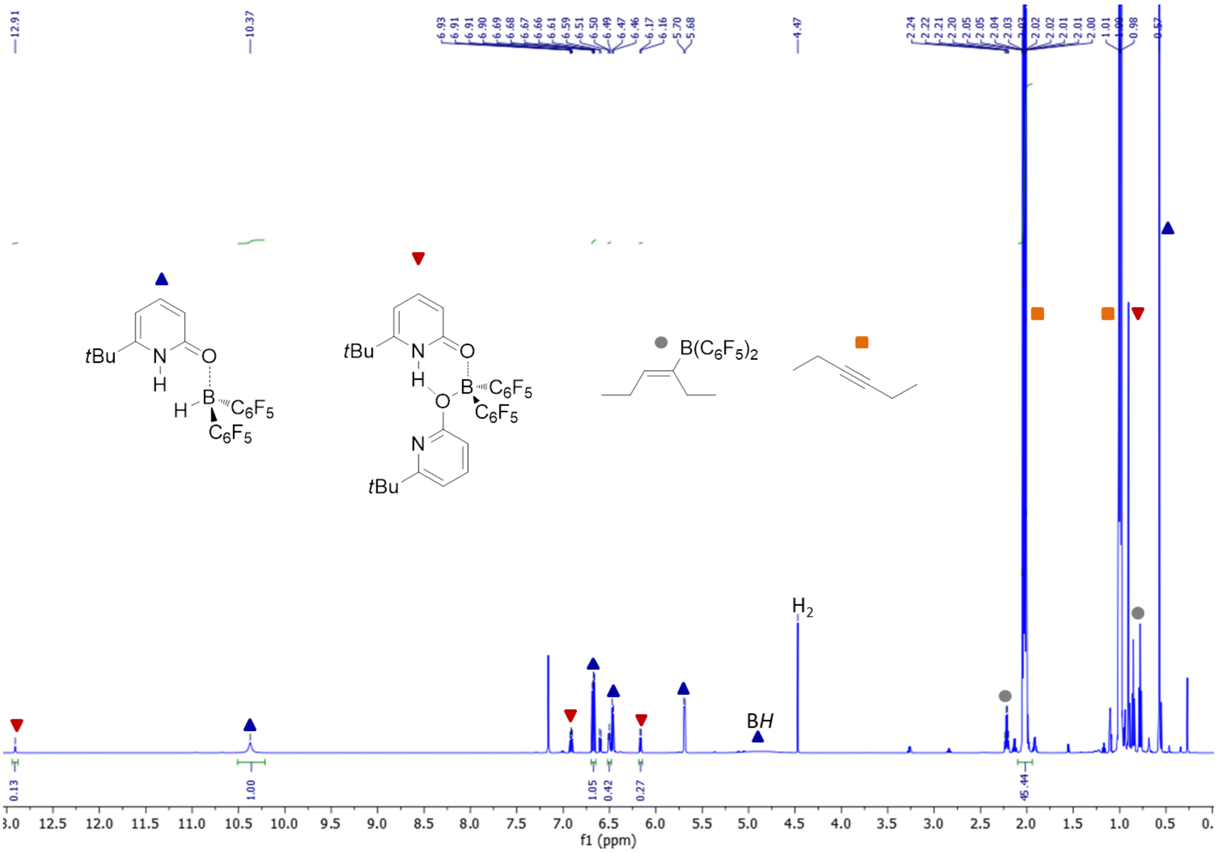

Figure SI 13: ^1H NMR before the start of the reaction monitoring of the hydrogenation of 3-hexyne (600 MHz, benzene-d_6).

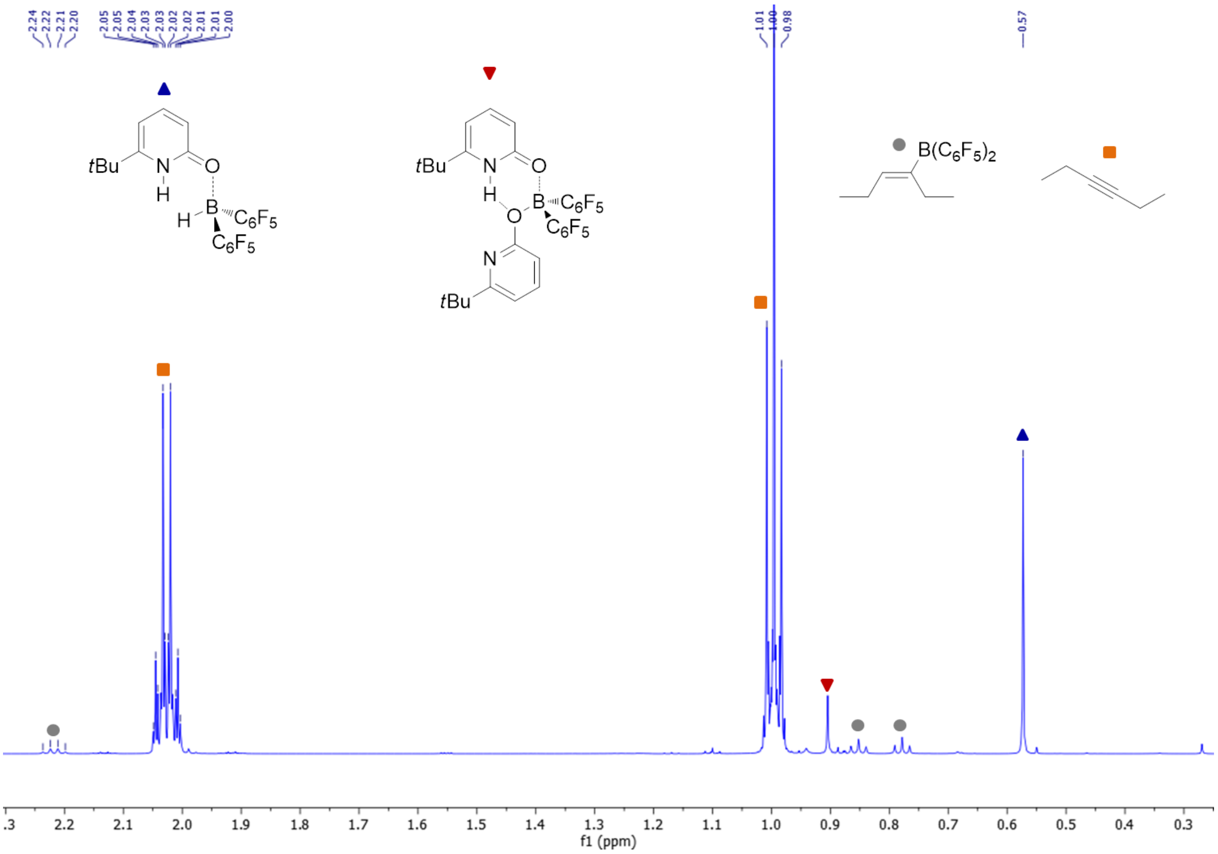

Figure SI 14: High field shifted extract of the ^{1}H NMR before the start of the reaction monitoring of the hydrogenation of 3-hexyne (600 MHz, benzene-d_6).

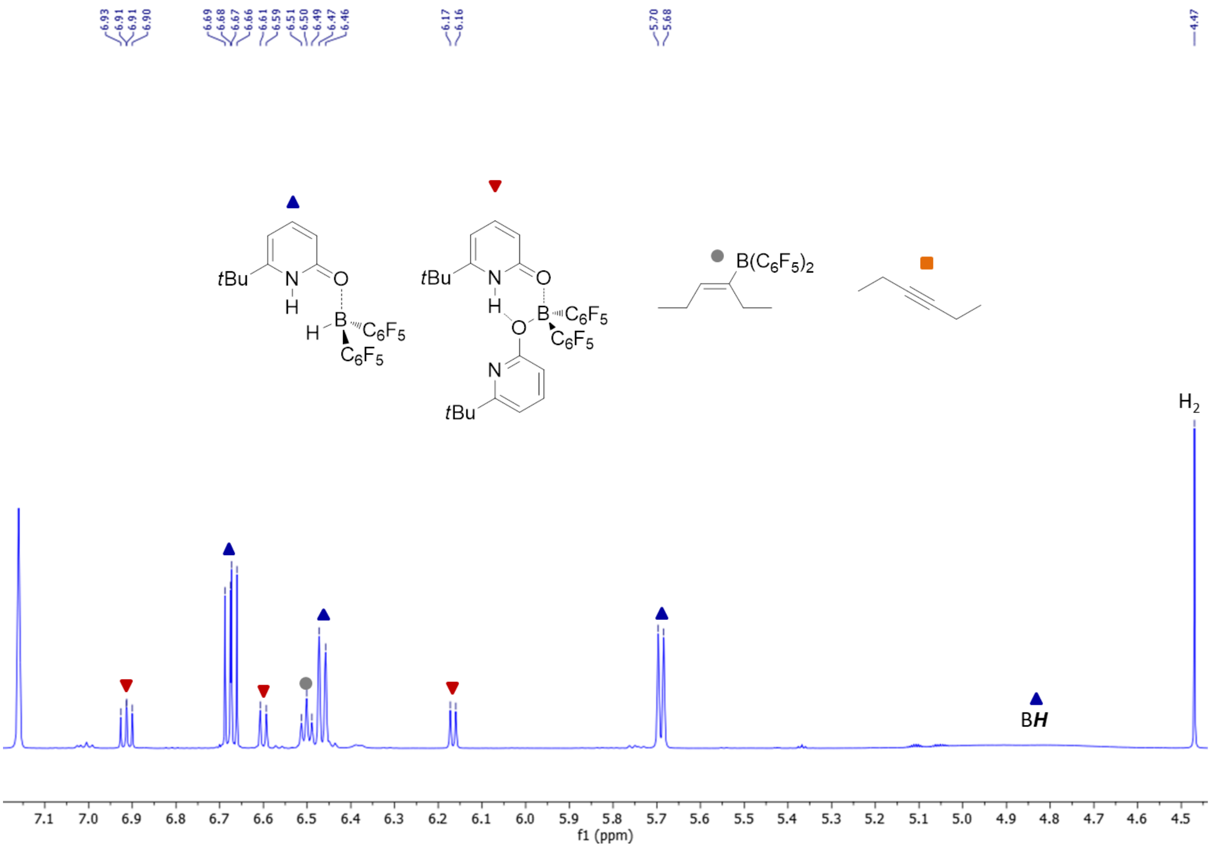

Figure SI 15: Low field shifted extract of the ^{1}H NMR before the start of the reaction monitoring of the hydrogenation of 3-hexyne (600 MHz, benzene-d_6).

In the ^{11}B NMR spectrum, two high field shifted signals (5.0 ppm and -7.2 ppm) are visible which can be assigned to the tetra-substituted pyridone borane **4** and bispyridone complex **8.** In addition, one low field shifted signal (63.1 ppm) is visible which can be assigned to the tri-substituted boron species, the hydroboration product of 3-hexyne with Piers borane (Figure SI 16).

The assignments are supported by the literature known spectra for pyridone borane **4** and bispyridone complex **8** and the spectra of the independent synthesis of the hydroboration product of 3-hexyne with Piers borane (see chapter 3.5).[7]

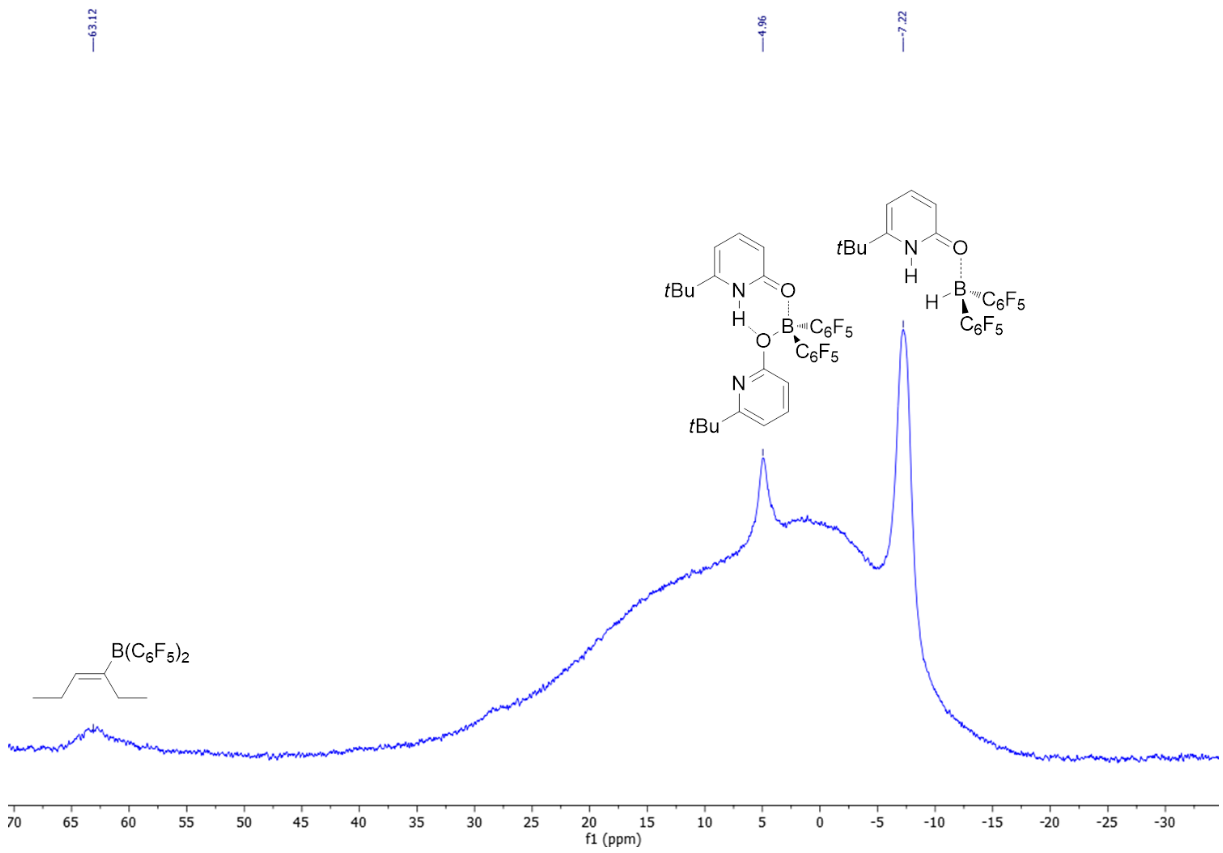

Figure SI 16: ^{11}B NMR before the start of the reaction monitoring of the hydrogenation of 3-hexyne (193 MHz, benzene-d_6).

Afterward, the sample was heated to 60 °C inside the NMR spectrometer and the reaction was monitored by ^{1}H NMR spectroscopy overnight.

Figure SI 17 and Figure SI 18 show high field and low field excerpts of the ^{1}H NMR spectra after 0, 4, 8, 12 and 15 h. As expected, the signals broaden and show slight variation in their chemical shift which is attributed to the elevated temperature. The signals for the different species were assigned by comparing them to room temperature spectra. Over time signals for the hydrogenation product (*Z*)-hex-2-ene (orange square) increased, while the signals for H_2 and 3-hexyne (blue up-pointing triangle) decreased in intensity. The signals for bispyridone complex **8** (red down-pointing triangle) and the hydroboration product of 3-hexyne with Piers borane did not significantly change in intensity indicating that these two species are the resting state of the catalytic cycle. The signals for pyridone borane **4** (black cross) decreased in intensity and new signals which can be assigned to boroxypyridine **3** (blue up-pointing triangle) appeared with progressing reaction time and hydrogen consumption.

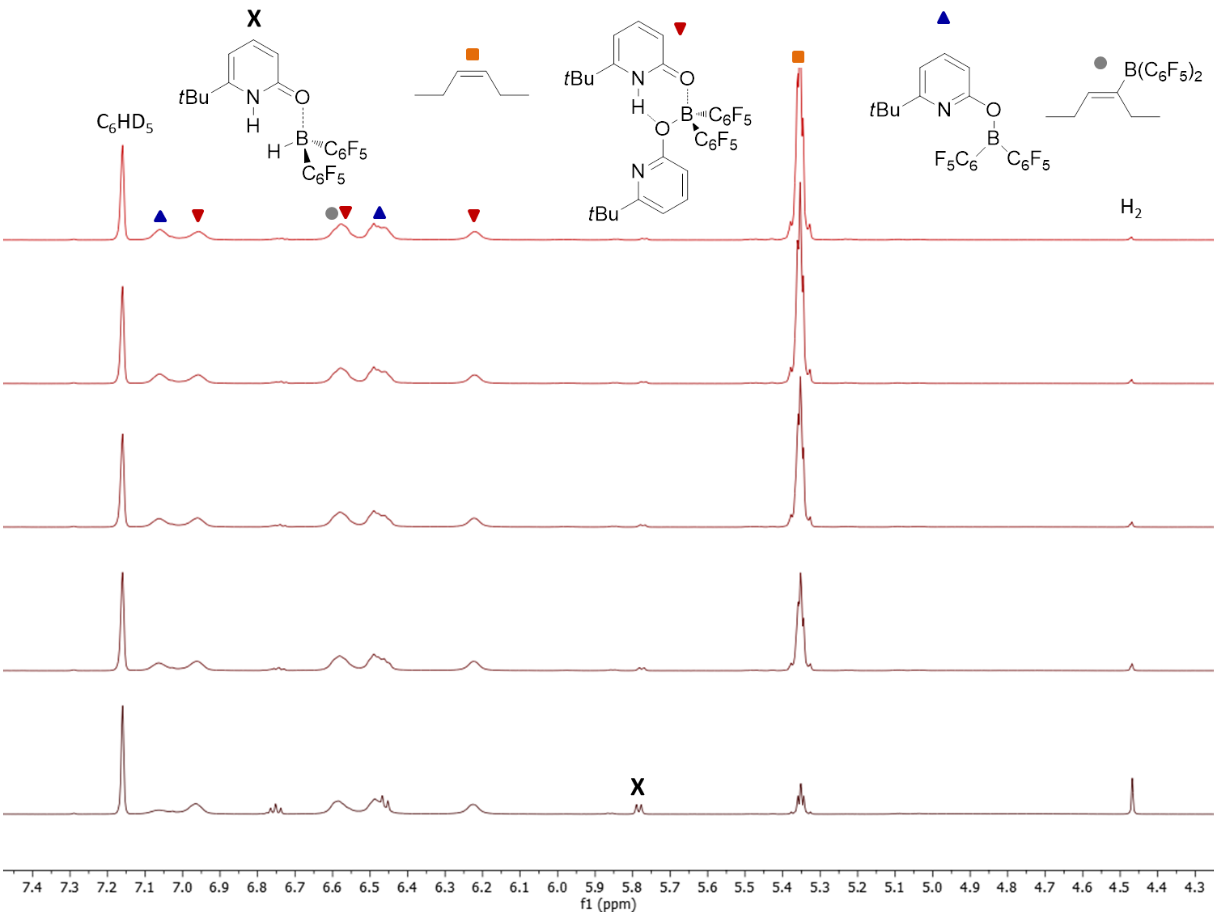

Figure SI 17: Low field excerpts of selected ^{1}H NMR spectra of reaction monitoring of the hydrogenation of 3-hexyne after 0, 4, 8, 12 and 15 h (600 MHz, benzene-d_6).

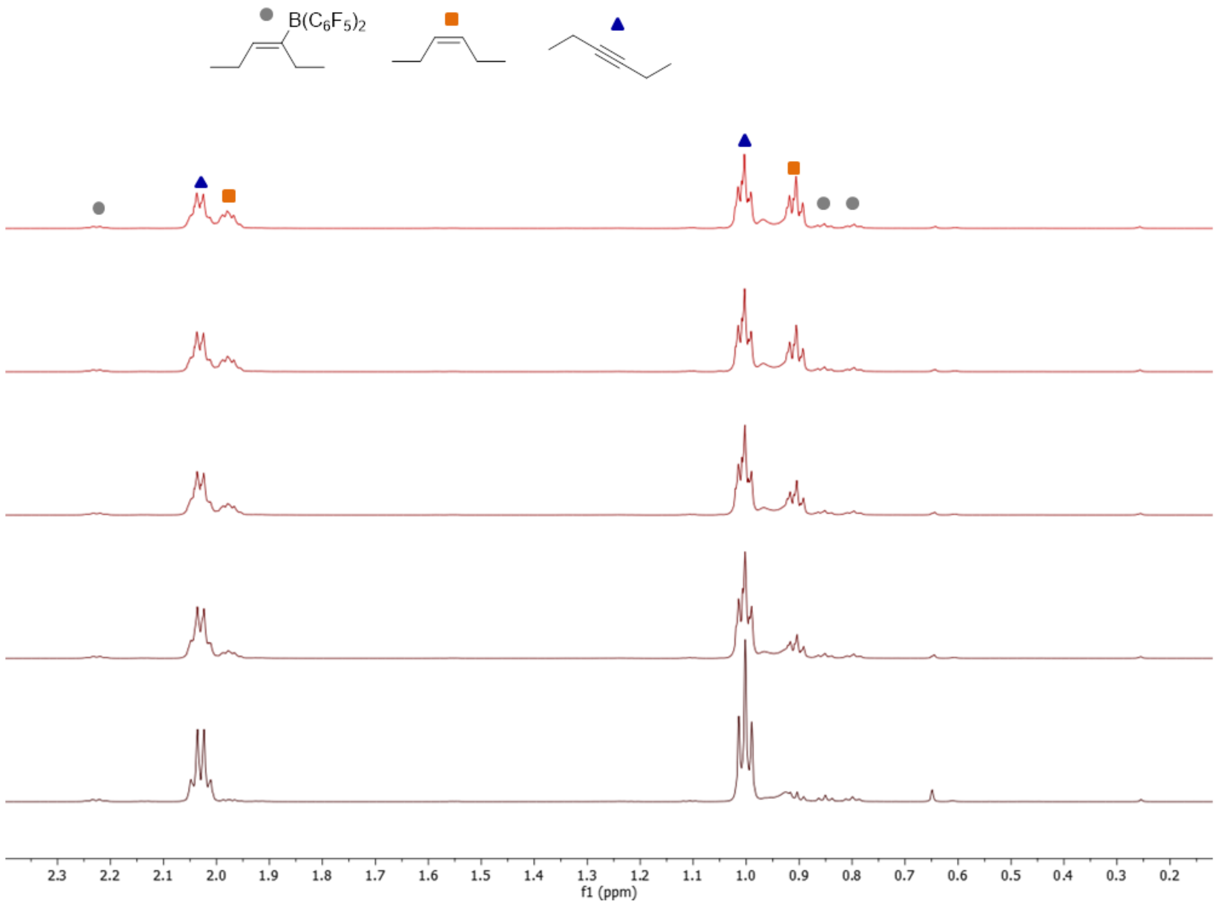

Figure SI 18: High field excerpts of **s**elected ^{1}H NMR spectra of reaction monitoring of the hydrogenation of 3-hexyne after 0, 4, 8, 12 and 15 h (600 MHz, benzene-d_6).

Afterwards, the NMR tube was cooled to room temperature and ^{1}H and ^{11}B NMR spectra were recorded (Figure SI 19, Figure SI 20, Figure SI 21 and, Figure SI 22). These spectra show that the signals for pyridone borane **4** disappeared while signals for boroxypyridine **3** (blue up-pointing triangle) appeared. The low field shifted region also shows signals for bispyridone complex **8** (red, down-pointing triangle) and hydroborated 3-hexyne (grey dot), caused by a slight excess of Piers borane **6** in the reaction mixture. The high field excerpt shows that 3-hexyne (blue up-pointing triangle), its hydroborated form (grey dot), and (*Z*)-hex-2-ene (orange square) were present in a 1.42 to 1 ratio. This corresponds to a yield of about 41%.

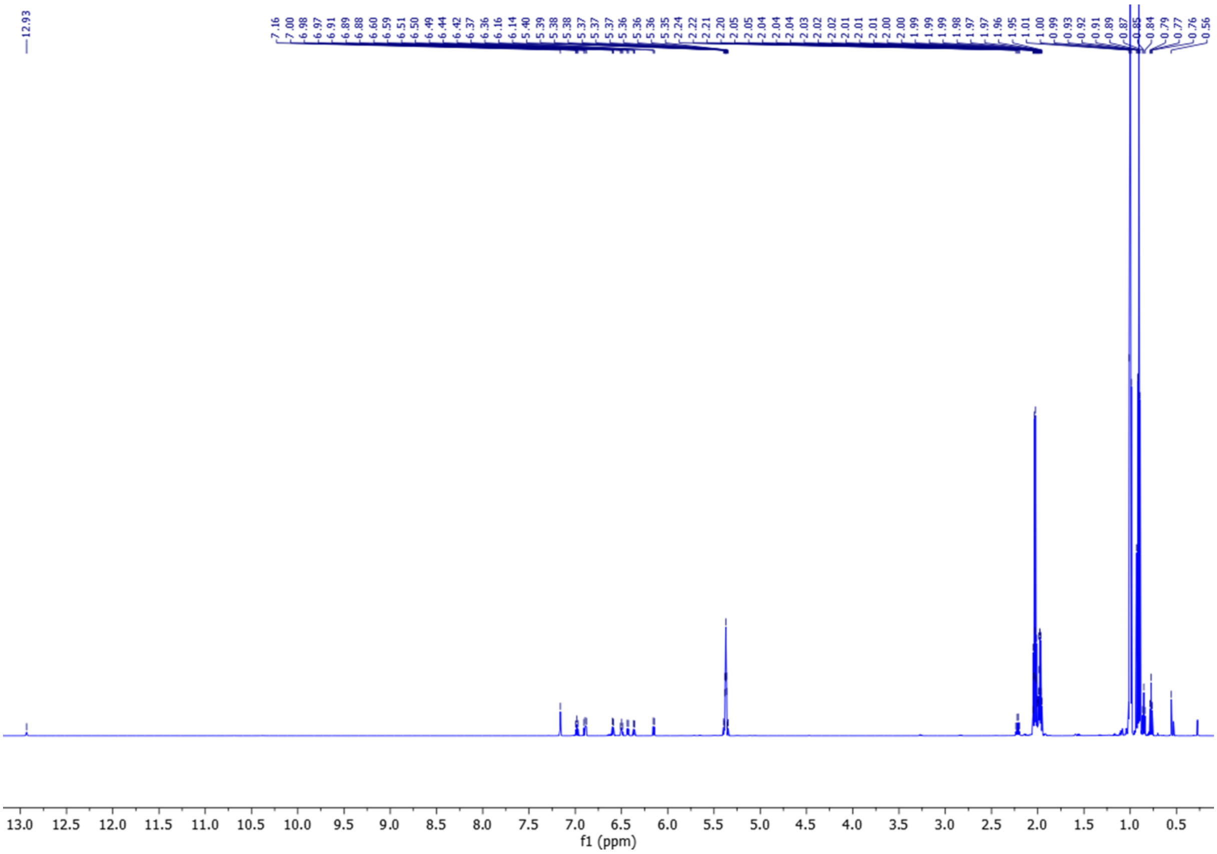

Figure SI 19: Room temperature ^{1}H NMR spectrum after the reaction monitoring at 60 °C overnight of the hydrogenation of 3-hexyne (600 MHz, benzene-d_6).

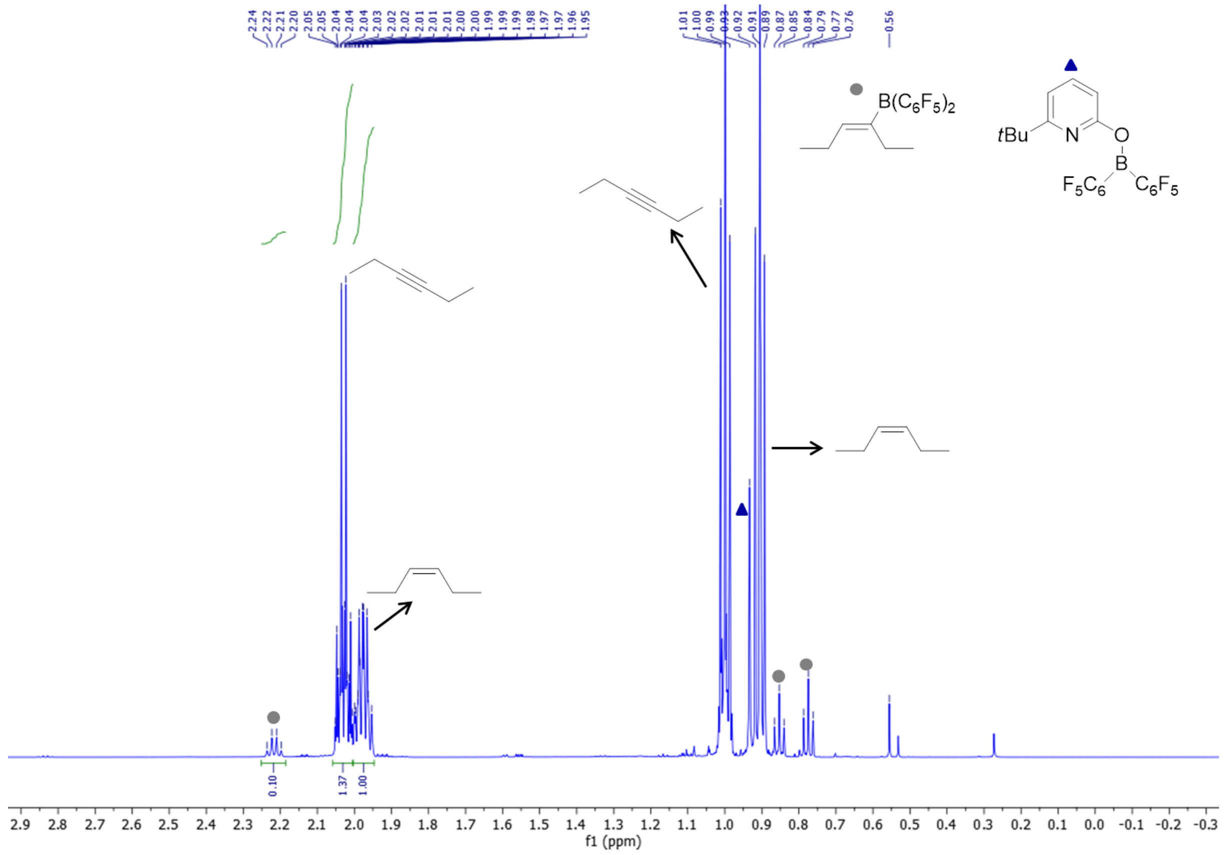

Figure SI 20: High field excerpt of the room temperature ^{1}H NMR spectrum after the reaction monitoring at 60 °C of the hydrogenation of 3-hexyne (600 MHz, benzene-d_6).

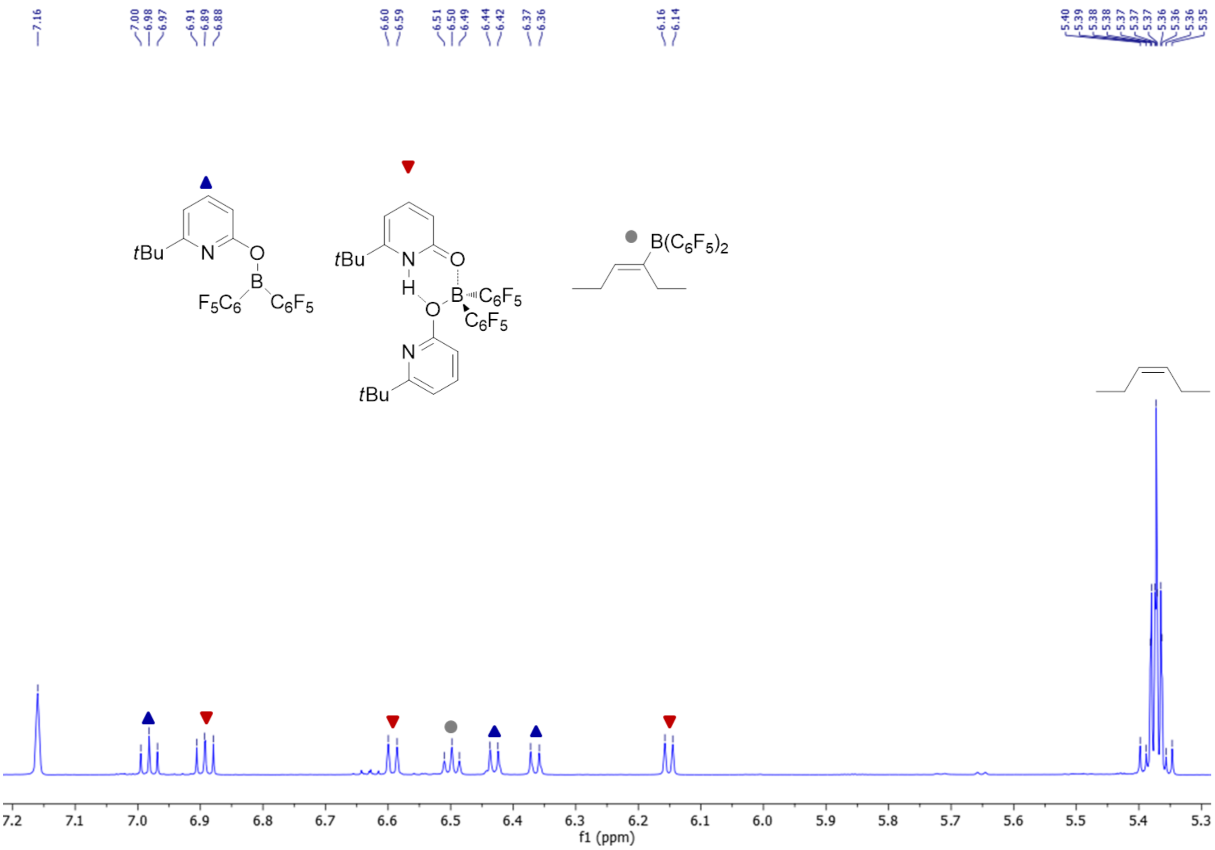

Figure SI 21: Low field excerpt of the room temperature ¹H NMR spectrum after the reaction monitoring at 60 °C of the hydrogenation of 3-hexyne (600 MHz, benzene-d_6).

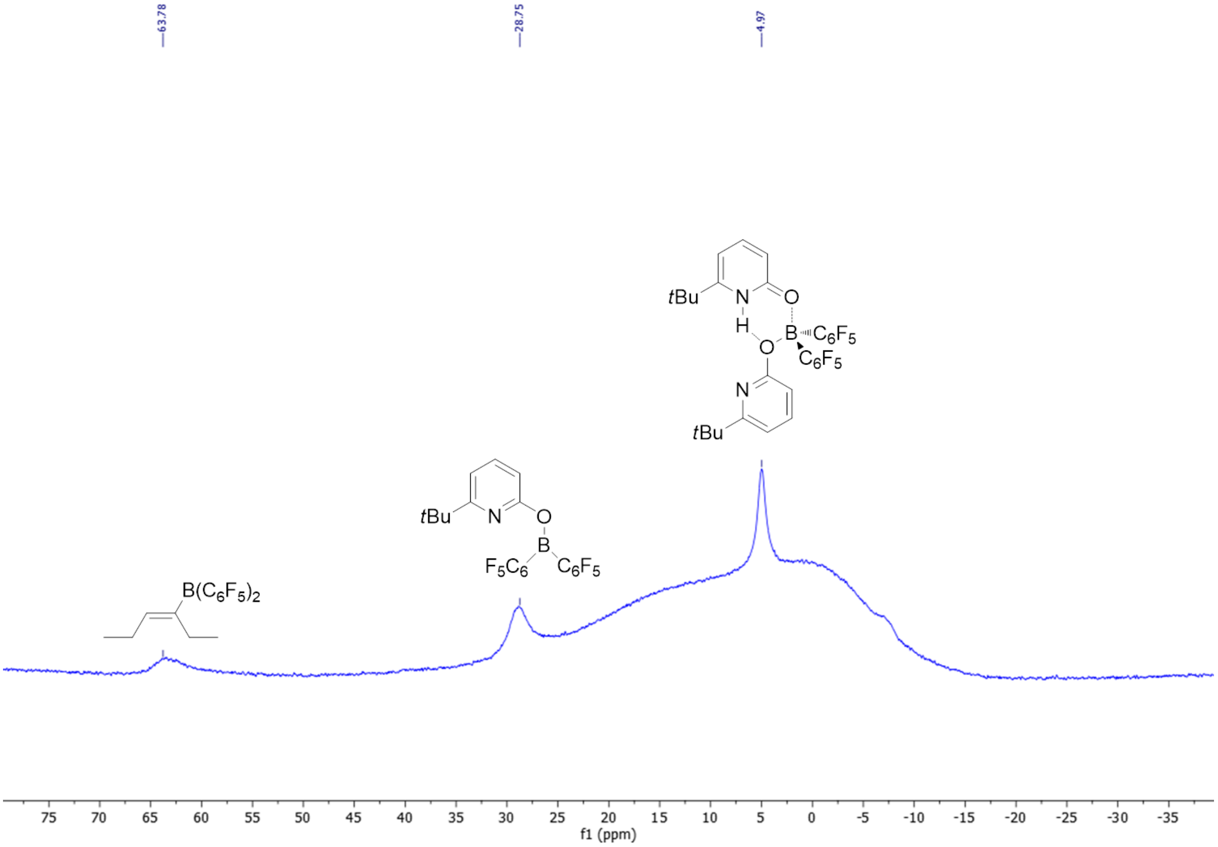

Figure SI 22: ^{11}B NMR spectrum at room temperature after the reaction monitoring at 60 °C of the hydrogenation of 3-hexyne (193 MHz, benzene-d_6).

3.4 Reaction of (*Z*)-hex-3-en-3-ylbis(perfluorophenyl)borane with pyridone 5

Piers borane **6** (17.3 mg, 0.05 mmol) and 3-hexyne (5.7 µL, 0.05 mmol) were suspended in benzene-d_6 (0.4 ml) and the suspension was shaken until a clear solution was obtained. The solution was transferred to an NMR tube with J Young valve and rinsed two times with benzene-d_6 (0.1 ml). The product (*Z*)-hex-3-en-3-ylbis(perfluorophenyl)borane was completely characterized (see chapter 3.5). Subsequently, pyridone **5** (7.5 mg, 0.05 mmol) was added and ^{1}H, COSY, and ^{11}B NMR spectra were measured directly afterwards (Figure SI 23, Figure SI 24, Figure SI 25, Figure SI 26, Figure SI 27, Figure SI 28, and Figure SI 29).

These spectra show three main components: the bispyridone complex **8**, (*Z*)-hex-3-ene and a set of signals which were assigned to the (*Z*)-hex-3-en-3-ylbis(perfluorophenyl)borane pyridone complex **10**. In the high field shifted region two triplets at 0.70 and 1.06 ppm are visible corresponding to the CH_3 groups of the hexene moiety. The triplet at 0.70 ppm shows a COSY coupling to a quadruplet at 2.50 ppm and the triplet at 1.06 ppm to a pentet at 2.17 ppm. Both were assigned to the CH_2 groups of the hexene moiety. The pentet shows a COSY signal to triplet at 5.73 ppm which was assigned to the olefinic hydrogen of the hexene moiety. Additionally, the low field shifted region shows three typical pyridone signals at 5.68, 6.14 and 6.63 ppm, which show cross-coupling signals in the COSY spectrum and a low shifted broad signal at 10.97 ppm which was assigned to the NH group of the pyridone moiety. The singlet signal at 0.66 ppm and an integral of nine was attributed to the *tert*-butyl group of the pyridone. In the ^{11}B NMR spectrum two signals were visible. At 4.97 ppm for the bispyridone complex **8** and at 1.68 ppm for the alkenyl borane pyridone complex **10** (Figure SI 29). Because complex **10** reacts over the course of a few hours at room temperature undergoing the protodeborylation reaction (*vide infra*) it was only possible to characterize it by ^{1}H and ^{11}B NMR spectra (for a full characterization see chapter 3.6).

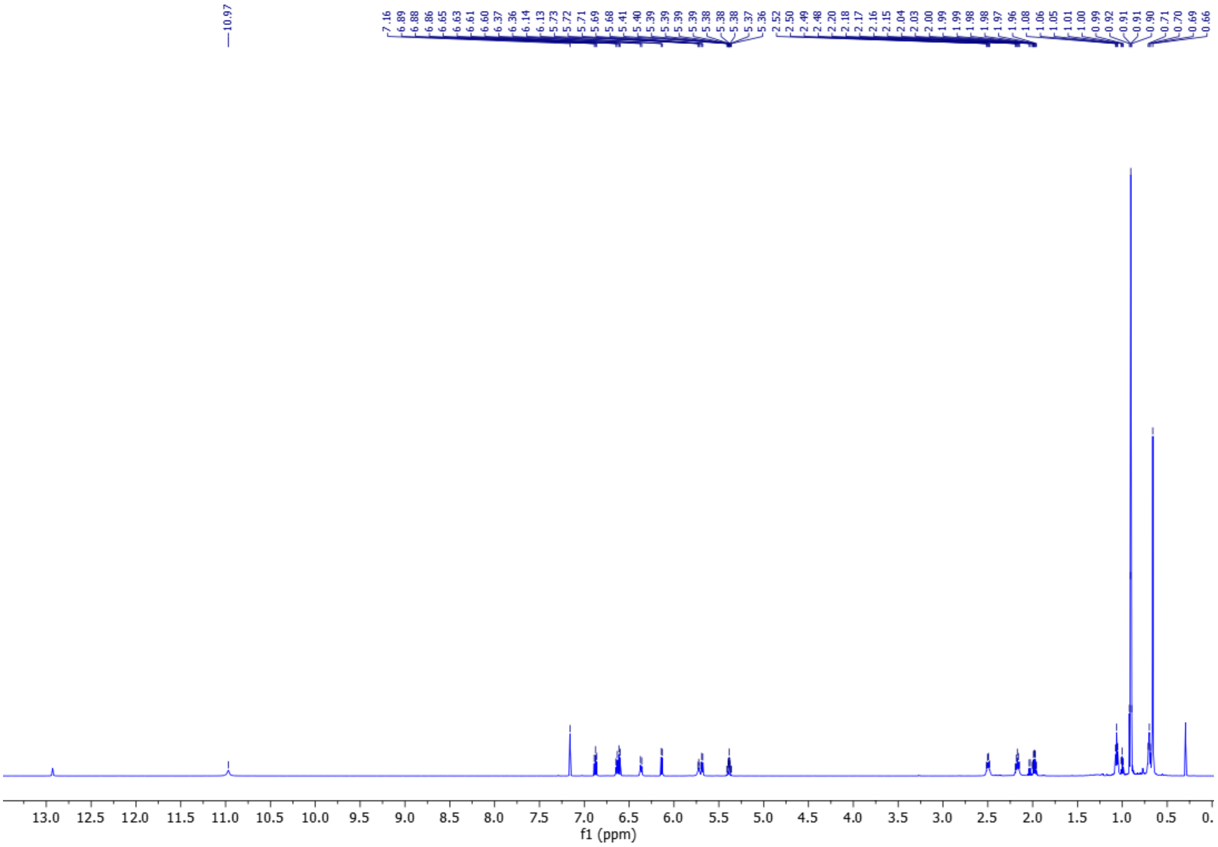

Figure SI 23: ^{1}H NMR spectrum directly after the addition of pyridone **5** to the hydroboration product of 3-hexyne and Piers borane (600 MHz, benzene-d_6).

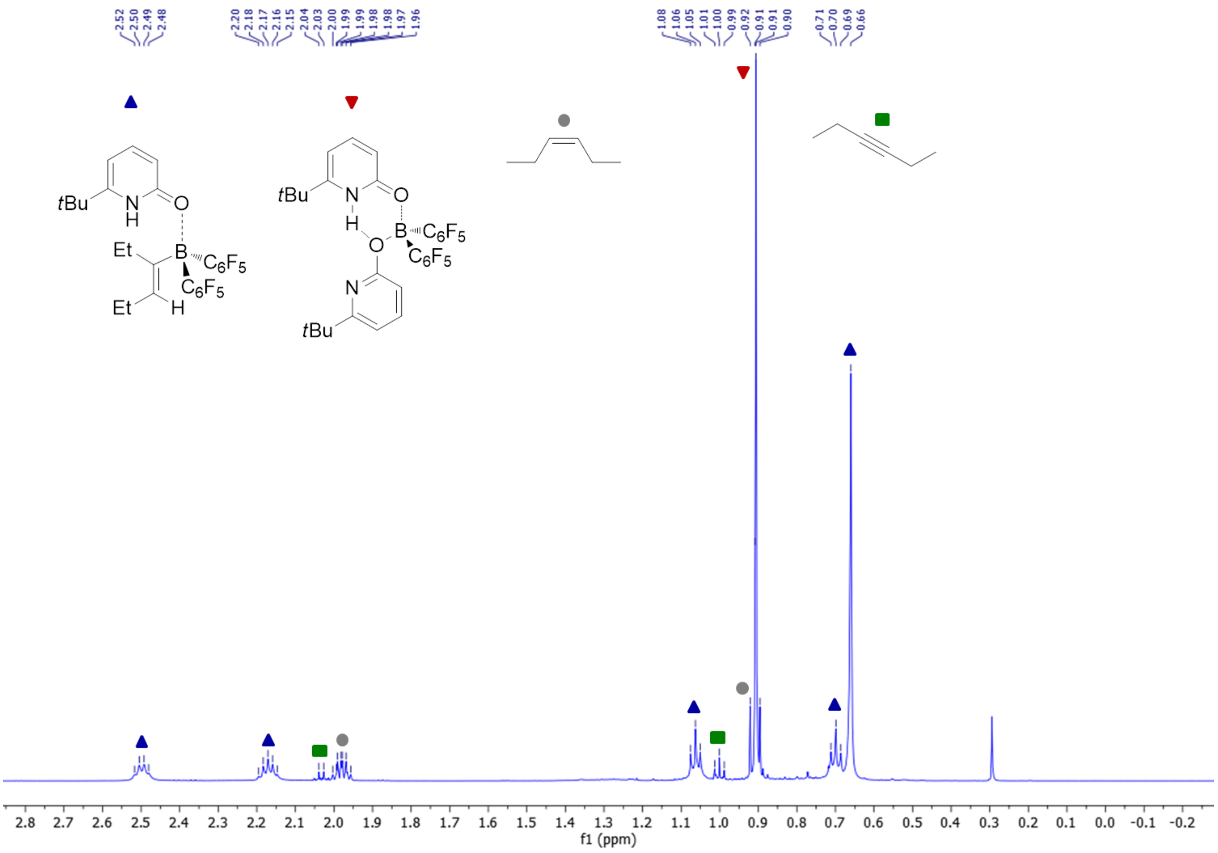

Figure SI 24: High field excerpt of the ^{1}H NMR spectrum directly after the addition of pyridone **5** to the hydroboration product of 3-hexyne and Piers borane (600 MHz, benzene-d_6).

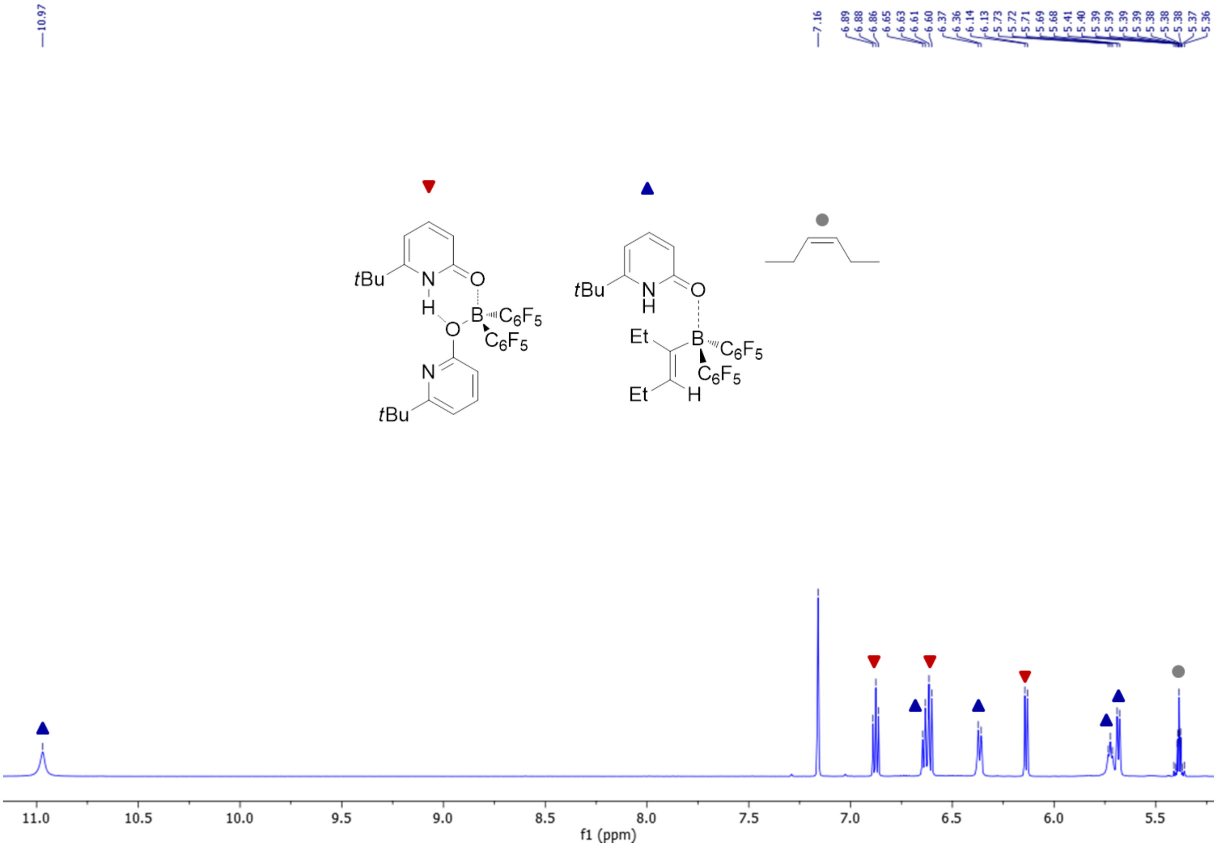

Figure SI 25: Low field excerpt of the ^{1}H NMR spectrum directly after the addition of pyridone **5** to the hydroboration product of 3-hexyne and Piers borane (600 MHz, benzene-d_6).

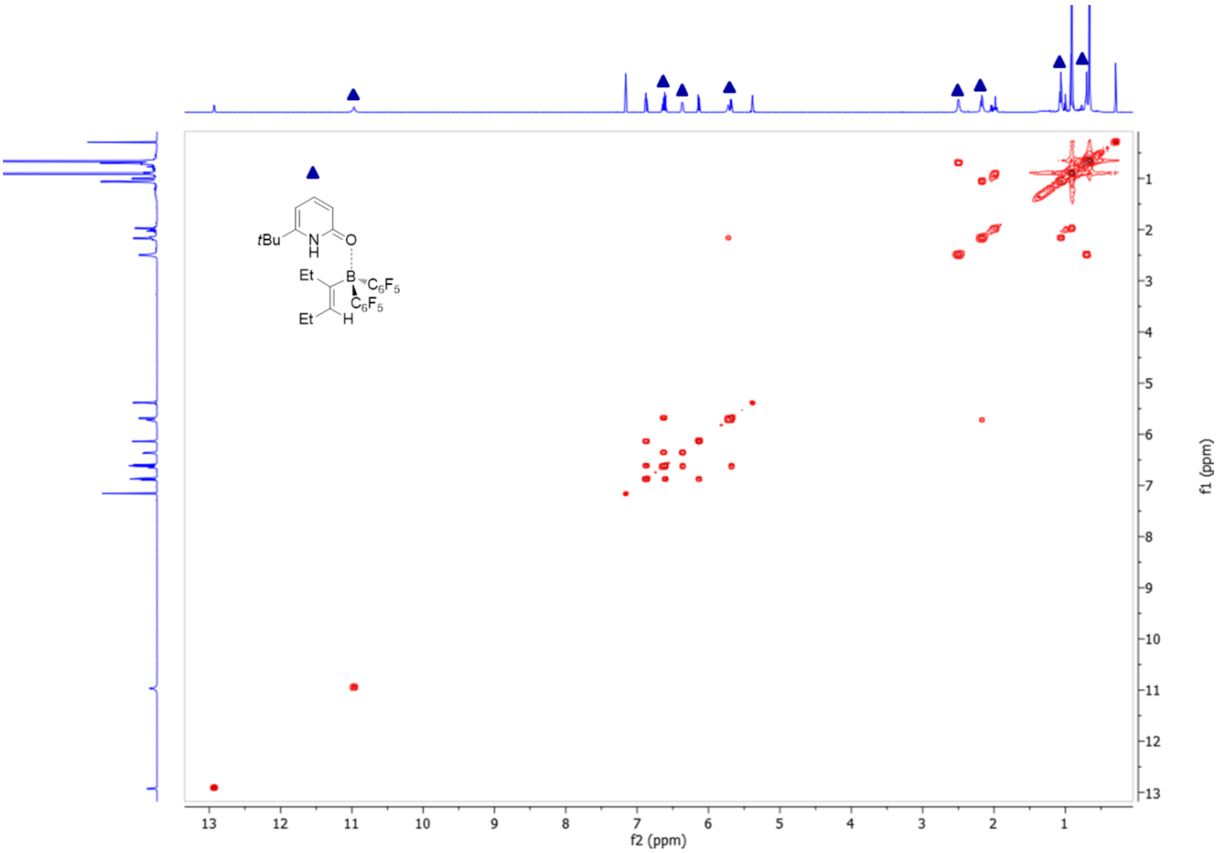

Figure SI 26: COSY NMR spectrum directly after addition of pyridone **5** to the hydroboration product of 3-hexyne and Piers borane (600 MHz, benzene-d_6).

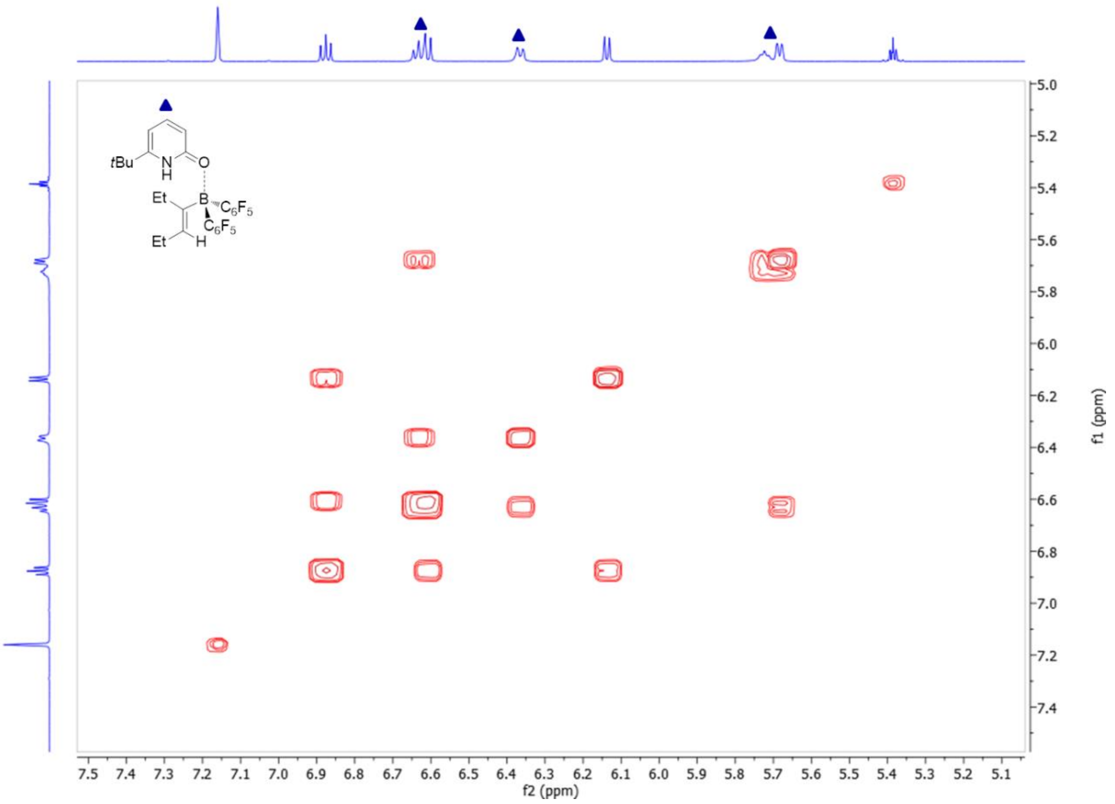

Figure SI 27: Low field excerpt of the COSY NMR spectrum directly after the addition of pyridone **5** to the hydroboration product of 3-hexyne and Piers borane (600 MHz, benzene-d_6).

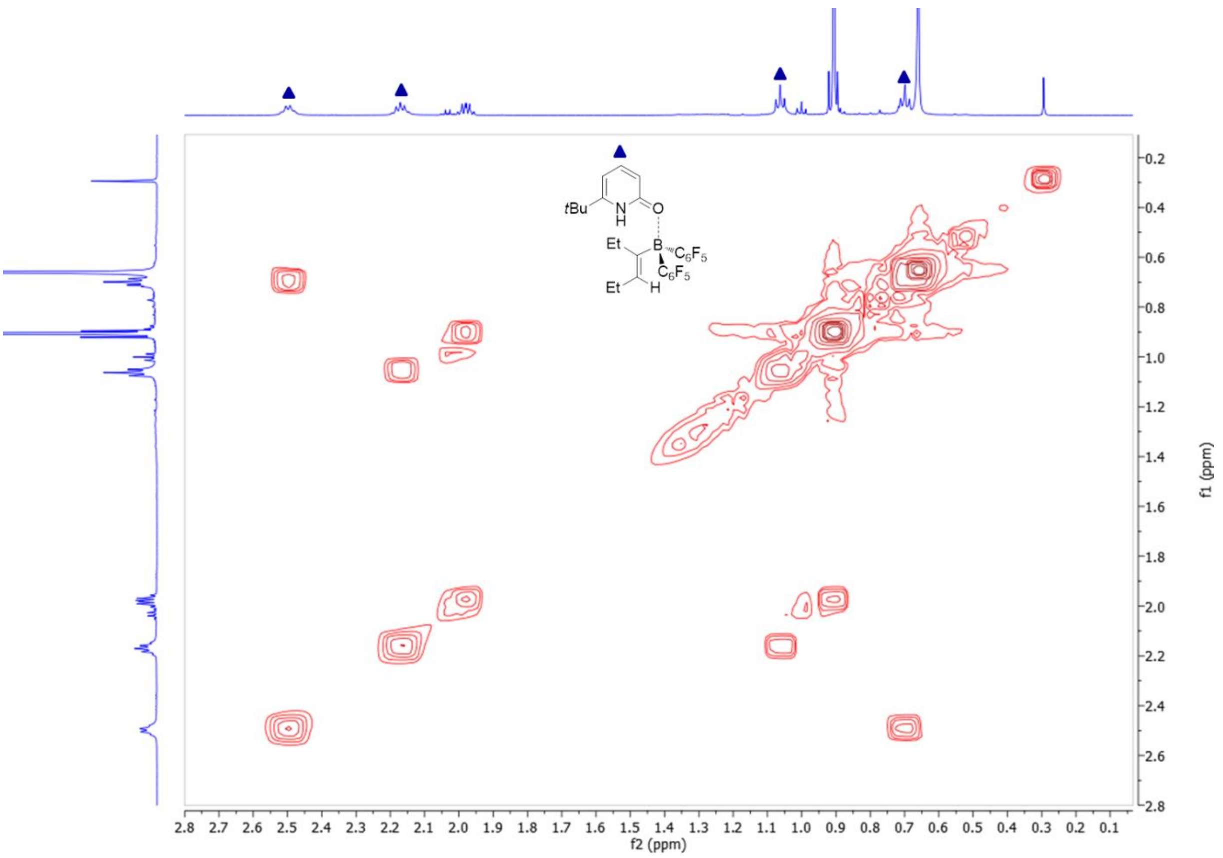

Figure SI 28: High field excerpt of the COSY NMR spectrum directly after the addition of pyridone **5** to the hydroboration product of 3-hexyne and Piers borane (600 MHz, benzene-d_6).

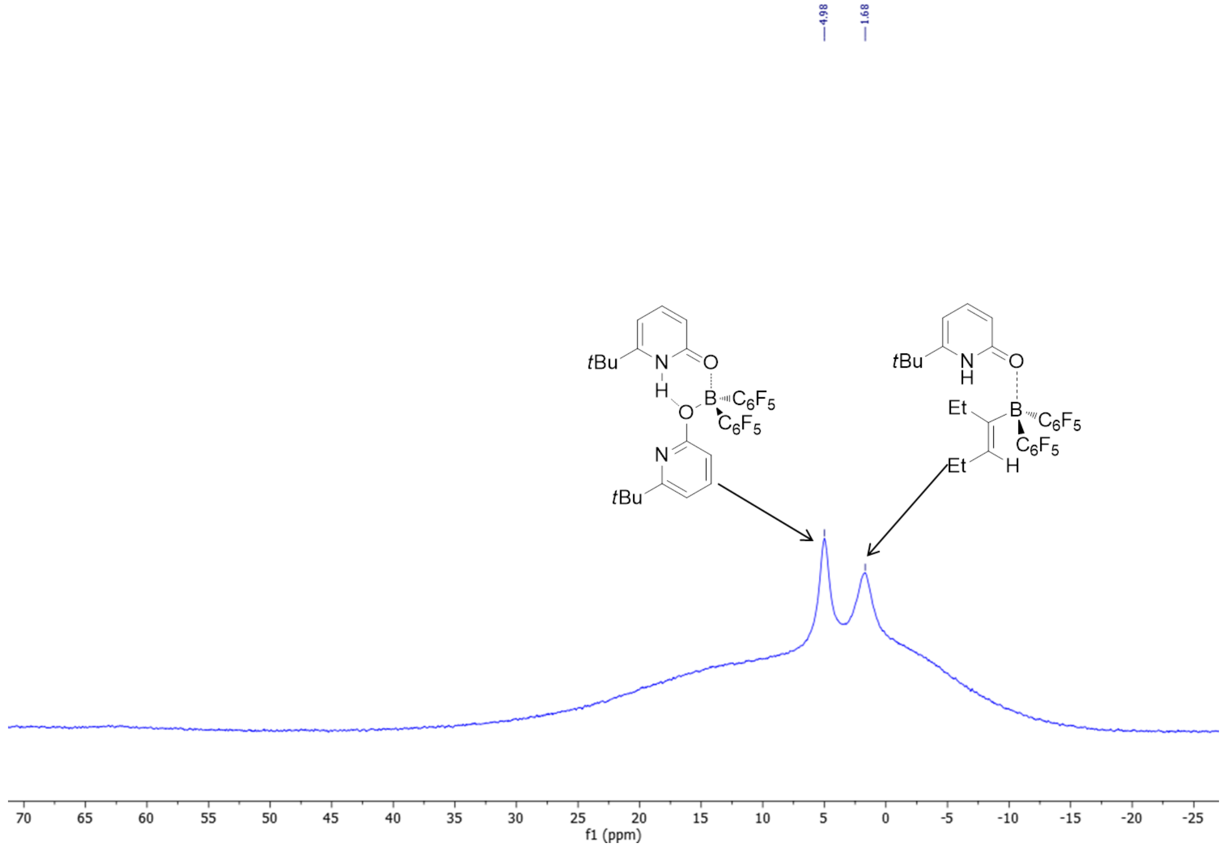

Figure SI 29: ^{11}B NMR spectrum directly after the addition of pyridone **5** to the hydroboration product of 3-hexyne and Piers borane (193 MHz, benzene-d_6).

The formation of (*Z*)-hex-3-ene is attributed to the protodeborylation reaction of complex **10** as described in the proposed catalytic cycle. NMR experiments and computations indicate that bispyridone complex **8** and (*Z*)-hex-3-en-3-ylbis(perfluorophenyl)borane is the resting state of the catalytic cycle. Complex **8** itself is not able to undergo the protodeborylation reaction. An EXSY experiment showed exchange signals between signals corresponding to **8** and **10**, a clear indication that bispyridone complex **8** can be converted to **10**. The later one is able to undergo the protodeborylation reaction. This yields the hydrogenation product (*Z*)-hex-3-ene and boroxypyridine **3**. Additionally, the EXSY experiment showed that **10** and (*Z*)-hex-3-en-3-ylbis(perfluorophenyl)borane are in equilibrium and that the dissociation of the borane species from the pyridone occurs at room temperature (Figure SI 30, Figure SI 31, Figure SI 32, and Figure SI 33).[5]

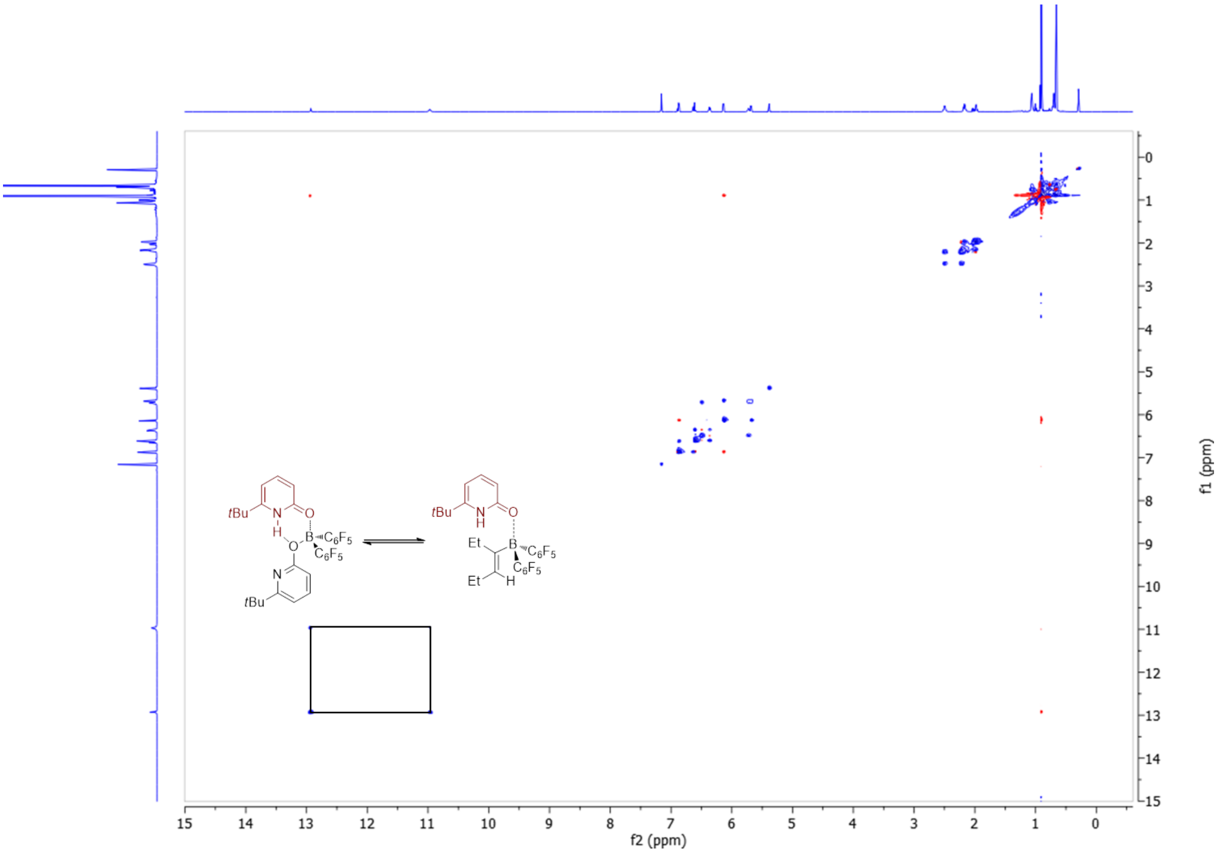

Figure SI 30: EXSY NMR spectrum directly after the addition of pyridone **5** to the hydroboration product of 3-hexyne and Piers borane (600 MHz, benzene-d_6).

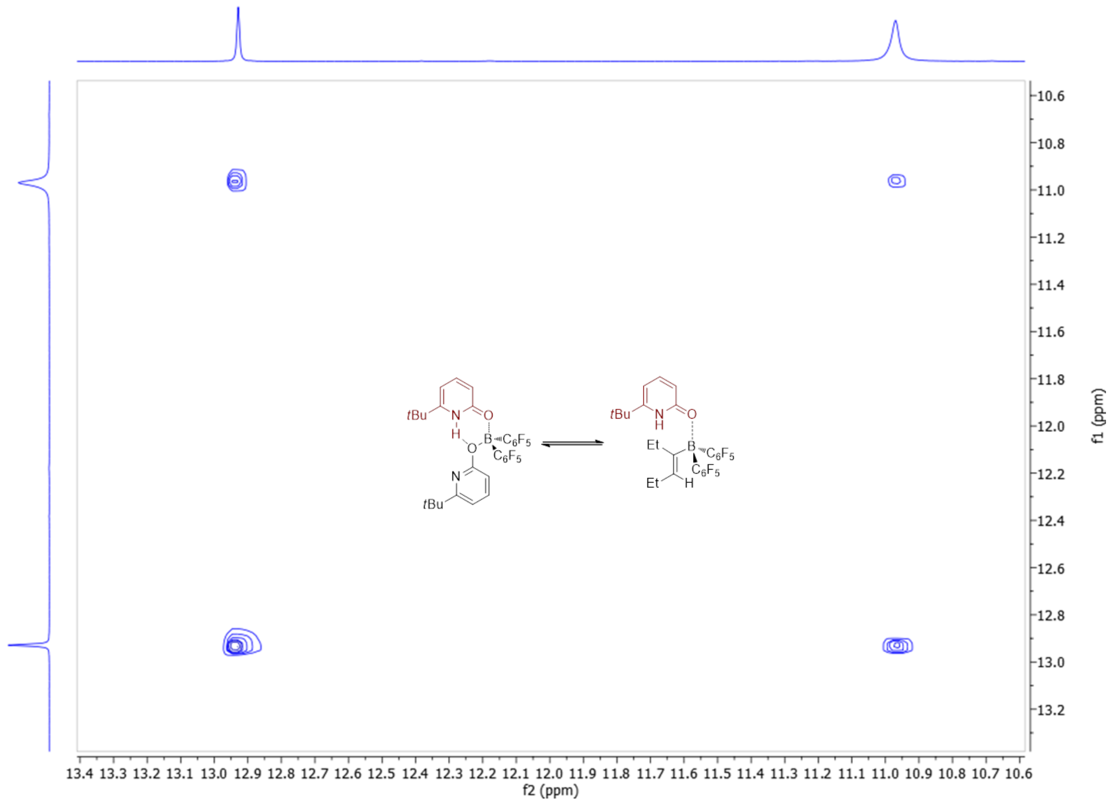

Figure SI 31: Low field excerpt of the EXSY NMR spectrum directly after the addition of pyridone **5** to the hydroboration product of 3-hexyne and Piers borane (600 MHz, benzene-d_6).

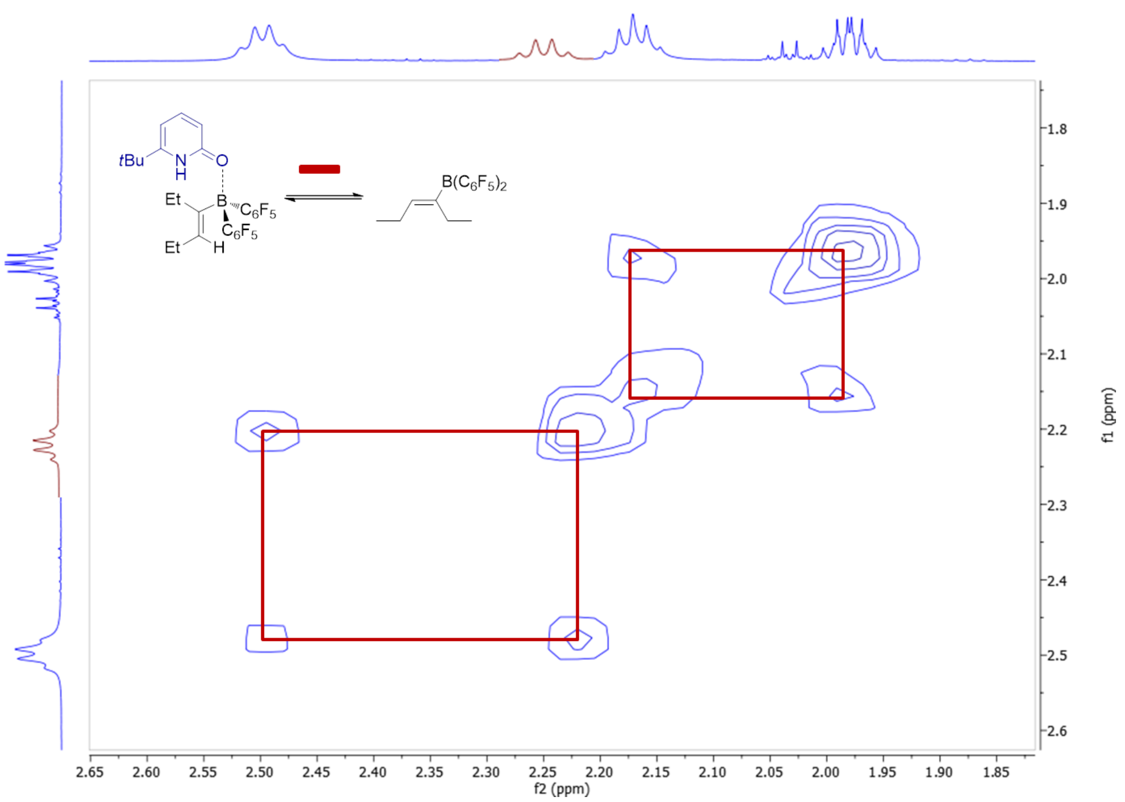

Figure SI 32: High field excerpt of the EXSY directly after the addition of pyridone **5** to the hydroboration product of 3-hexyne and Piers borane (600 MHz, benzene-d_6). The red marked signal in the 1D spectrum shows the signal which has appeared in the reaction mixture during the NOESY experiment.

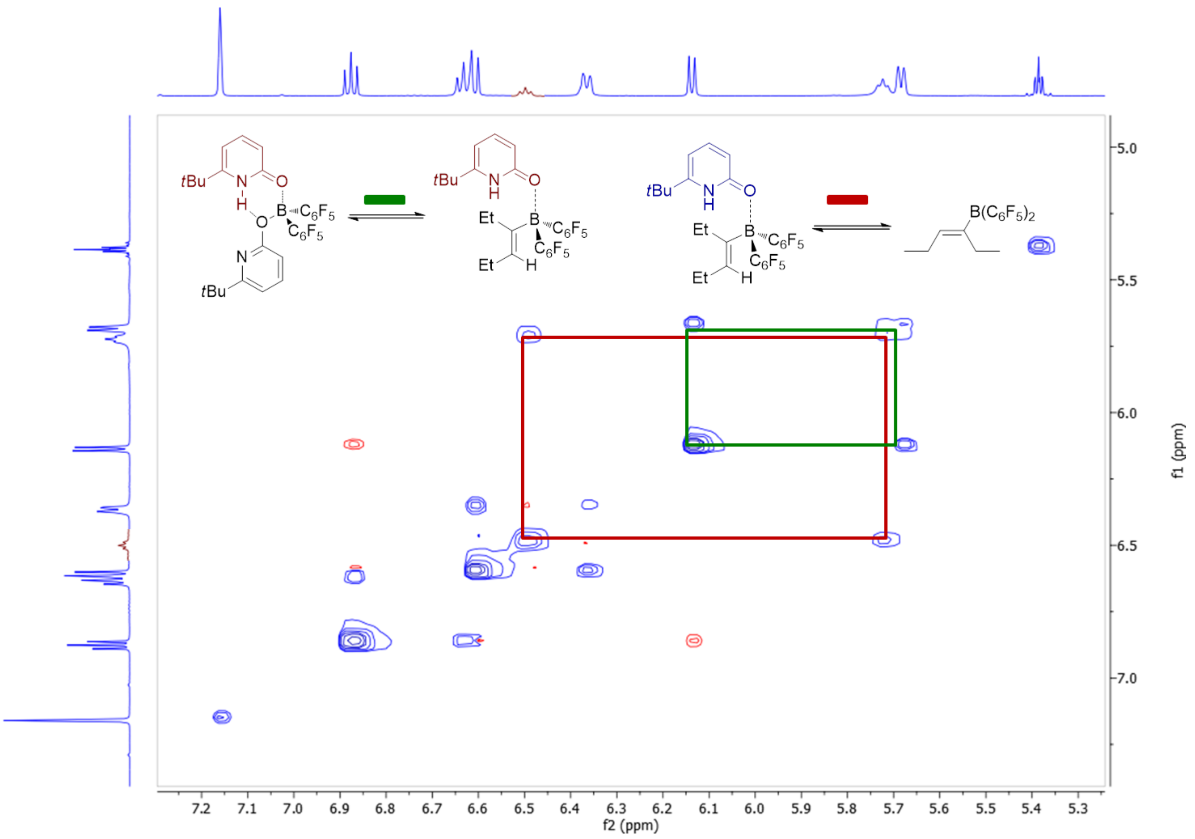

Figure SI 33: Low field excerpt of the EXSY spectrum directly after the addition of pyridone **5** to the hydroboration product of 3-hexyne and Piers borane (600 MHz, benzene-d_6). The red marked signal in the 1D spectrum shows the signal which has appeared in the reaction mixture during measuring of the NOESY spectrum.

After the EXSY experiment, additional ^{1}H and ^{11}B NMR spectra were measured. They revealed that over the time of the EXSY experiment the reaction mixture changed. The signals assigned to (*Z*)-hex-3-ene and bispyridone complex **8** increased in intensity while the signals for **10** nearly completely disappeared. Additionally, signals of small intensity which are assigned to boroxypyridine **3** and (*Z*)-hex-3-en-3-ylbis(perfluorophenyl)borane appeared (Figure SI 34, Figure SI 35, Figure SI 36, and Figure SI 37).

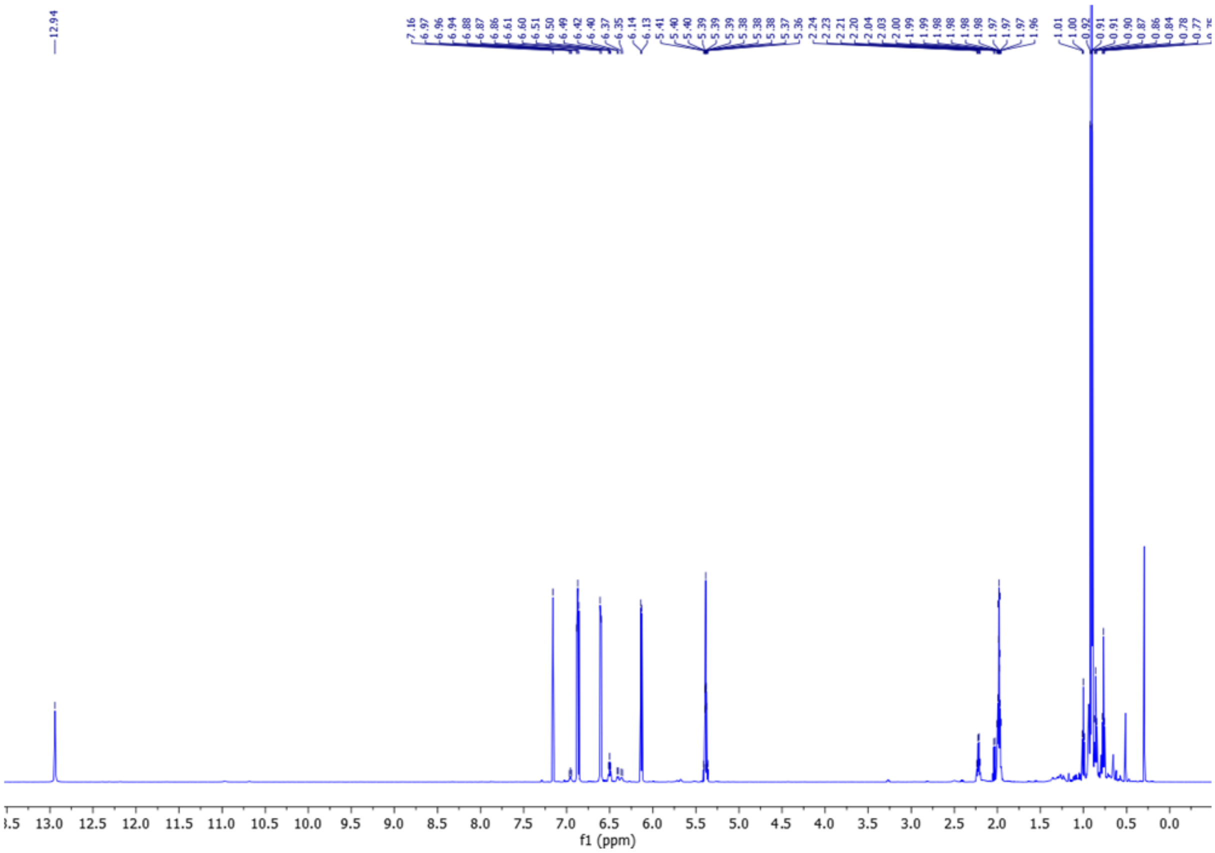

Figure SI 34: ^{1}H NMR spectrum ca. 3 h after the addition of pyridone **5** to the hydroboration product of 3-hexyne and Piers borane (600 MHz, benzene-d_6).

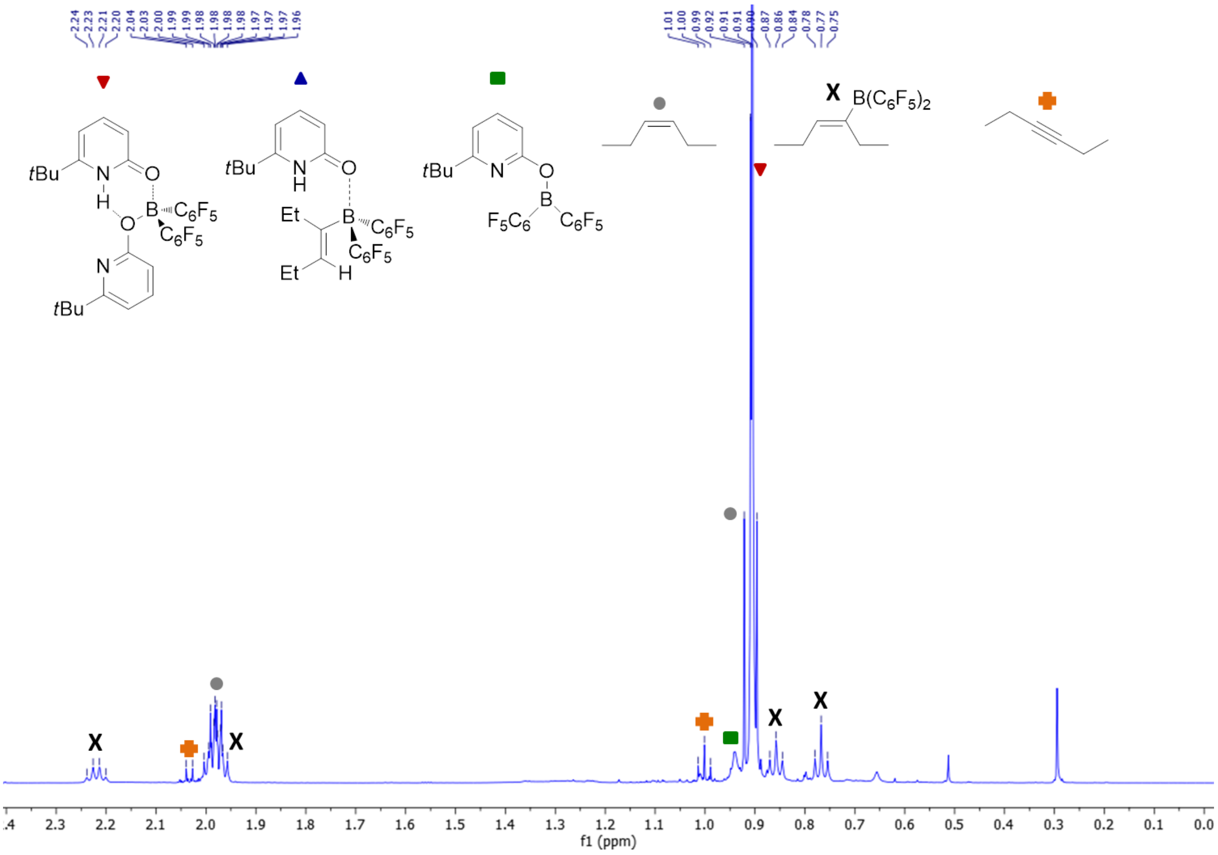

Figure SI 35: High field excerpt of the ^{1}H NMR spectrum ca. 3 h after the addition of pyridone **5** to the hydroboration product of 3-hexyne and Piers borane (600 MHz, benzene-d_6).

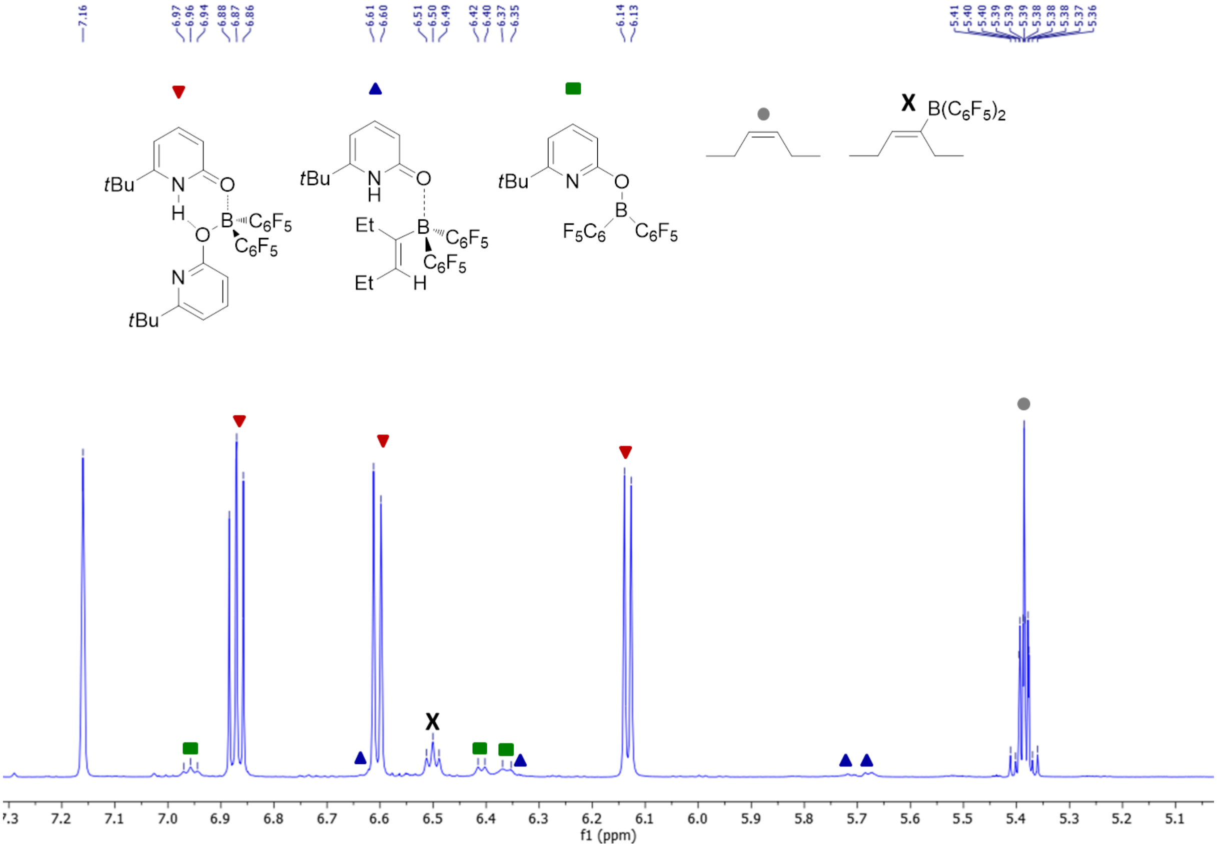

Figure SI 36: Low field excerpt of the ^{1}H NMR spectrum ca. 3 h after the addition of pyridone **5** to the hydroboration product of 3-hexyne and Piers borane (600 MHz, benzene-d_6).

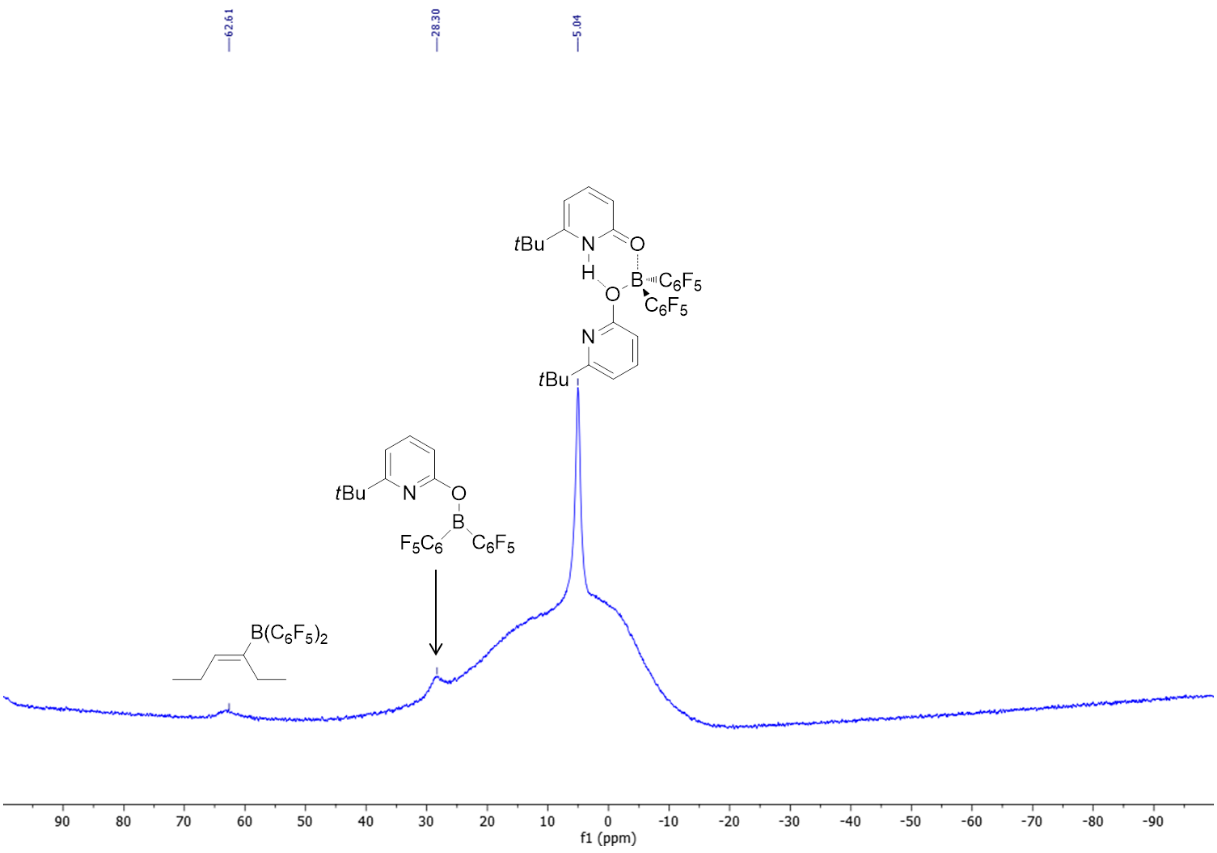

Figure SI 37: ^{11}B NMR spectrum ca. 3 h after the addition of pyridone **5** to the hydroboration product of 3-hexyne and Piers borane (193 MHz, benzene-d_6).

Although trace amounts of **10** were detectable, its signals further decreased in intensity. Ongoing reaction monitoring at room temperature over days showed that the signals for (*Z*)-hex-3-ene and boroxypyridine **3** further increased in intensity (Figure SI 38). Presumably, **10** is formed in low equilibrium concentrations that are not detectable by NMR. The protodeborylation reaction of **10** yields (*Z*)-hex-3-ene and boroxypyridine **3**. Therefore, it is evident that bispyridone complex **8** is not a thermodynamic dead end of the reaction. After 11 d the corresponding ^{11}B NMR spectrum showed signals which were assigned to boroxypyridine **3** and biscomplex **8** (Figure SI 39).

Please note, that the integrals for the detectable species in the ^{1}H NMR are in a ratio of 1.00 to 0.76 to 0.60 regarding pyridone, borane, and hexyne derivatives, presumably due to a fast exchange process of complex **8** and **10**.

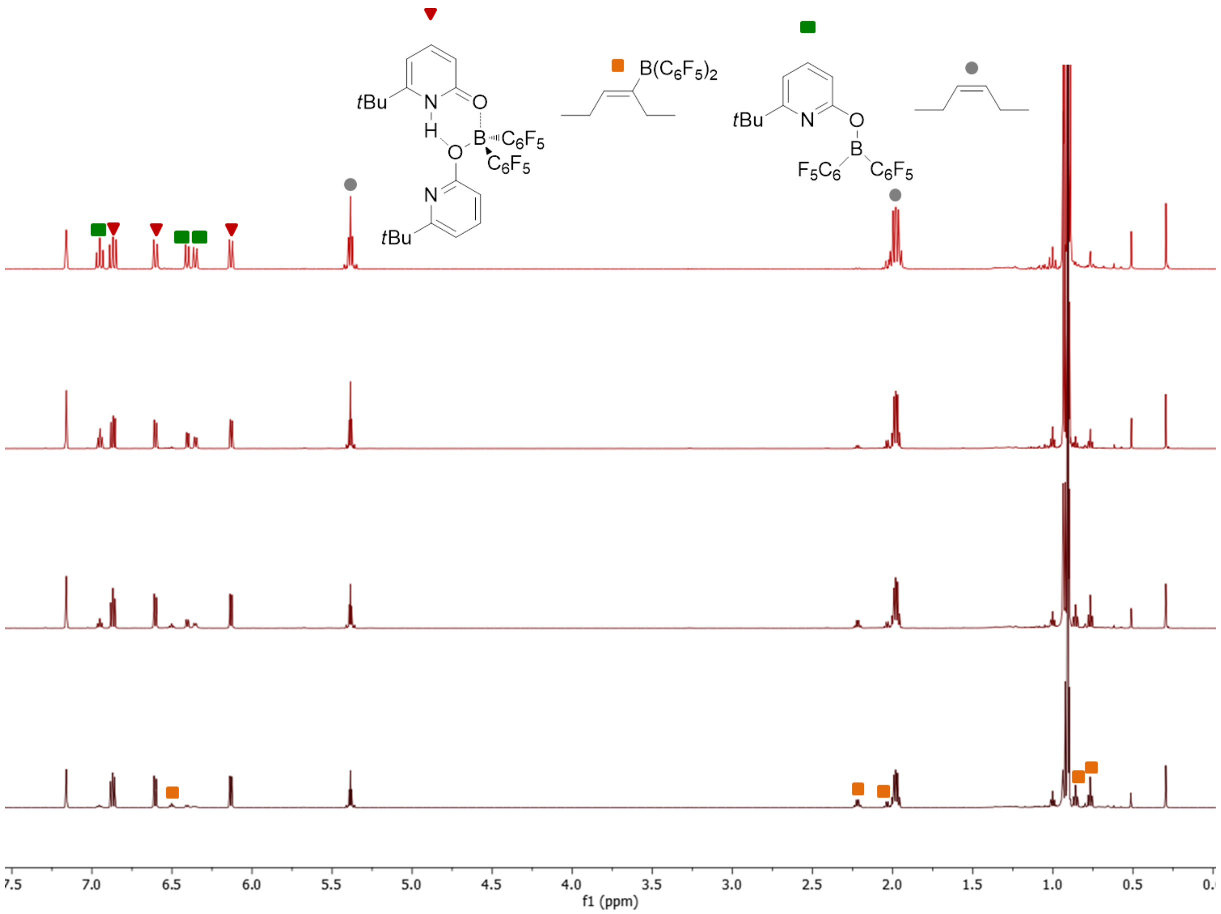

Figure SI 38: ^{1}H NMR spectra of the reaction monitoring after 7 h, 22 h, 4 d and 11 d at room temperature (600 MHz, benzene-d_6).

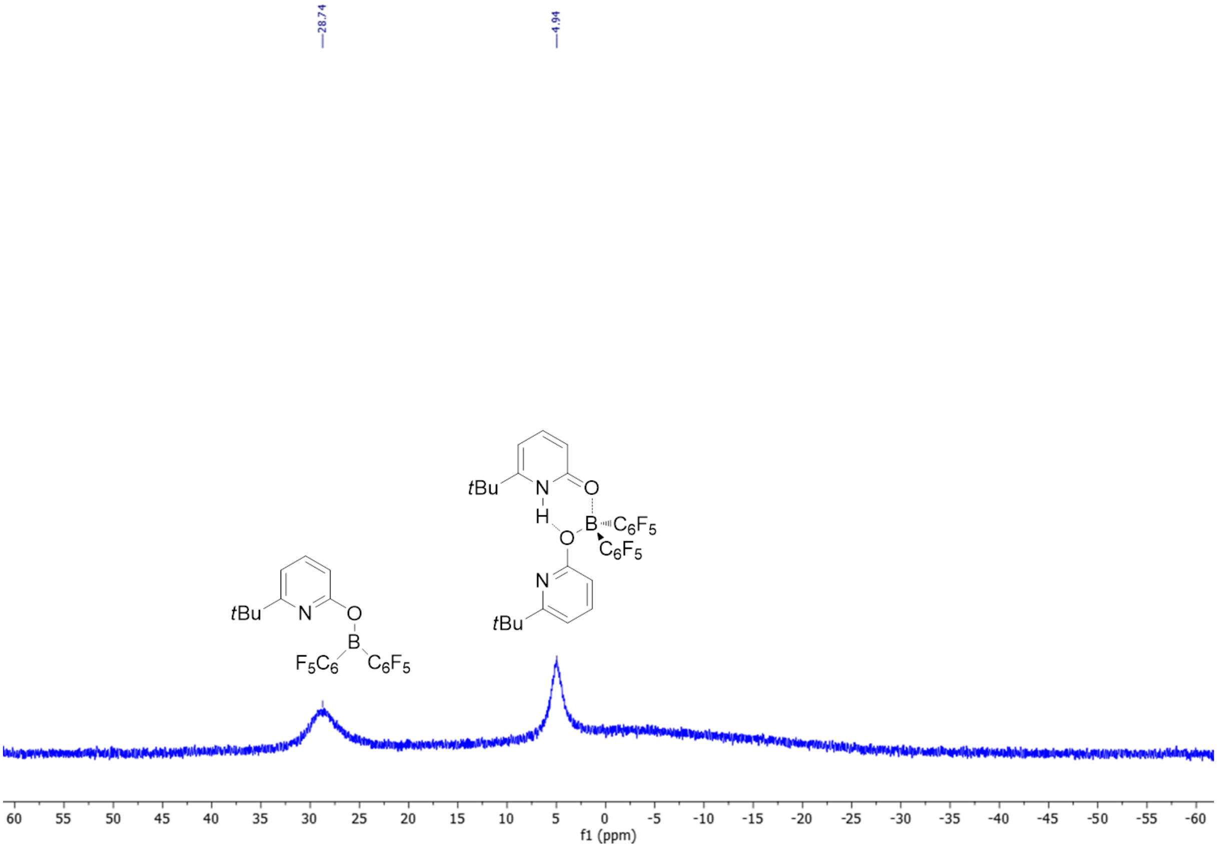

Figure SI 39: ^{11}B NMR spectra of the reaction monitoring after 11 d at room temperature (128 MHz, benzene-d_6).

3.5 Independent synthesis and characterization of the hydroboration product of 3-hexyne with Piers borane, (*Z*)-hex-3-en-3-ylbis(pentafluorophenyl)borane 9

Piers Borane (17.3 mg, 0.05 mmol) and 3-hexyne (5.7 µL, 0.05 mmol) were suspended in benzene-d_6 (0.4 ml) and the suspension was shaken until a clear colorless solution was obtained (ca. 10-15 min). The solution was transferred to an NMR tube with J Young valve and rinsed two times with benzene-d_6 (0.1 ml).

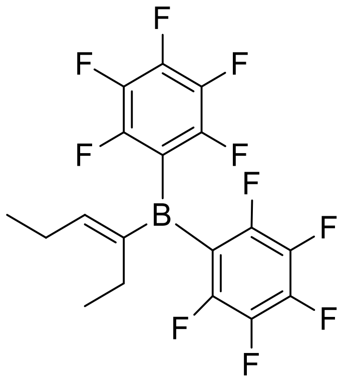

^{1}H NMR (400 MHz, benzene-d_6) δ 6.50 (t, *J* = 7.2 Hz, 1H, *H*C=CB), 2.22 (q, *J* = 7.6 Hz, 2H, $CH_3C\mathit{H}_2CB$=CH), 1.98 (p, *J* = 7.5 Hz, 2H, $CH_3C\mathit{H}_2CH$=CB), 0.86 (t, *J* = 7.6 Hz, 3H, $C\mathit{H}_3CH_2CB$=CH), 0.77 (t, *J* = 7.5 Hz, 3H, $C\mathit{H}_3CH_2CH$=CB).

^{13}C NMR (101 MHz, benzene-d_6) δ 165.0 (*C*-B), 23.7 ($H_3C\mathit{C}H_2C_{sp2}H$), 23.6 ($H_3C\mathit{C}H_2C_{sp2}B$), 15.7 ($H_3\mathit{C}CH_2C_{sp2}H$), 12.8 ($H_3\mathit{C}CH_2C_{sp2}B$).

Remark: The ^{13}C NMR spectrum shows broad signals with low intensity at 147.6, 145.1, 144.0, 141.5, 138.9, 136.4 ppm which can be tentatively assigned to the pentafluorophenyl groups. The signal of the olefinic quarternary carbon attached to the boron shows no signal in the ^{13}C NMR spectrum.

^{11}B NMR (128 MHz, benzene-d_6) δ -63.8 (s)

^{19}F NMR (377 MHz, benzene-d_6) δ -131.2 (dd, *J* = 24.5, 9.5 Hz), *ortho*-F), -149.2 (t, *J* = 20.9 Hz, *para*-F), -161.0 (td, *J* = 23.2, 9.4 Hz, *meta*-F).

3.5.1 Additional NMR spectra

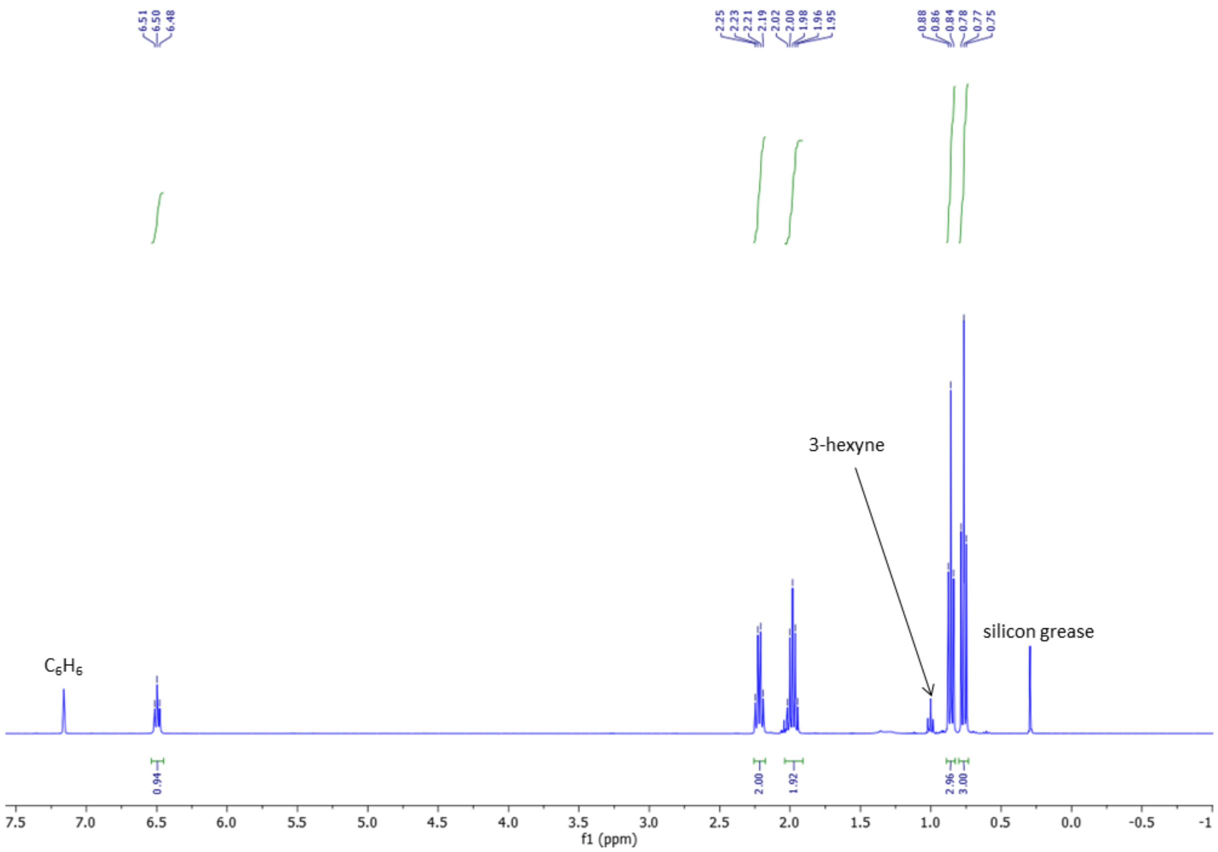

Figure SI 40: ^{1}H NMR spectrum of (*Z*)-hex-3-en-3-ylbis(perfluorophenyl)borane **9** (400 MHz, benzene-d_6).

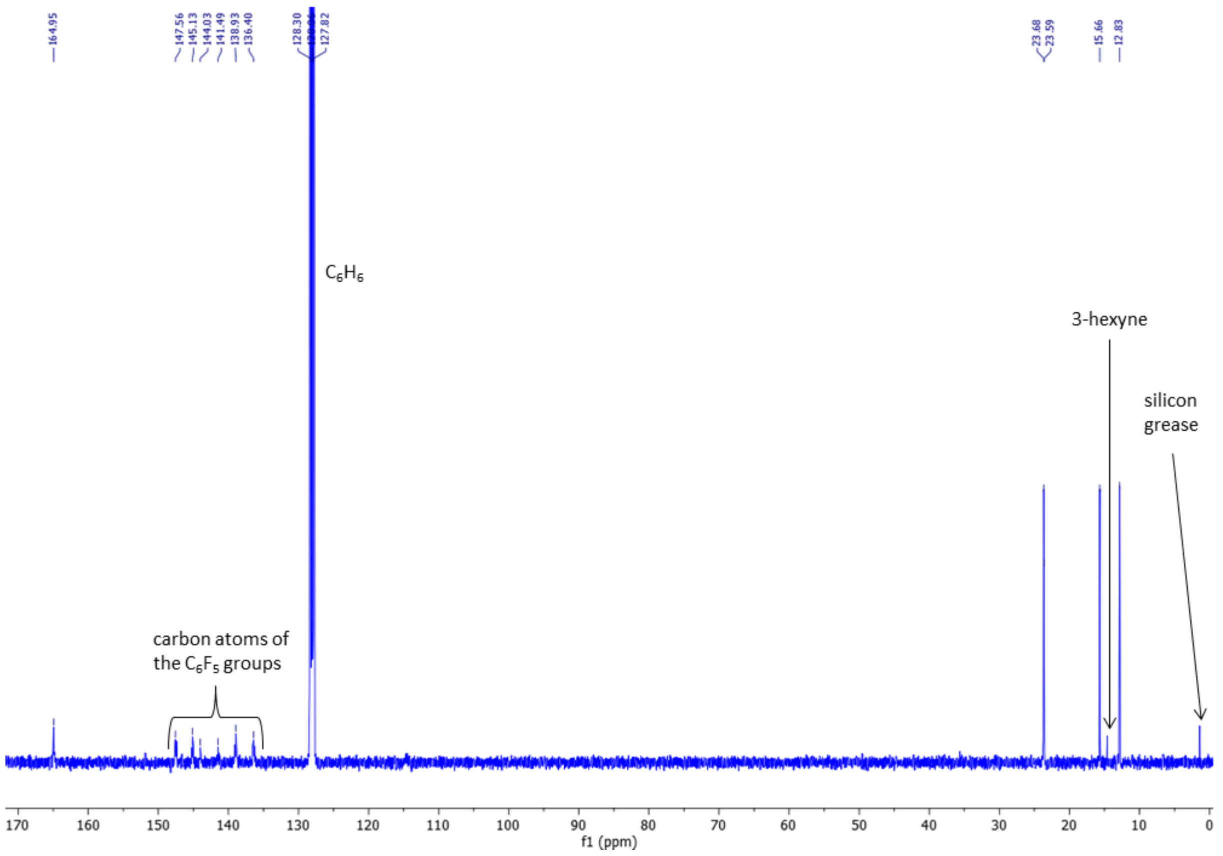

Figure SI 41: ^{13}C NMR spectrum of (*Z*)-hex-3-en-3-ylbis(perfluorophenyl)borane **9** (101 MHz, benzene-d_6).

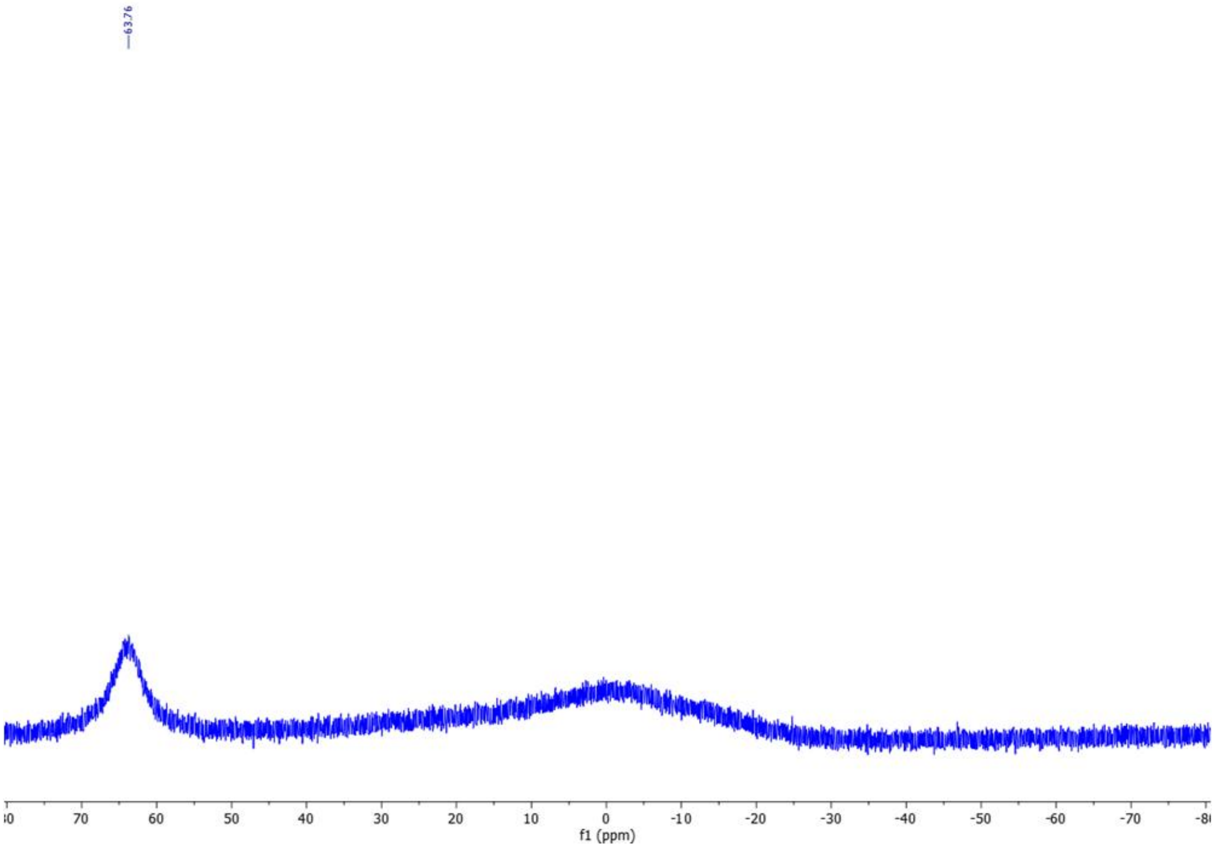

Figure SI 42: ^{11}B NMR spectrum of (*Z*)-hex-3-en-3-ylbis(perfluorophenyl)borane **9** (128 MHz, benzene-d_6).

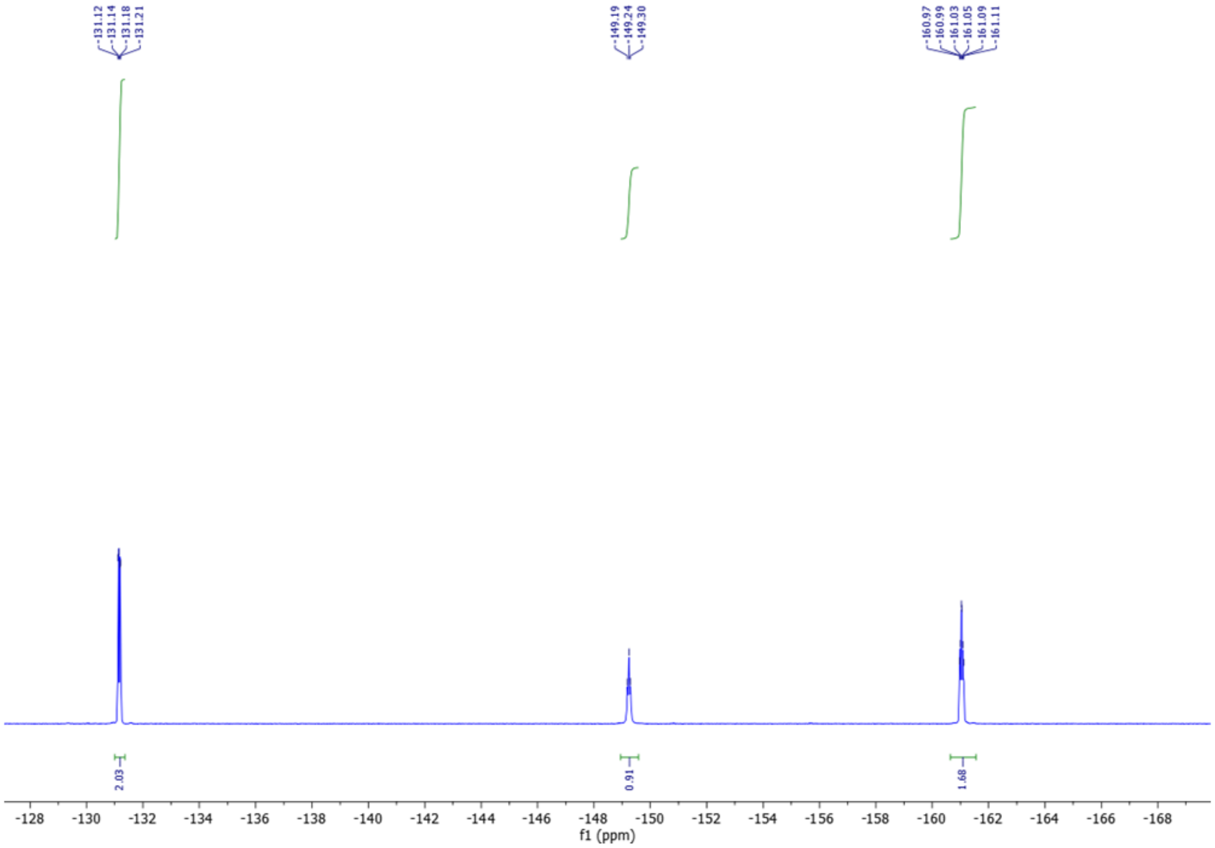

Figure SI 43: ^{19}F NMR spectrum of (*Z*)-hex-3-en-3-ylbis(perfluorophenyl)borane **9** (377 MHz, benzene-d_6).

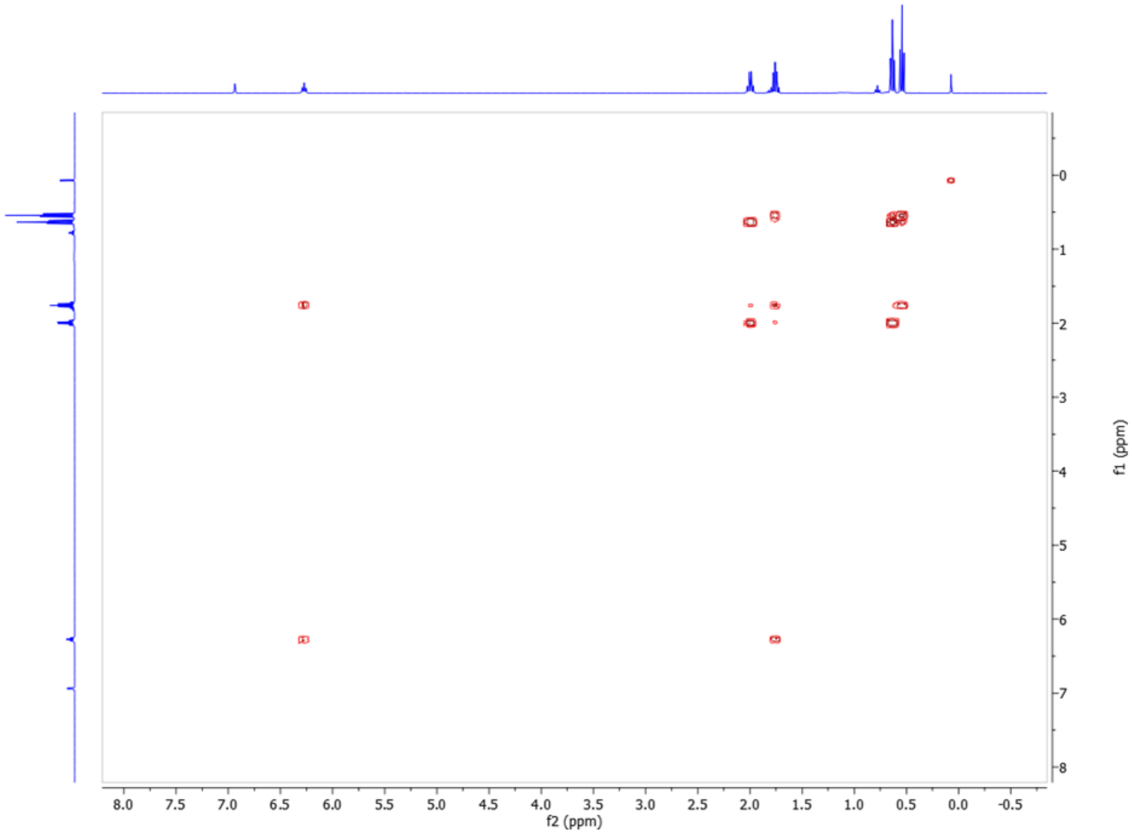

Figure SI 44: COSY NMR spectrum of (*Z*)-hex-3-en-3-ylbis(perfluorophenyl)borane **9** (400 MHz, benzene-d_6).

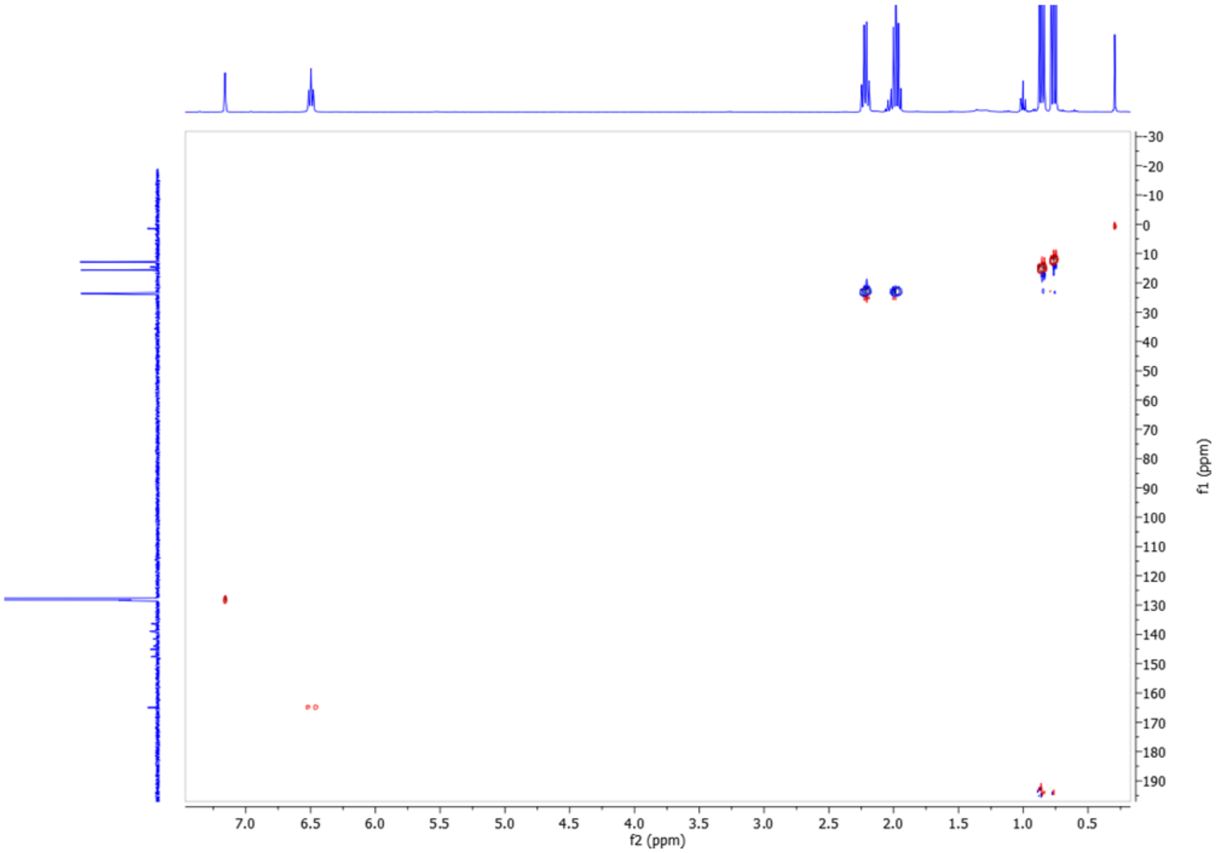

Figure SI 45: HSQC NMR spectrum of (*Z*)-hex-3-en-3-ylbis(perfluorophenyl)borane **9** (101 MHz, benzene-d_6).

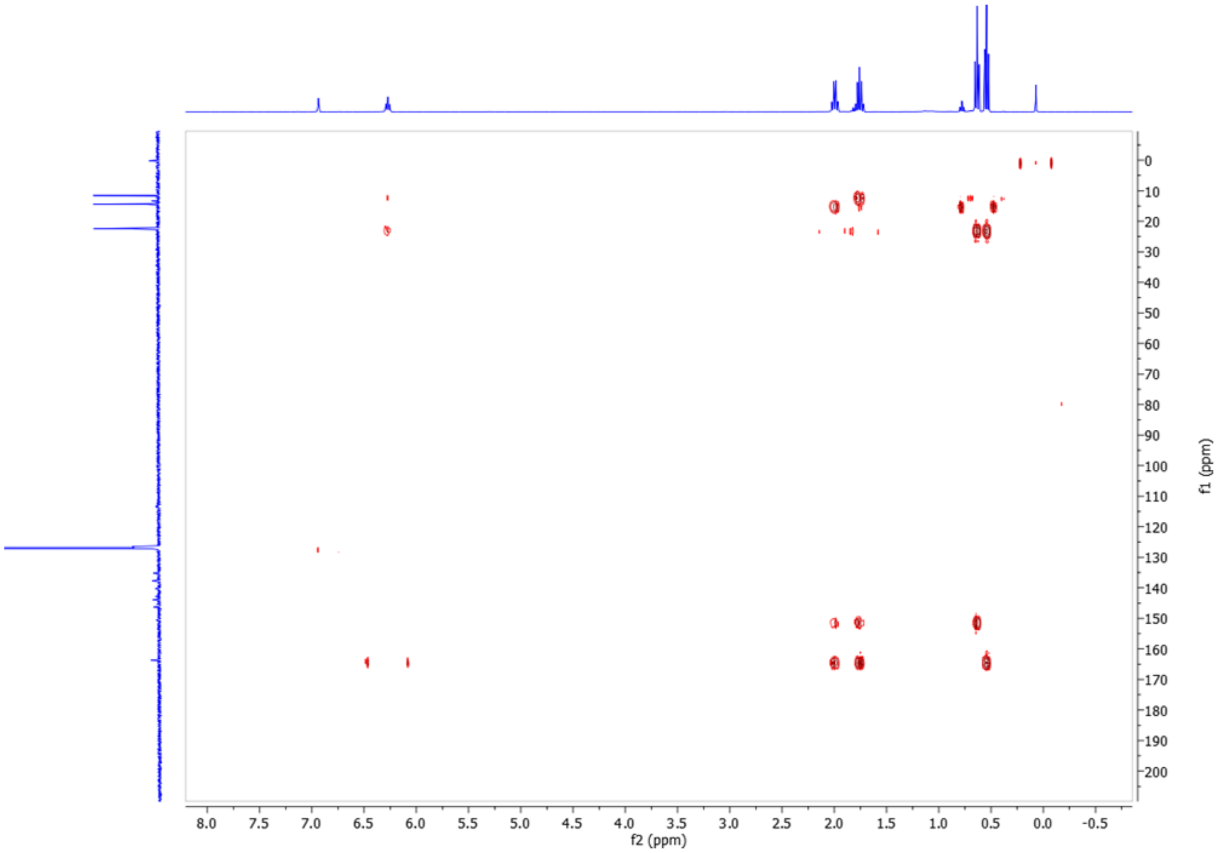

Figure SI 46: HMBC NMR spectrum of (*Z*)-hex-3-en-3-ylbis(perfluorophenyl)borane **9** (101 MHz, benzene-d_6).

3.6 Characterization of (*Z*)-hex-3-en-3-ylbis(perfluorophenyl)borane pyridone complex 10

For the synthesis see chapter 3.4.

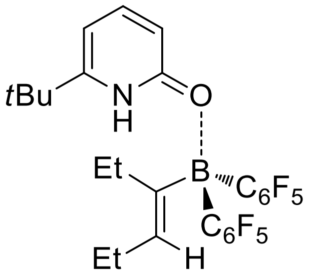

^{1}H NMR (600 MHz, benzene-d_6) δ 10.97 (s, 1H, N*H*), 6.63 (t, *J* = 8.2 Hz, 1H, Py-*H*), 6.37 (dd, *J* = 8.9, 1.2 Hz, 1H, Py-*H*), 5.72 (t, *J* = 6.4 Hz, 1H, BC=C*H*CH_2), 5.68 (d, *J* = 7.4 Hz, 1H, Py-*H*), 2.50 (q, *J* = 7.3 Hz, 2H, H_3CCH_2CB), 2.17 (p, *J* = 7.4 Hz, 2H, BC=CHCH_2CH_3), 1.06 (t, *J* = 7.5 Hz, 3H, CH_3CH_2CB=CH), 0.70 (t, *J* = 7.6 Hz, 3H, CH_3CH_2CH=CB), 0.66 (s, 9H, $C(CH_3)_3$).

^{11}B NMR (193 MHz, benzene-d_6) δ 1.68 (s).

3.6.1 Additional NMR spectra

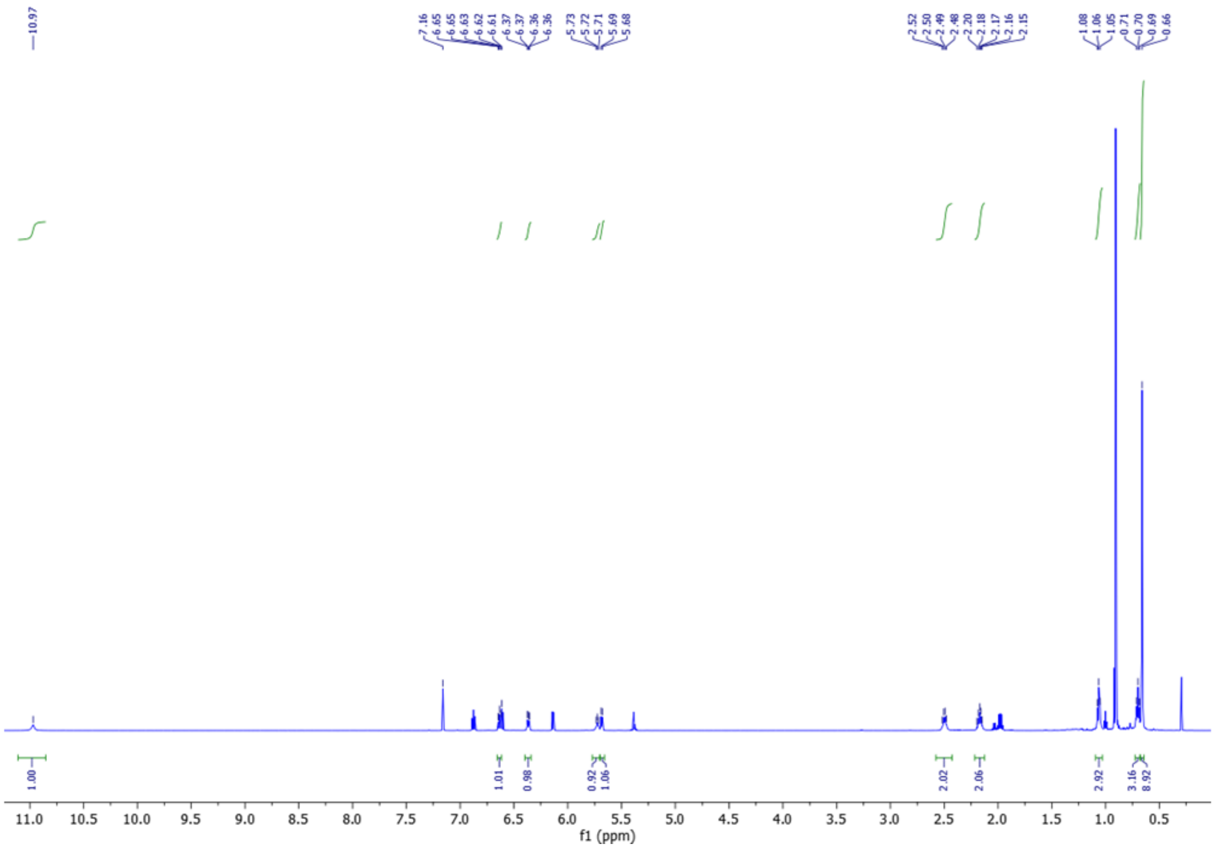

Figure SI 47: ^{1}H NMR spectrum of the reaction mixture containing complex **10**, bispyridone complex **8**, (*Z*)-hex-3-ene (600 MHz, benzene-d_6).

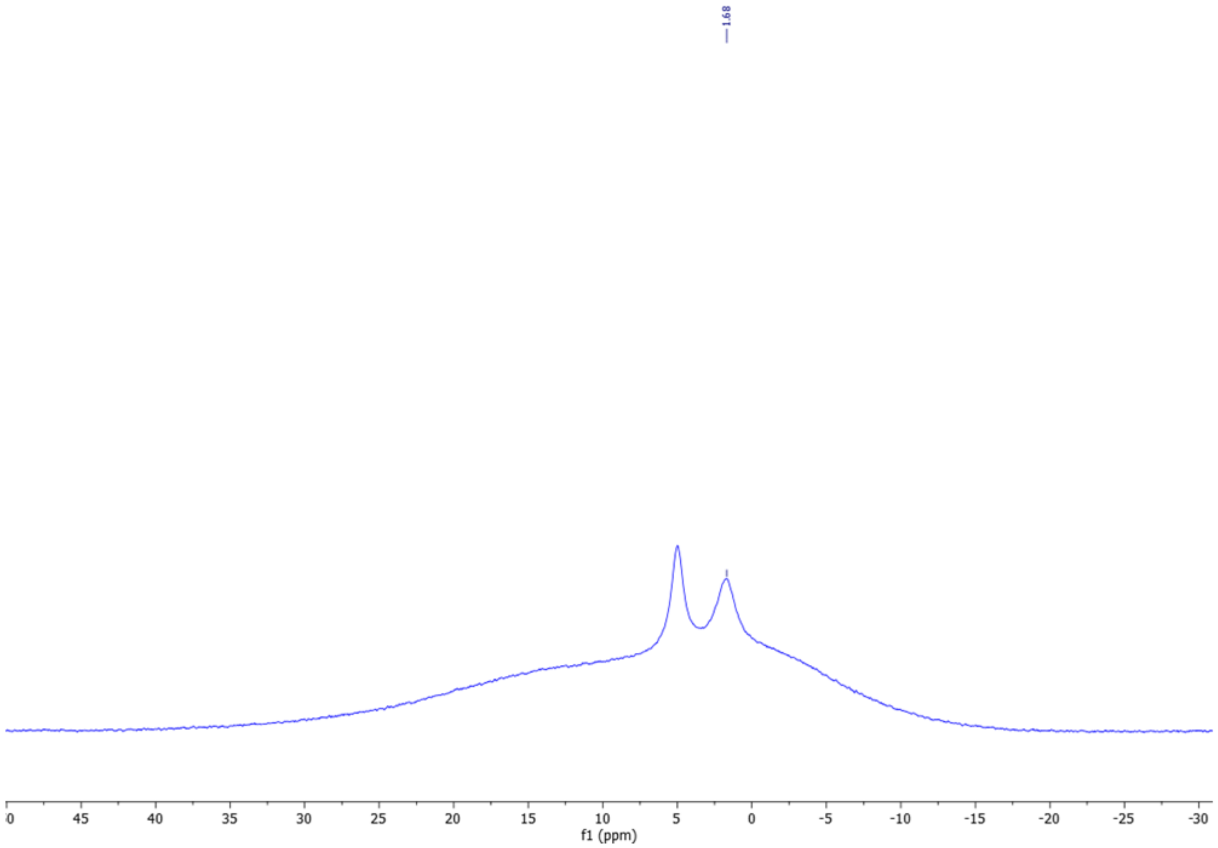

Figure SI 48: ^{11}B NMR spectrum of the reaction mixture containing complex **9**, bispyridone complex **7**, (*Z*)-hex-3-ene (193 MHz, benzene-d_6).

3.7 Independent synthesis of the hydroboration product of styrene and Piers borane

Piers Borane (10.4 mg, 0.030 mmol) was suspended in benzene-d_6 (0.4 ml). Styrene (3.4 µL, 0.030 mmol) was added and the suspension was shaken until a clear solution was obtained. The solution was transferred to an NMR tube with a J Young valve.

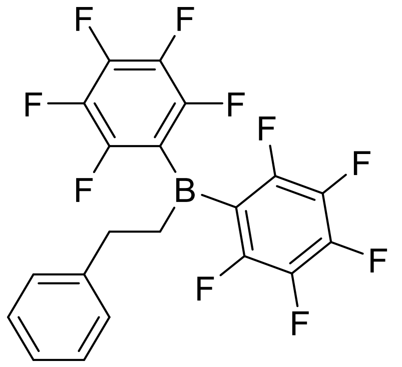

^{1}H NMR (600 MHz, benzene-d_6) δ 7.12 (t, J = 7.6 Hz, 2H, Ph-H), 7.05-7.00 (m, 3H, Ph-H), 2.69 (t, J = 7.7 Hz, CH_2), 2.20 (t, J = 7.9 Hz, CH_2).

^{13}C NMR (151 MHz, benzene-d_6) δ 142.4 (C_q), 128.2 (CH), 128.0 (CH), 125.0 (CH), 32.9 (CH$_2$), 30.6 (CH$_2$)

Remark: The ^{13}C NMR spectrum shows broad signals with low intensity at 147.5, 145.9, 138.1, 136.4 ppm which can be tentatively assigned to the pentafluorophenyl groups.

^{11}B NMR (192 MHz, benzene-d_6) δ 73.1 (s).

^{19}F NMR (377 MHz, benzene-d_6) δ -130.3 (dt, J = 20.0, 7.1 Hz, *ortho*-F), -147.1 (t, J = 20.1 Hz, *para*-F), -160.8 - -161.0 (m, *meta*-F).

3.7.1 Additional NMR spectra

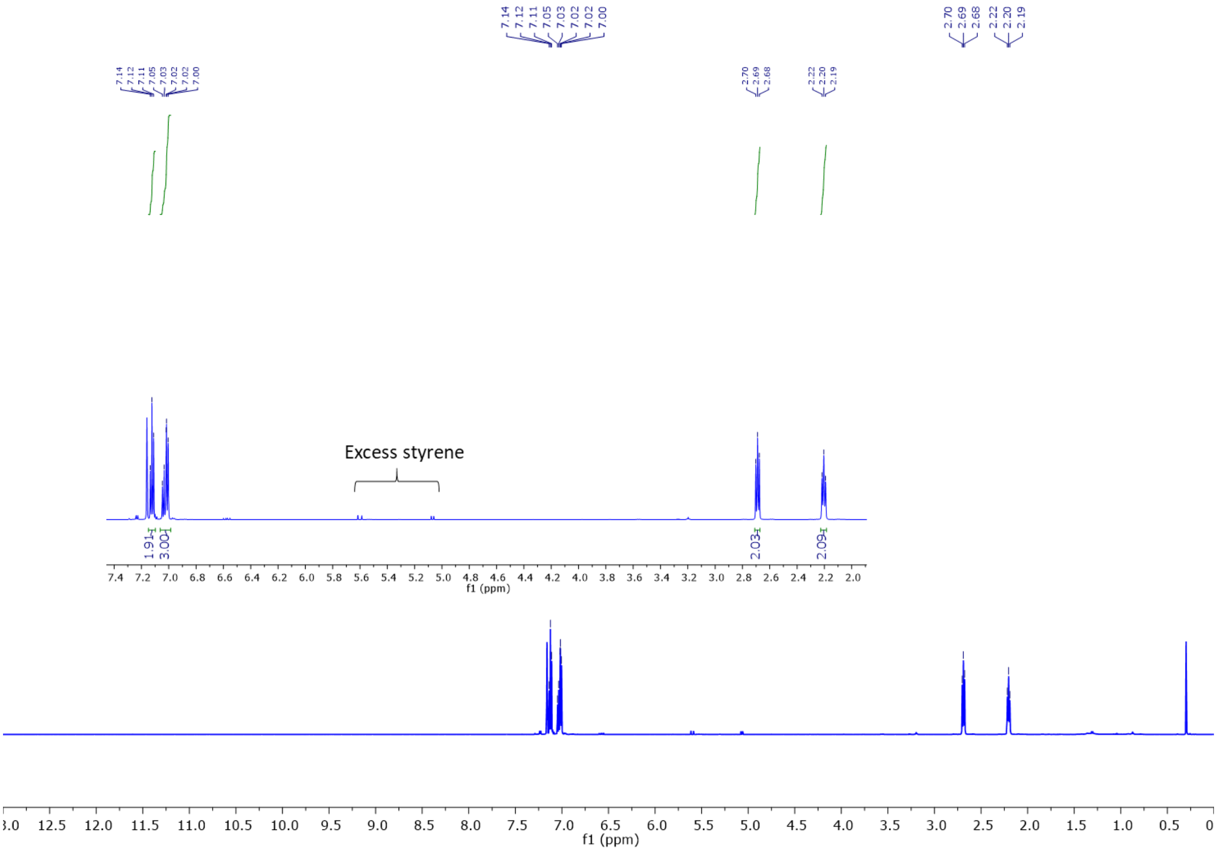

Figure SI 49: ^{1}H NMR spectrum of the hydroboration of styrene with Piers borane. (600 MHz, benzene-d_6)

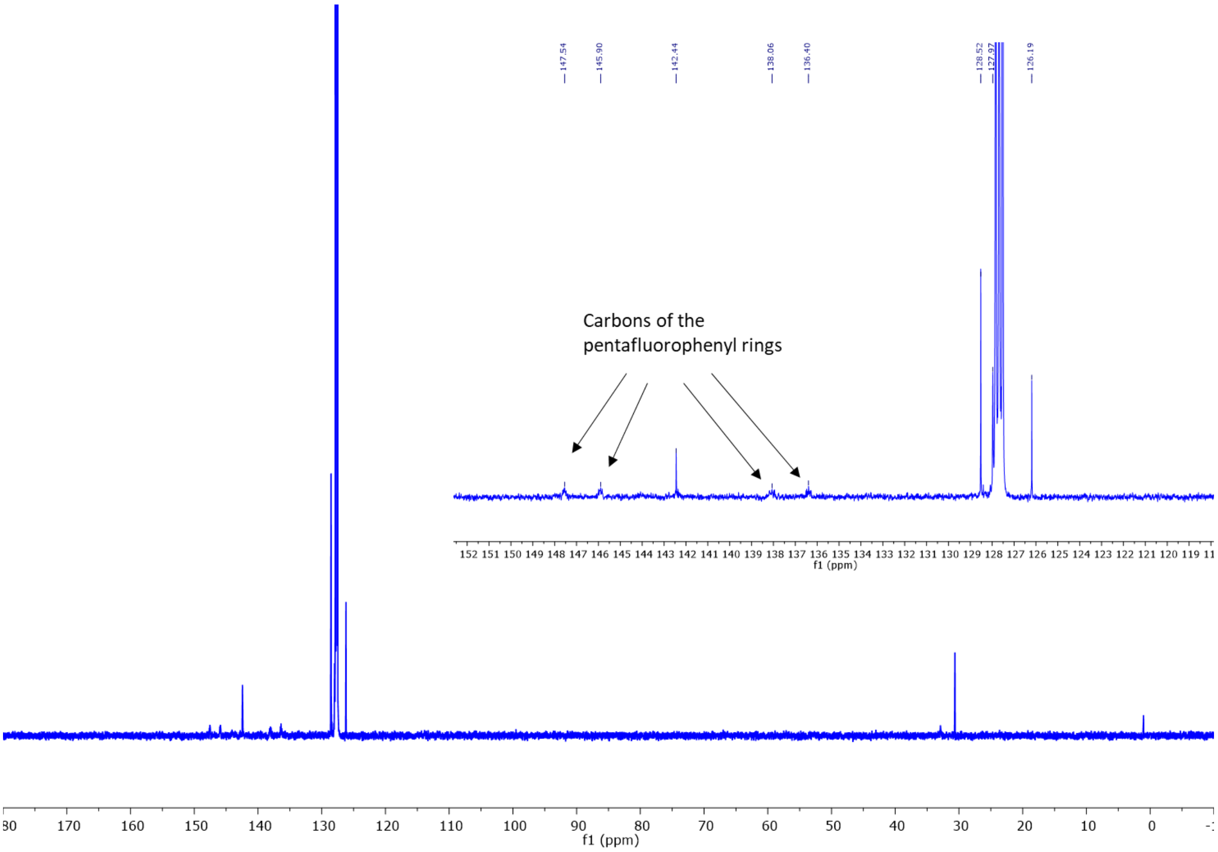

Figure SI 50: ^{13}C-NMR spectrum of the hydroboration of styrene with Piers borane. (151 MHz, benzene-d_6)

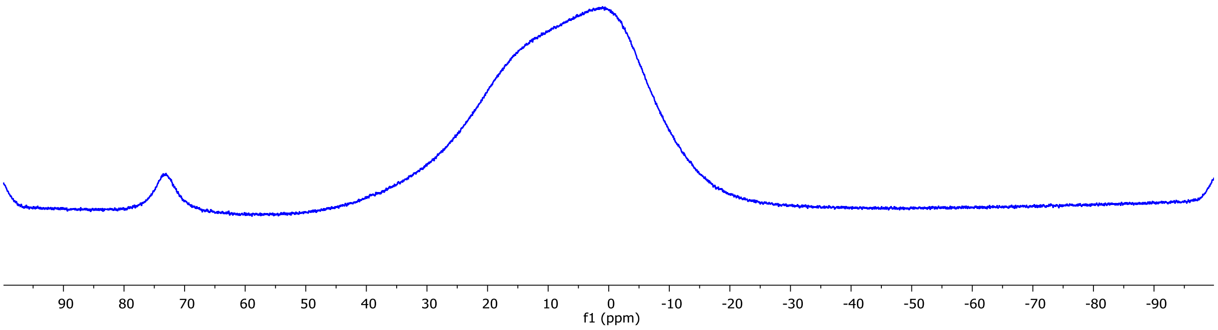

Figure SI 51: ^{11}B NMR spectrum of the hydroboration of styrene with Piers borane. (192 MHz, benzene-d_6)

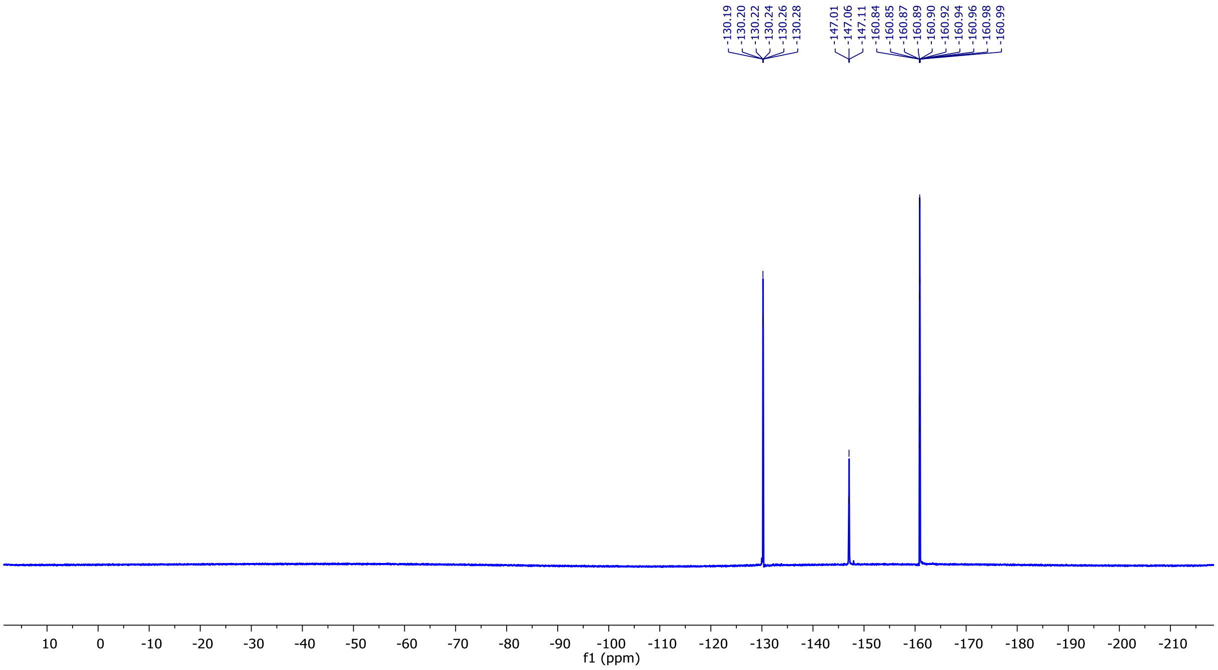

Figure SI 52: ^{19}F NMR spectrum of the hydroboration of styrene with Piers borane. (377 MHz, benzene, d_6)

3.8 Synthesis of the hydroborated styrene's 6-*tert*-2-pyridone complex 7

Piers Borane (10.4 mg, 0.030 mmol) was suspended in benzene-d_6 (0.4 ml). Styrene (3.44 µL, 0.030 mmol) was added and the suspension was shaken until a clear solution was obtained. Pyridone **5** (4.5 mg, 0.030 mmol) was added and the solution was transferred to an NMR tube with J Young valve.

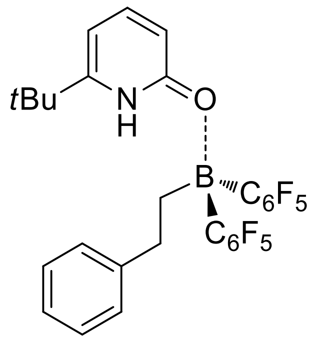

^{1}H NMR (400 MHz, benzene-d_6) δ 9.3 (bs, N*H*), 7.24 (d, *J* = 7.8 Hz, 2H, Ph-*H*), 7.08 (t, *J* = 7.4 Hz, 2H, Ph-*H*), 6.95 (t, *J* = 7.3 Hz, 1H, Ph-*H*), 6.50 (dd, *J* = 8.0, 7.40 Hz, 1H, Py-*H*), 6.35 (ddd, *J* = 9.0, 2.3, 0.8 Hz, 1H, Py-*H*), 5.49 (ddd, *J* = 7.4 Hz, 2.1, 1.0 Hz, 1H, Py-*H*), 2.76 (t, *J* = 8.4 Hz, CH_2), 1.96 (t, *J* = 8.1 Hz, CH_2), 0.41 (s, 9H, C(CH_3)$_3$).

^{13}C NMR (101 MHz, benzene-d_6) δ 160.7 (C_q), 156.8 (C_q), 146.2 (C_q), 145.4 (Pyr-*C*), 128.2 (Ph-*C*), 128.0 (Ph-*C*), 125.0 (Ph-*C*), 125.0 (Ph-*C*), 113.9 (Pyr-*C*), 108.0 (Pyr-*C*) , 34.0 (*C*H_2)), 32.7 (*C*H_2), 27.3 (C(*C*H_3)$_3$

Remark: The ^{13}C NMR spectrum shows broad signals with low intensity from 150 ppm to 135 ppm which can be tentatively assigned to the pentafluorophenyl groups.

^{11}B NMR (128 MHz, benzene-d_6) δ 3.3 (s).

^{19}F NMR (377 MHz, benzene-d_6) δ 133.4 (dd, *J* = 24.4, 9.5 Hz, *ortho*-F), -158.3 (d *J* = 20.5 Hz, *para*-F), -163.7 - -163.9 (m*, meta*-F).

3.8.1 Additional NMR spectra

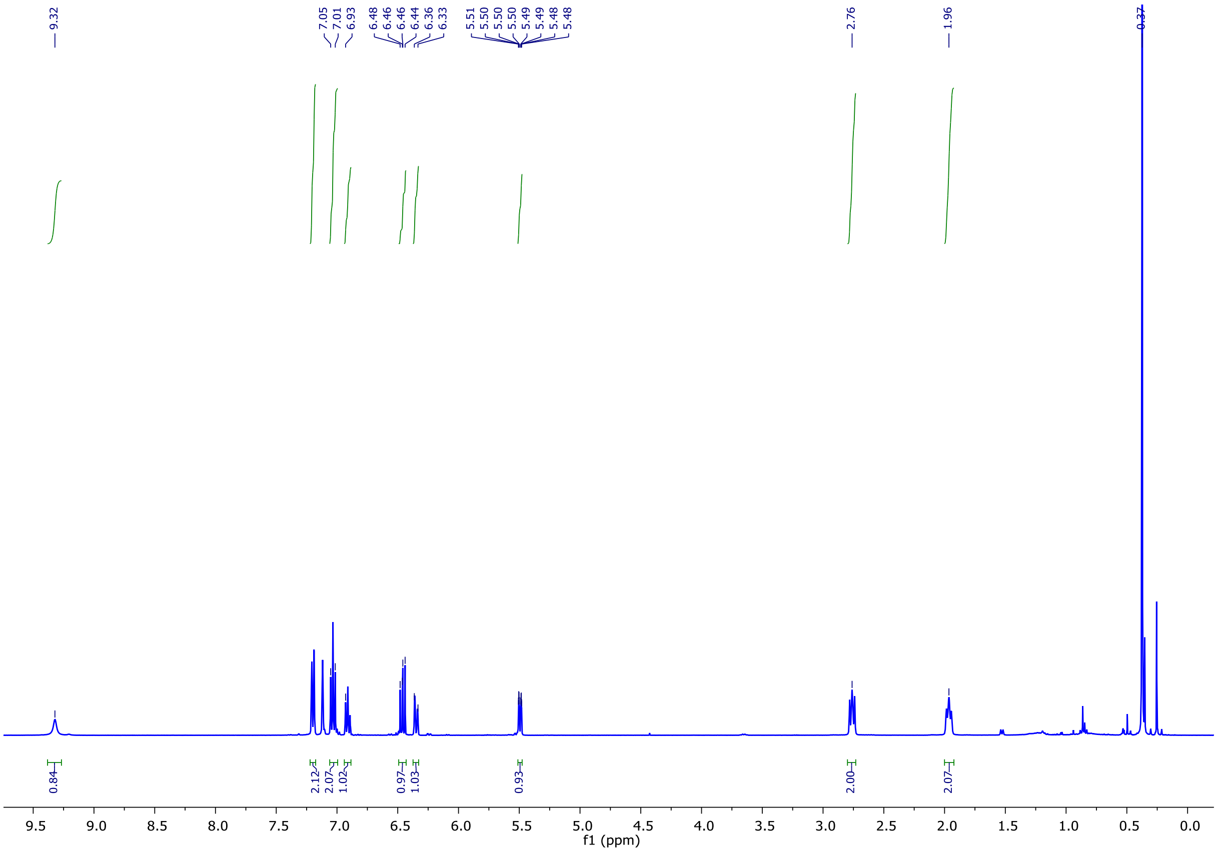

Figure SI 53: ^{1}H NMR spectrum of complex **7** (400 MHz, benzene-d_6).

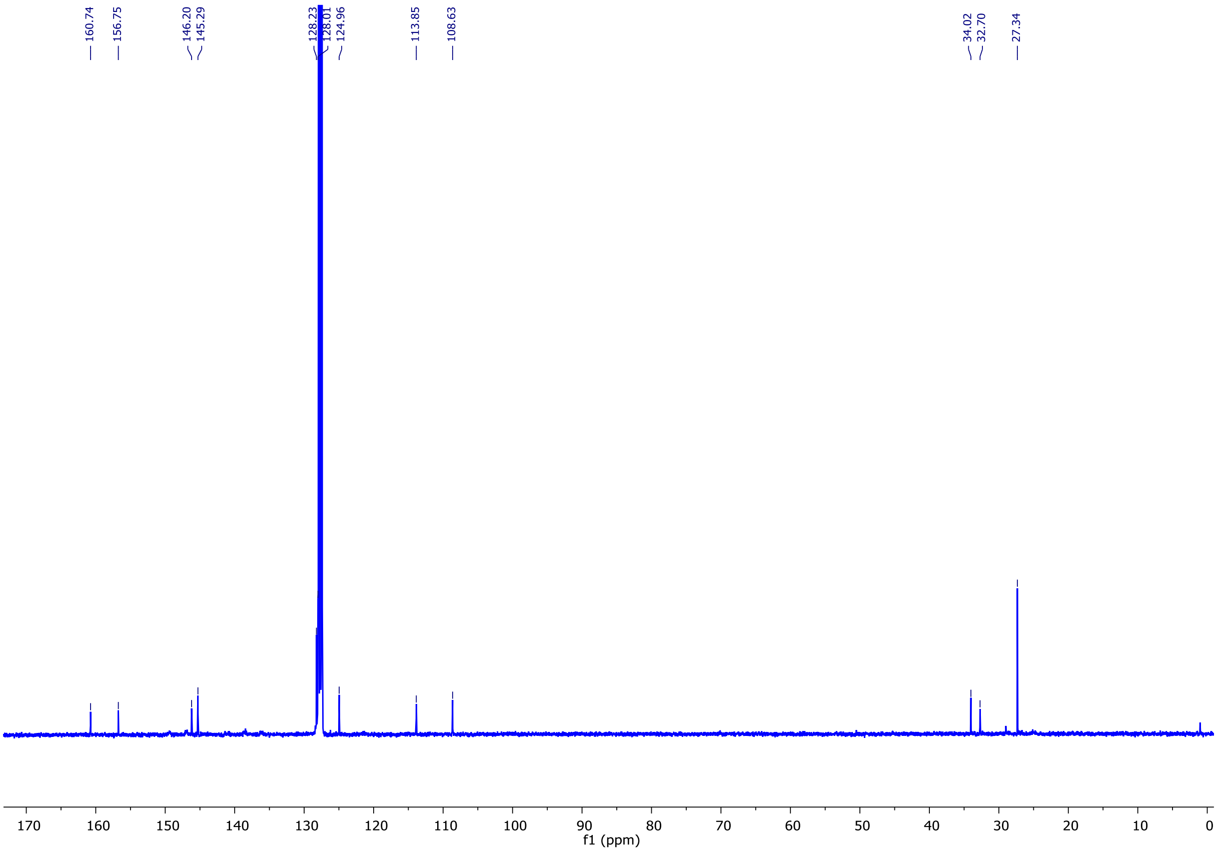

Figure SI 54: ^{13}C NMR spectrum of the complex **7** (101 MHz, benzene-d_6).

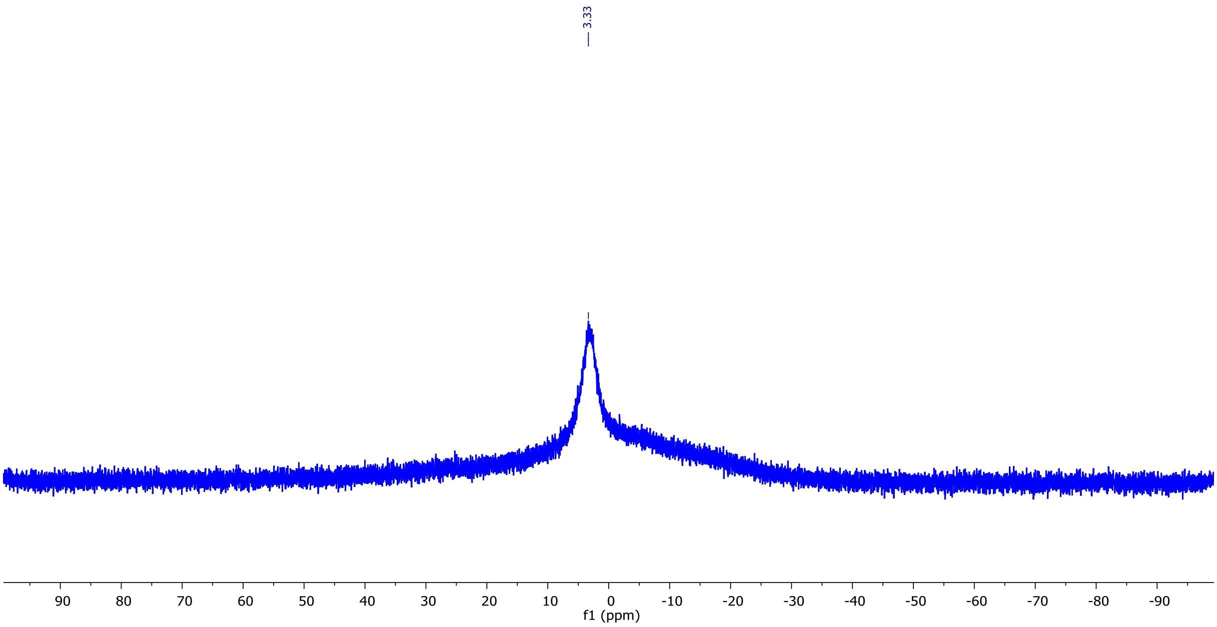

Figure SI 55: ^{11}B NMR spectrum of complex **7** (128 MHz, benzene-d_6).

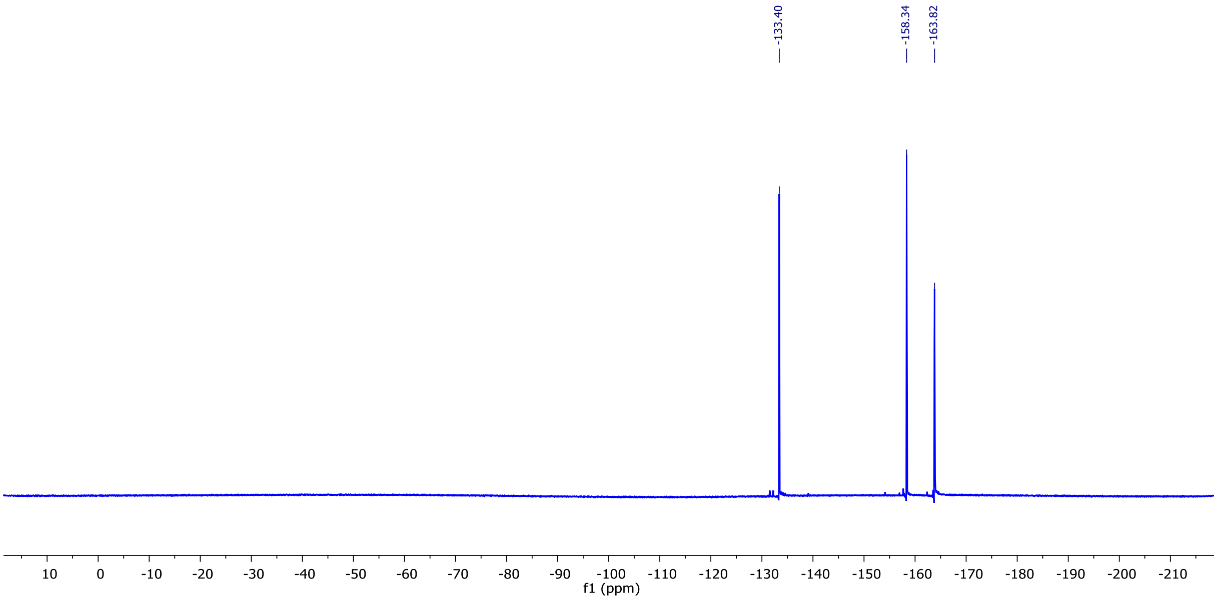

Figure SI 56: ^{19}F NMR spectrum of complex **7** (377 MHz, benzene-d_6).

3.9 Investigation of the C–H Activation of terminal alkynes by the boroxypyridine 3

It was investigated whether the product of C_{sp}-H activation by boroxypyridine **3** and a terminal alkyne is reversible.

Piers borane (10.4 mg, 0.030 mmol) and pyridone **5** (4.5 mg, 0.030 mmol) were added to an NMR tube with a J Young valve. Benzene-d_6 (0.4 ml) was added and the tube was shaken until a clear solution was obtained. The NMR tube was degassed using three freeze-pump-thaw cycles and placed in a 60 °C oil bath overnight under passive vacuum. The NMR tube was degassed again three times and a ^{1}H NMR spectrum was recorded.

Then, cyclohexylacetylene (4.23 µL, 0.034 mmol) was added and after 30 min at room temperature a ^{1}H NMR spectrum was measured (Figure SI 57).

Trace amounts of hydrogenation product and carboboration product were observed presumably due to slight excess of cyclohexylacetylene and Piers borane.

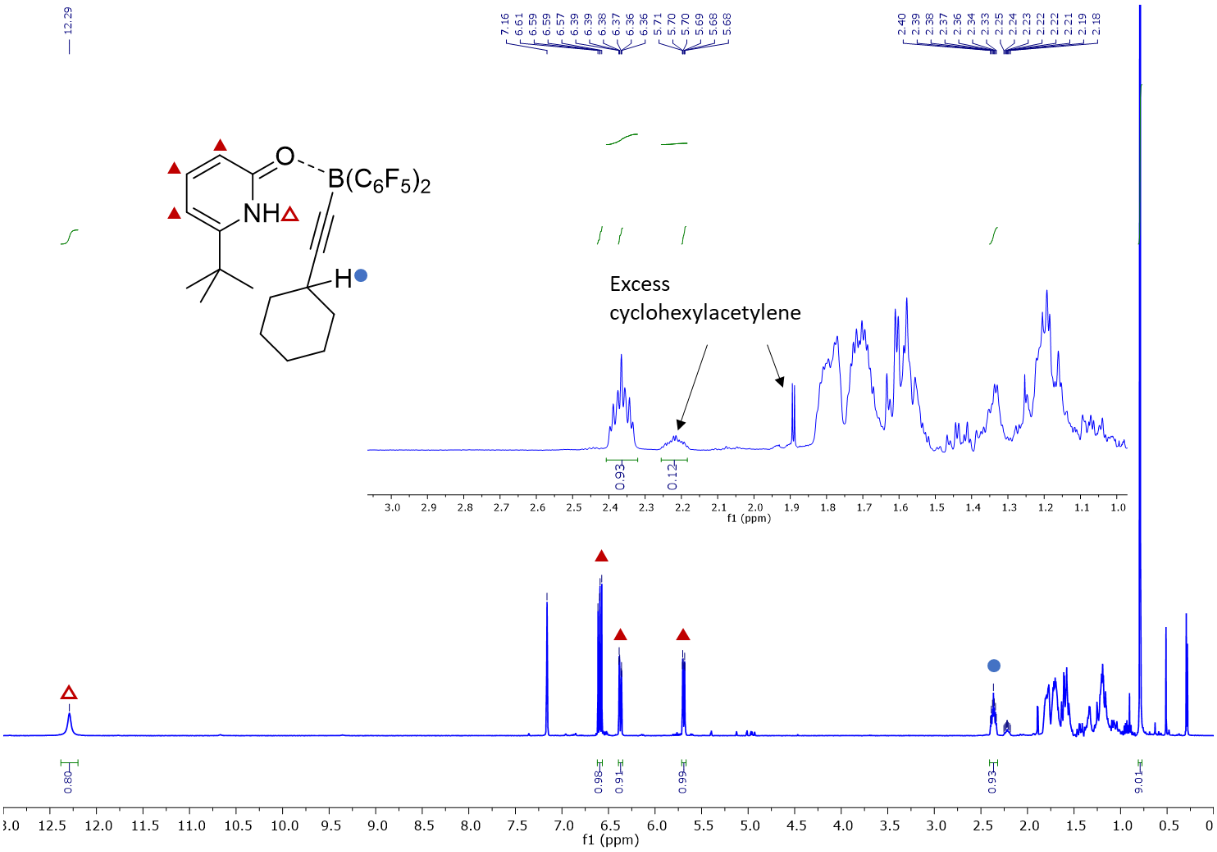

Figure SI 57: ^{1}H NMR of C–H activation of cyclohexylacetylene by boroxypyridine **3** with high field excerpt (400 MHz, benzene-d_6).

Then, phenylacetylene (3.73 µL, 0.034 mmol) was added to the NMR tube and a ^{1}H NMR spectrum was measured (Figure SI 58). The formation of the corresponding C-H activation product was not observed at room temperature. The NMR tube was placed in an oil bath preheated to 80 °C for 1 h and another ^{1}H NMR spectrum was measured.

A new set of signals was observed that can be assigned to the C-H activation product of the boroxypyridine **3** and phenylacetylene, as reported in the literature.[6] This is supported by the C_{sp}–H signal of phenylacetylene decreasing and the signals of cyclohexylacetylene increasing in intensity (Figure SI 59).

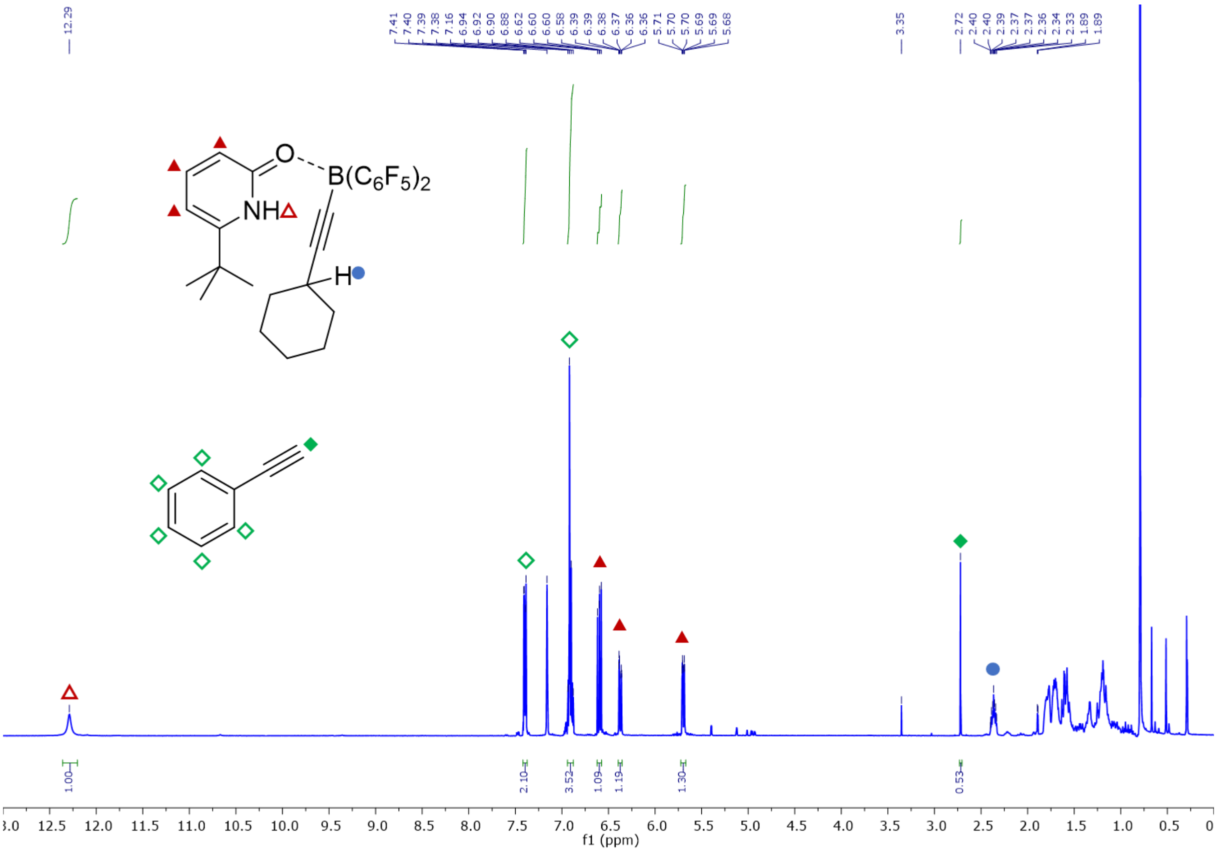

Figure SI 58: ^{1}H NMR after the addition of phenylacetylene to the NMR tube at room temperature (400 MHz, benzene-d_6).

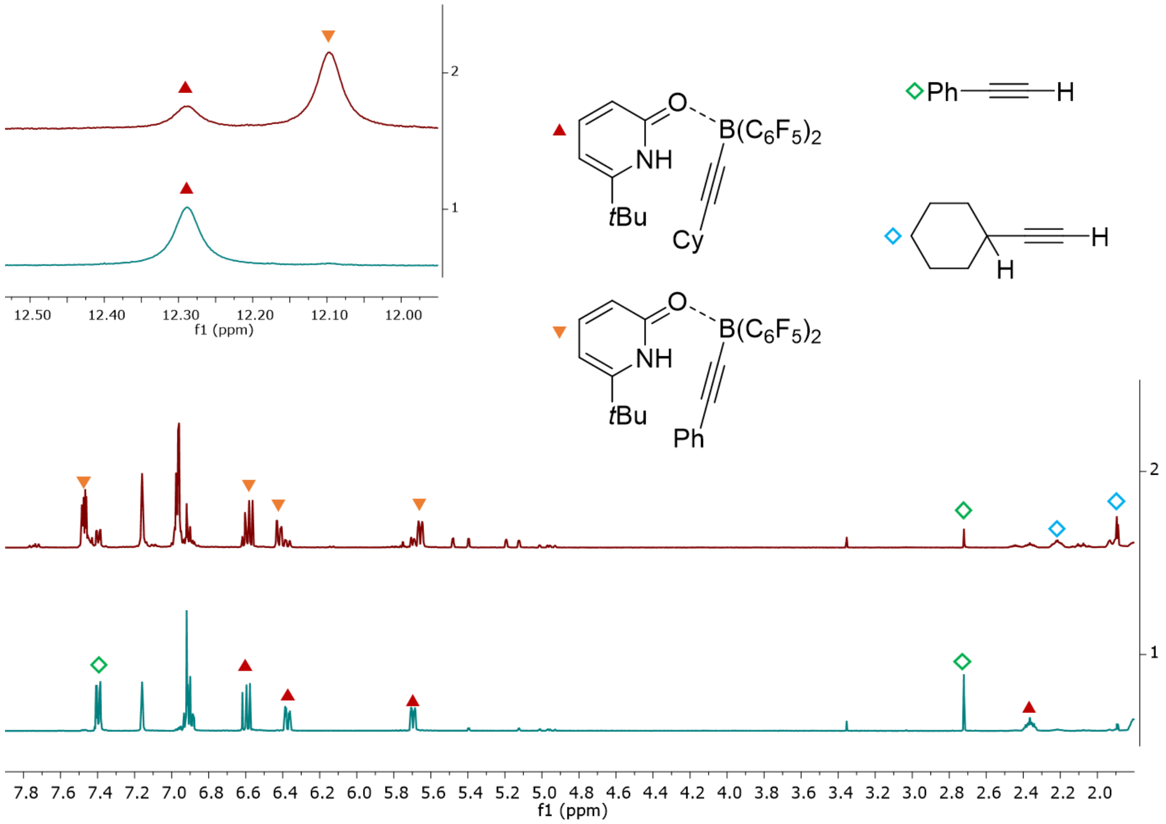

Figure SI 59: ^{1}H NMR spectra before heating (teal, bottom) and after 1 h at 80 °C (red, top) with excerpts of the N*H* signals (400 MHz, benzene-d_6).

4 NMR spectra of the catalysis experiments

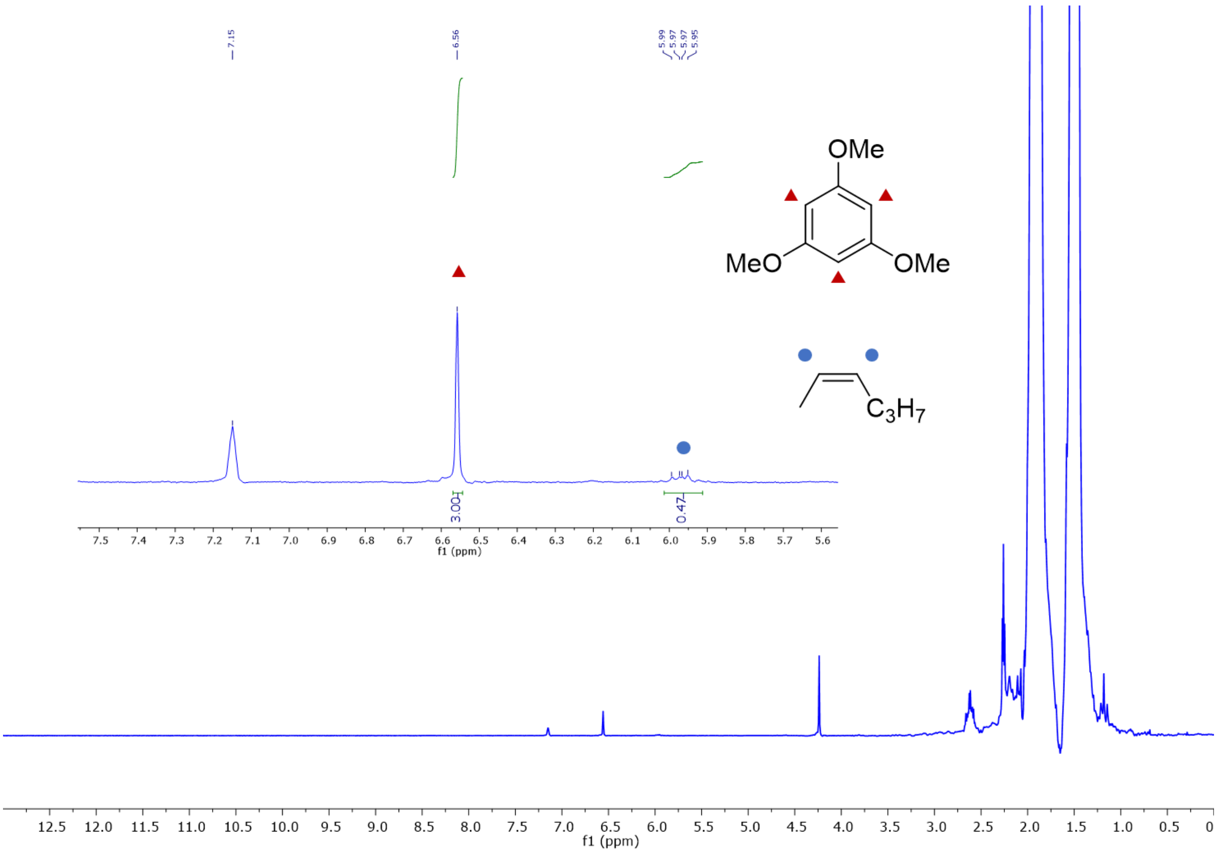

Figure SI 60: ^{1}H NMR spectrum of the hydrogenation of 2-hexyne using Piers borane (6.5 mol%) only with 1,3,5-trimethoxybenzene (8.4 mg, 0.050 mmol) as internal standard (400 MHz, *n*-hexane with benzene-d_6 capillary). Trace amounts of product detectable (<1%).

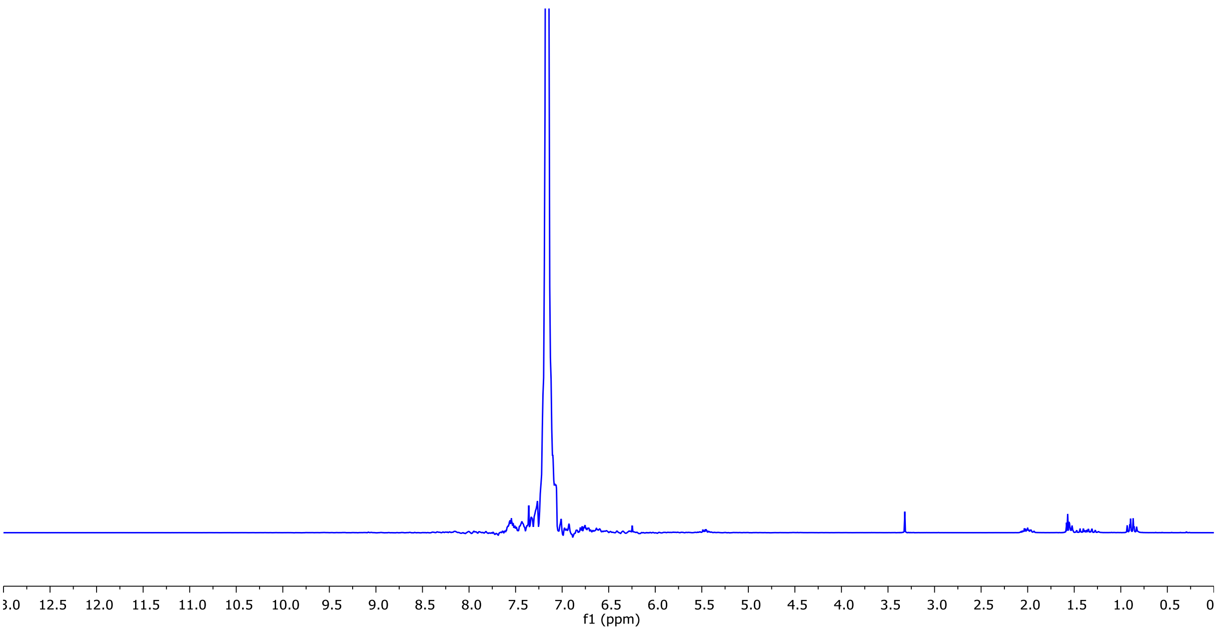

Figure SI 61: ^{1}H NMR spectrum of the hydrogenation of 2-hexyne using standard conditions in benzene with 1,3,5-trimethoxybenzene (8.4 mg, 0.050 mmol) as internal standard (200 MHz, *n*-hexane with benzene-d_6 capillary).

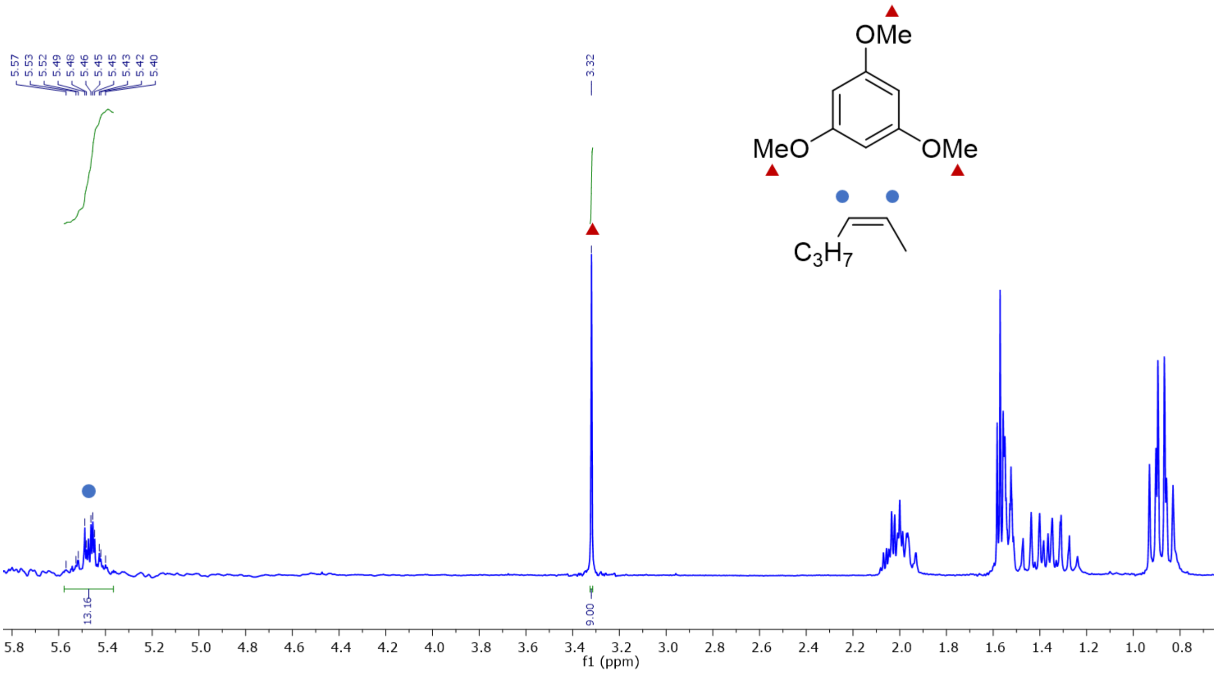

Figure SI 62: High field excerpt of the hydrogenation of 2-hexyne using standard conditions in benzene with 1,3,5-trimethoxybenzene (8.4 mg, 0.050 mmol) as internal standard (200 MHz, *n*-hexane with benzene-d_6 capillary). NMR yield: 67%.

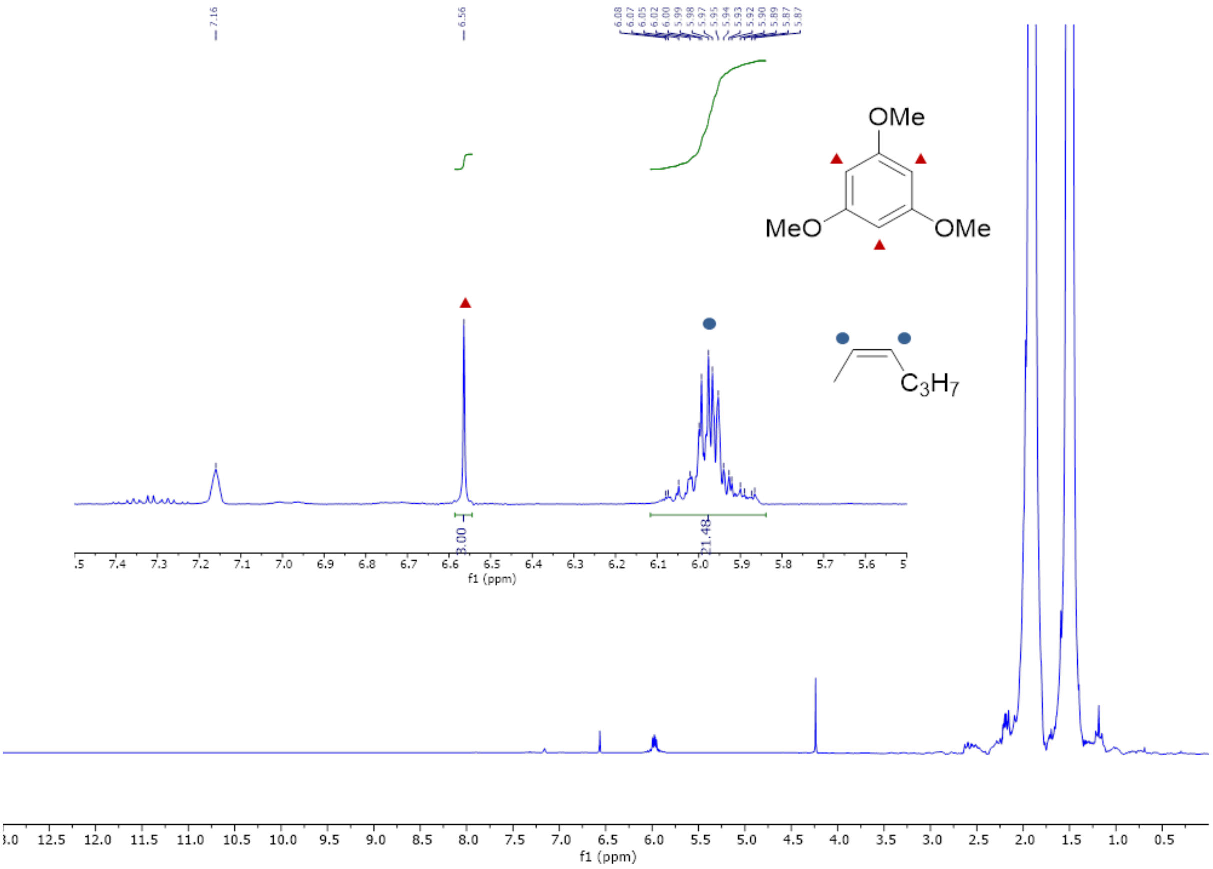

Figure SI 63: ^{1}H NMR spectrum of the hydrogenation of 2-hexyne with 1,3,5-trimethoxybenzene (8.4 mg, 0.050 mmol) as internal standard (200 MHz, *n*-hexane with benzene-d_6 capillary). NMR yield: 90%.

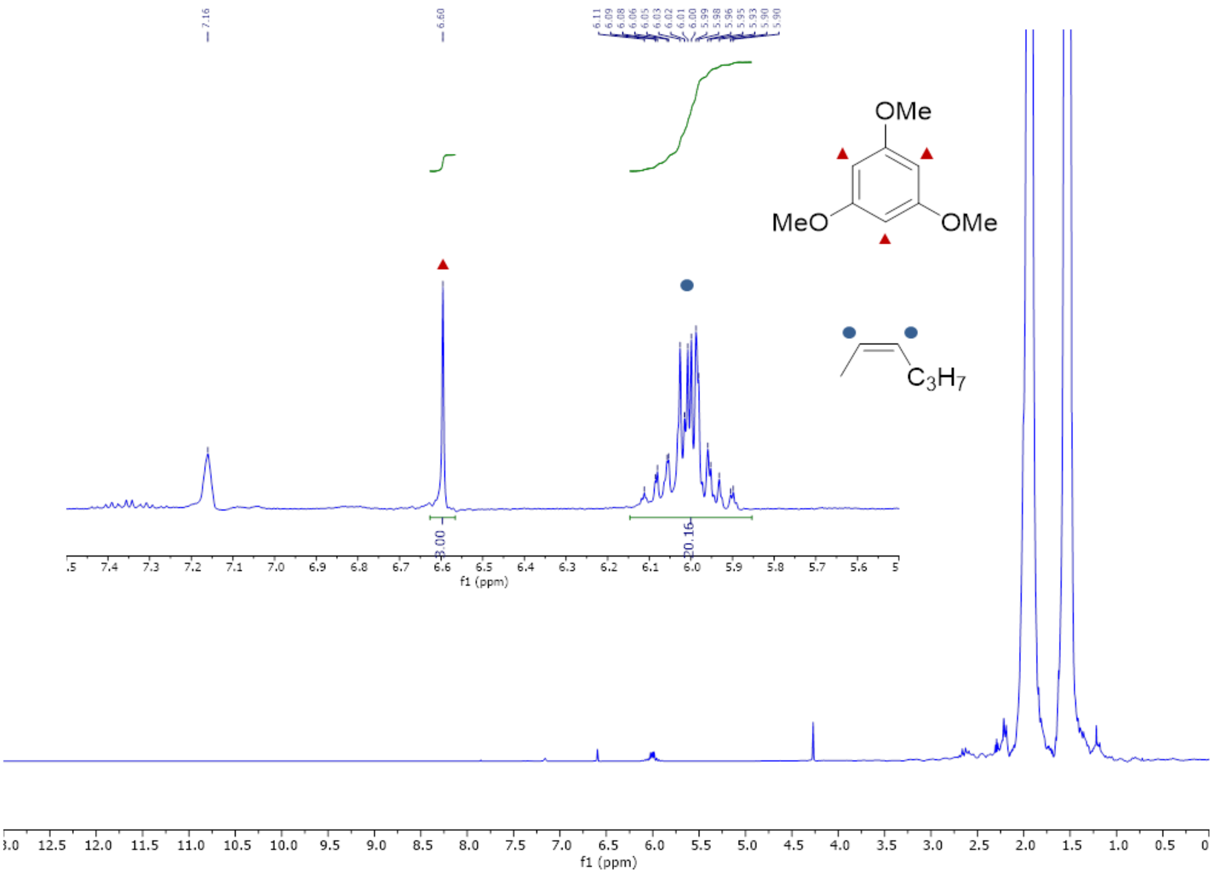

Figure SI 64: ^{1}H NMR spectrum of the hydrogenation of 2-hexyne using standard conditions with 1,3,5-trimethoxybenzene (8.4 mg, 0.050 mmol) as internal standard (200 MHz, *n*-hexane with benzene-d_6 capillary). NMR yield: 84%.

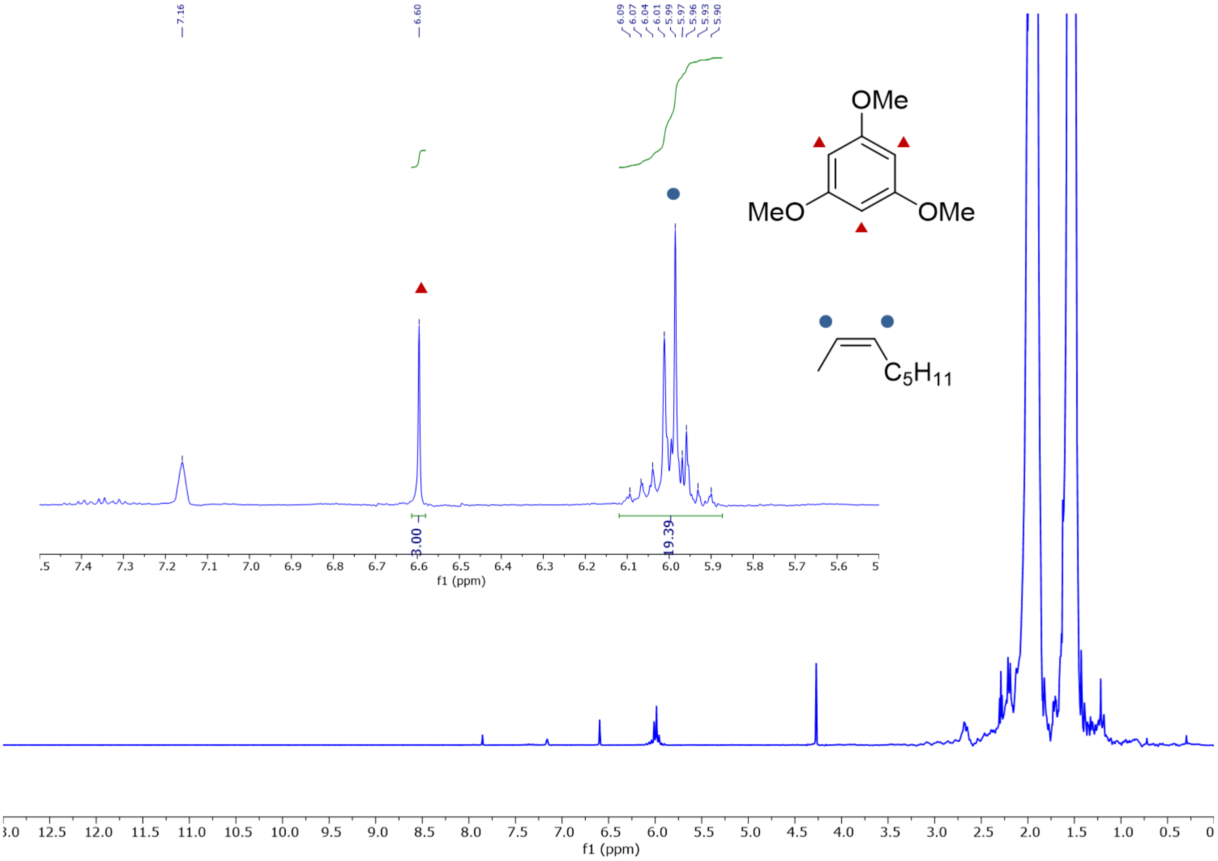

Figure SI 65: ^{1}H NMR spectrum of the hydrogenation of 2-octyne using standard conditions with 1,3,5-trimethoxybenzene (8.4 mg, 0.050 mmol) as internal standard (200 MHz, *n*-hexane with benzene-d_6 capillary). NMR yield: 81%.

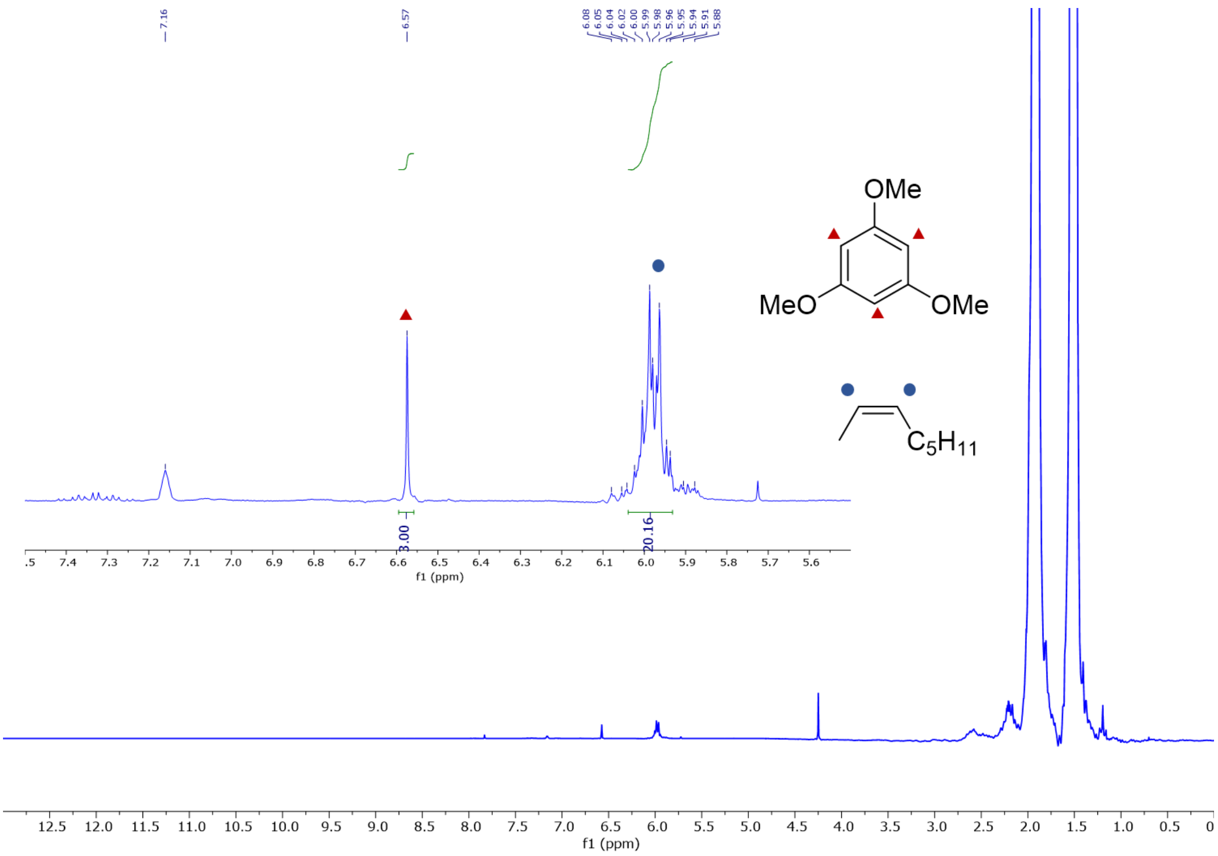

Figure SI 66: ^{1}H NMR spectrum of the hydrogenation of 2-octyne using standard conditions with 1,3,5-trimethoxybenzene (8.4 mg, 0.050 mmol) as internal standard (200 MHz, *n*-hexane with benzene-d_6 capillary). NMR yield: 84%.

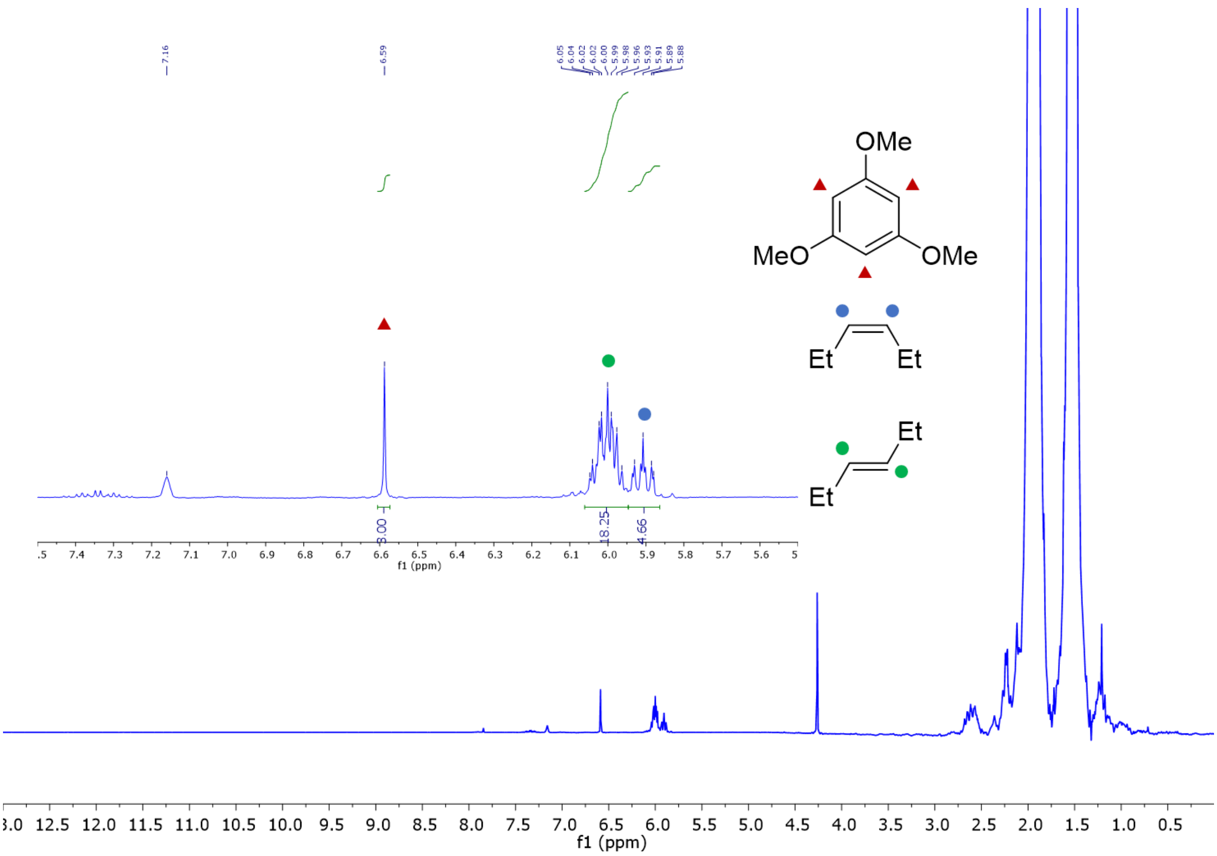

Figure SI 67: ^{1}H NMR spectrum of the hydrogenation of 3-hexyne using standard conditions with 1,3,5-trimethoxybenzene (8.4 mg, 0.050 mmol) as internal standard (200 MHz, *n*-hexane with benzene-d_6 capillary). NMR yield: 95% (*E*/*Z* = 80:20).

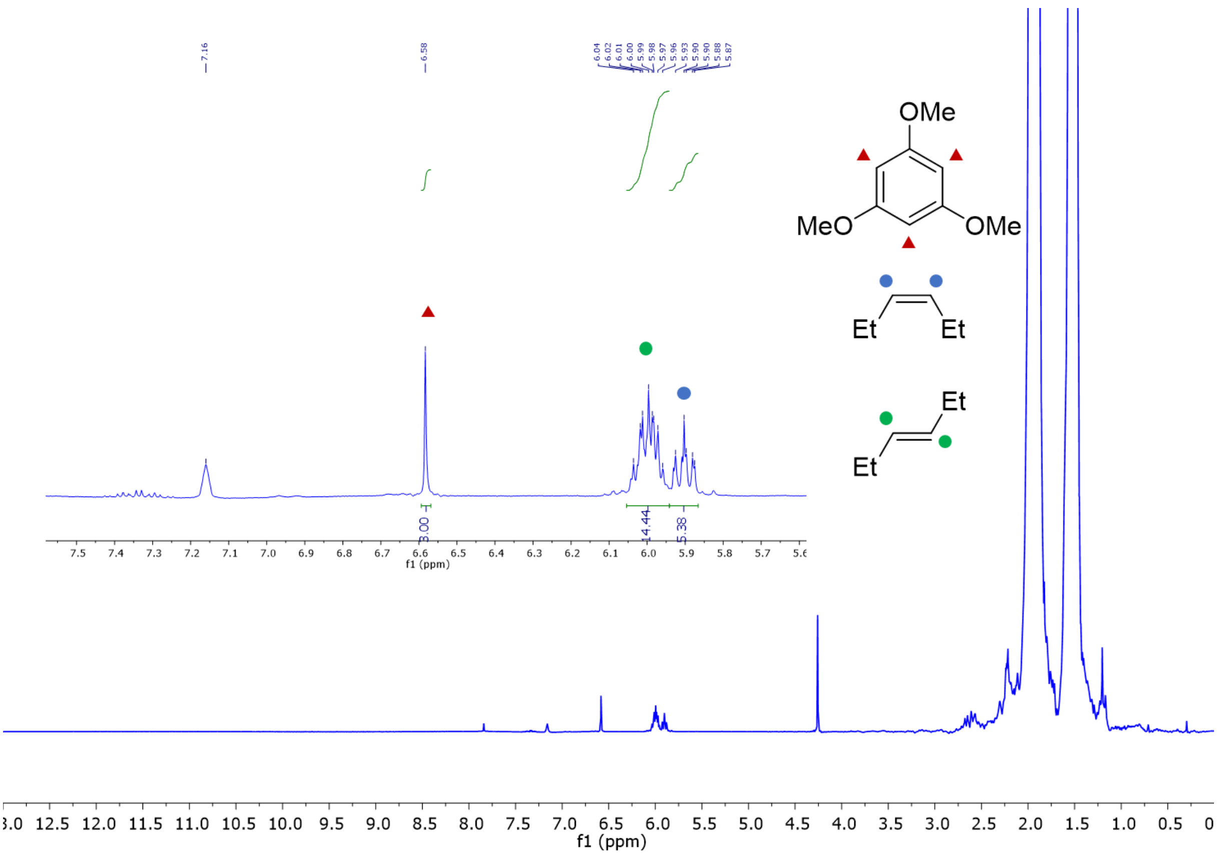

Figure SI 68: ^{1}H NMR spectrum of the hydrogenation of 3-hexyne using standard conditions with 1,3,5-trimethoxybenzene (8.4 mg, 0.050 mmol) as internal standard (200 MHz, *n*-hexane with benzene-d_6 capillary). NMR yield: 83% (*Z*/*E* = 73:27).

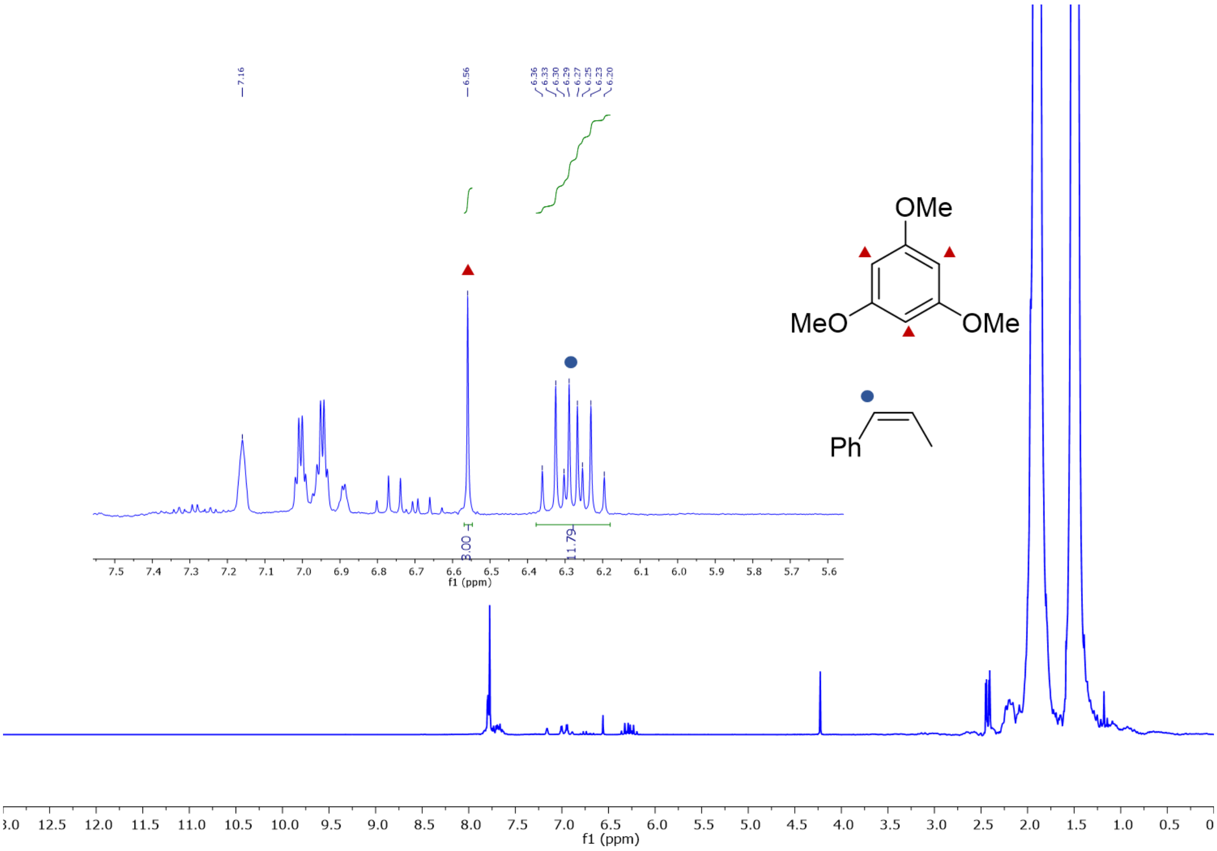

Figure SI 69: ^{1}H NMR spectrum of the hydrogenation of 1-phenyl-2-propyne using standard conditions with 1,3,5-trimethoxybenzene (8.4 mg, 0.050 mmol) as internal standard (200 MHz, *n*-hexane with benzene-d_6 capillary). NMR yield: 98%.

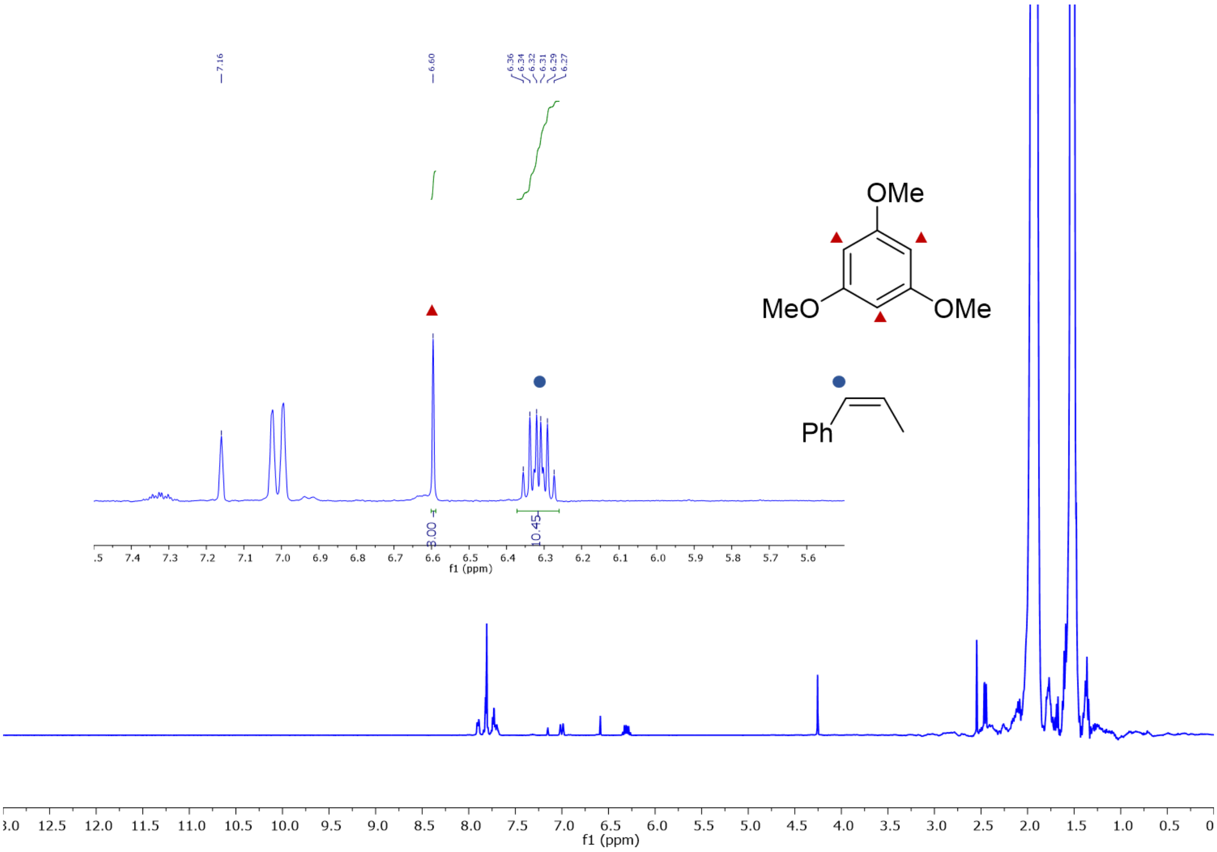

Figure SI 70: ^{1}H NMR spectrum of the hydrogenation of 1-phenyl-2-propyne using standard conditions with 1,3,5-trimethoxybenzene (8.4 mg, 0.050 mmol) as internal standard (200 MHz, *n*-hexane with benzene-d_6 capillary). NMR yield: 87%.

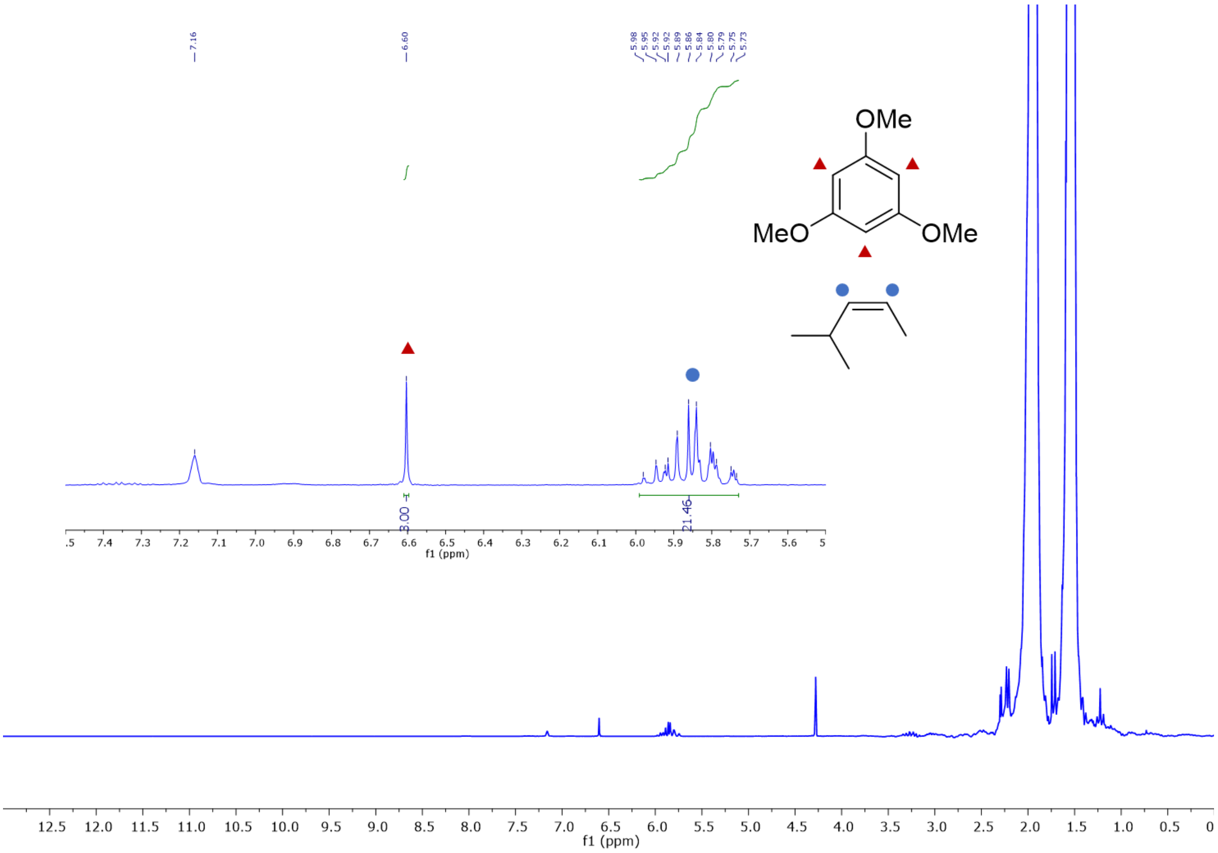

Figure SI 71: ^{1}H NMR spectrum of the hydrogenation of 4-methyl-2-pentyne at 16 h reaction time with 1,3,5-trimethoxybenzene (8.4 mg, 0.050 mmol) as internal standard (200 MHz, *n*-hexane with benzene-d_6 capillary, 5 mol% catalyst). NMR yield: 89%, *Z*-isomer exclusively.

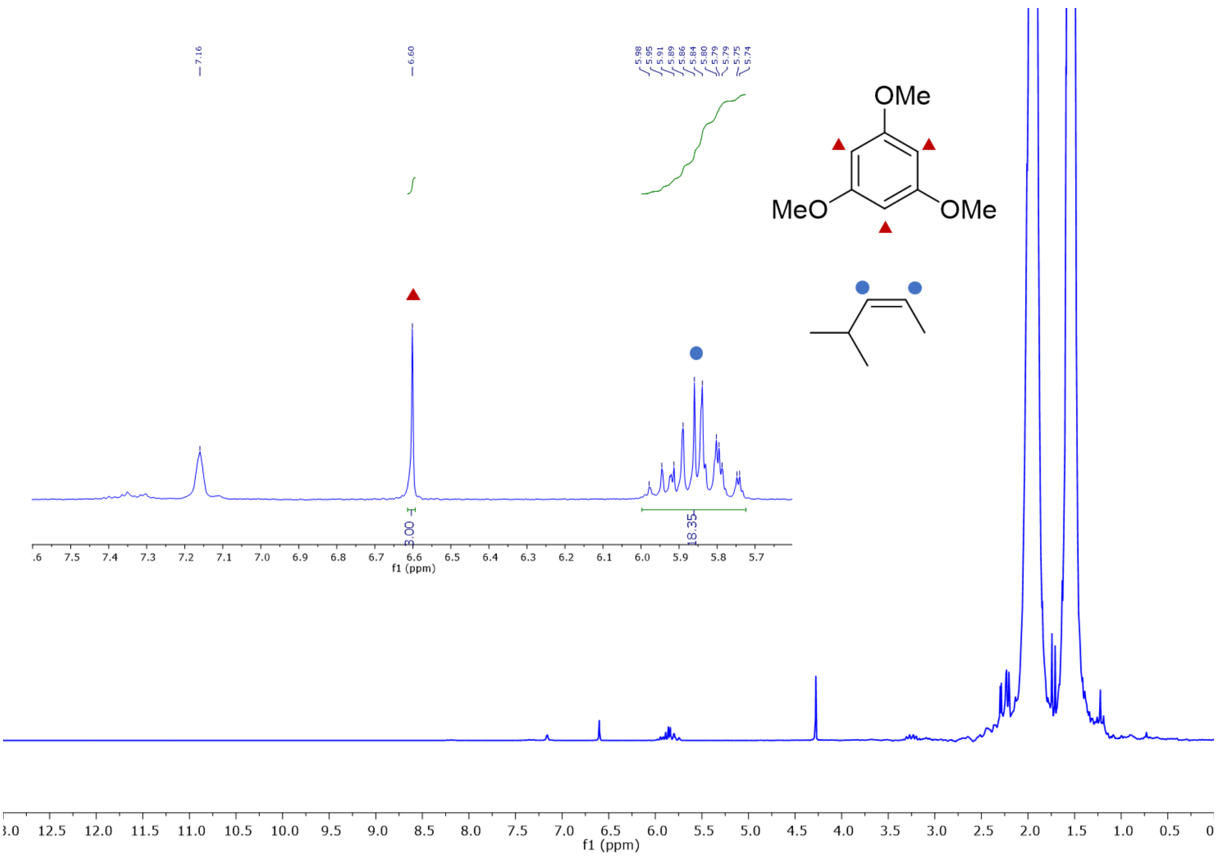

Figure SI 72: ^{1}H NMR spectrum of the hydrogenation of 4-methyl-2-pentyne at 16 h reaction time with 1,3,5-trimethoxybenzene (8.4 mg, 0.050 mmol) as internal standard (200 MHz, *n*-hexane with benzene-d_6 capillary, 5 mol% catalyst). NMR yield: 76%, *Z*-isomer exclusively.

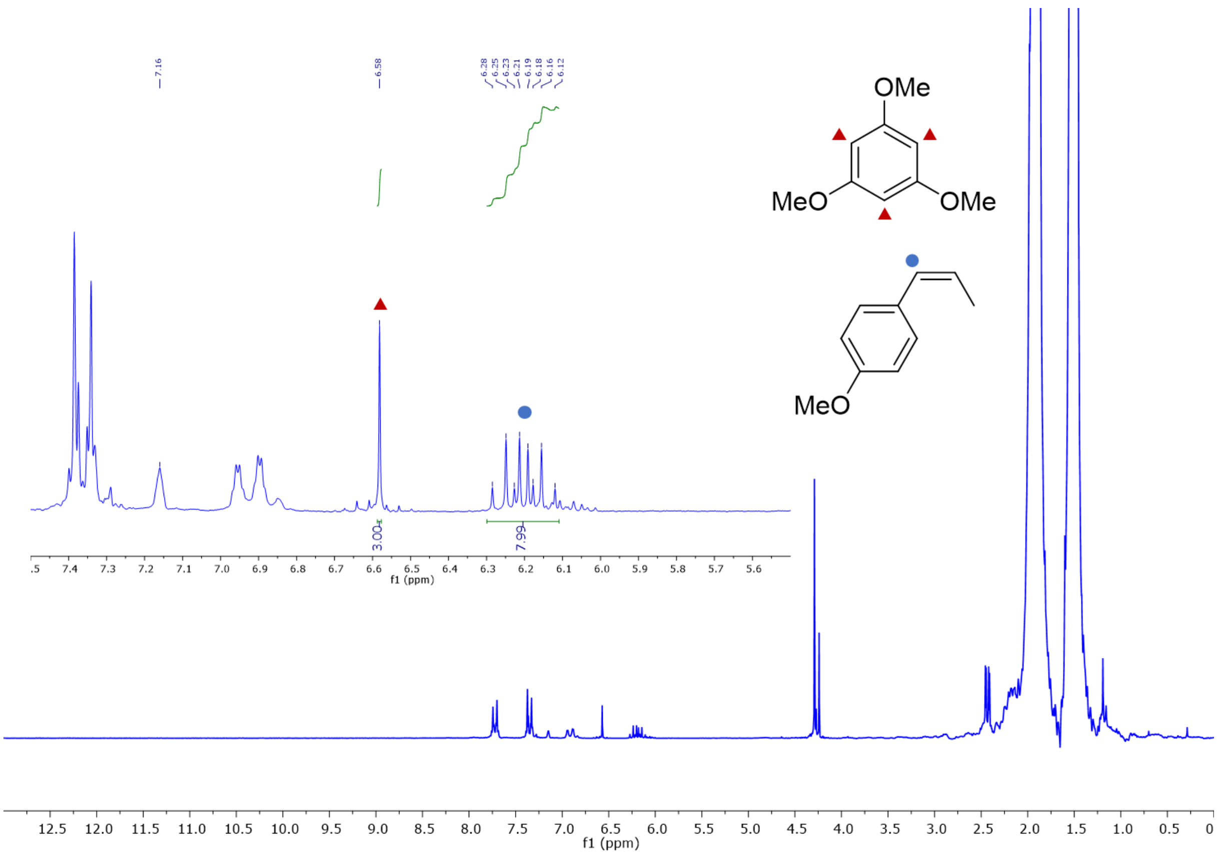

Figure SI 73: ^{1}H NMR spectrum of the hydrogenation of 1-(*para*-methoxyphenyl)propyne at 20 h reaction time with 1,3,5-trimethoxybenzene (8.4 mg, 0.050 mmol) as internal standard (200 MHz, *n*-hexane with benzene-d_6 capillary, 5 mol% catalyst). NMR yield: 67%.

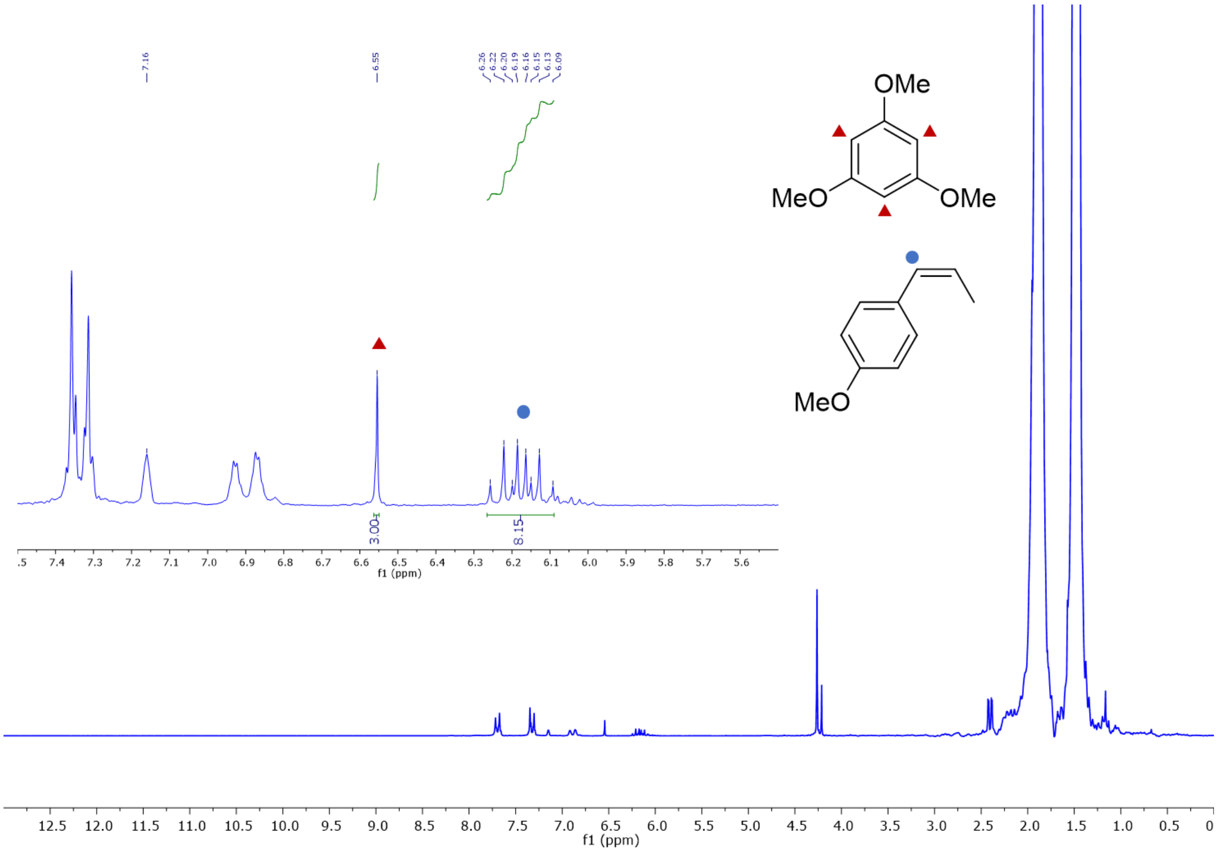

Figure SI 74: ^{1}H NMR spectrum of the hydrogenation of 1-(*para*-methoxyphenyl)propyne at 20 h reaction time with 1,3,5-trimethoxybenzene (8.4 mg, 0.050 mmol) as internal standard (200 MHz, *n*-hexane with benzene-d_6 capillary, 5 mol% catalyst). NMR yield:68 %.

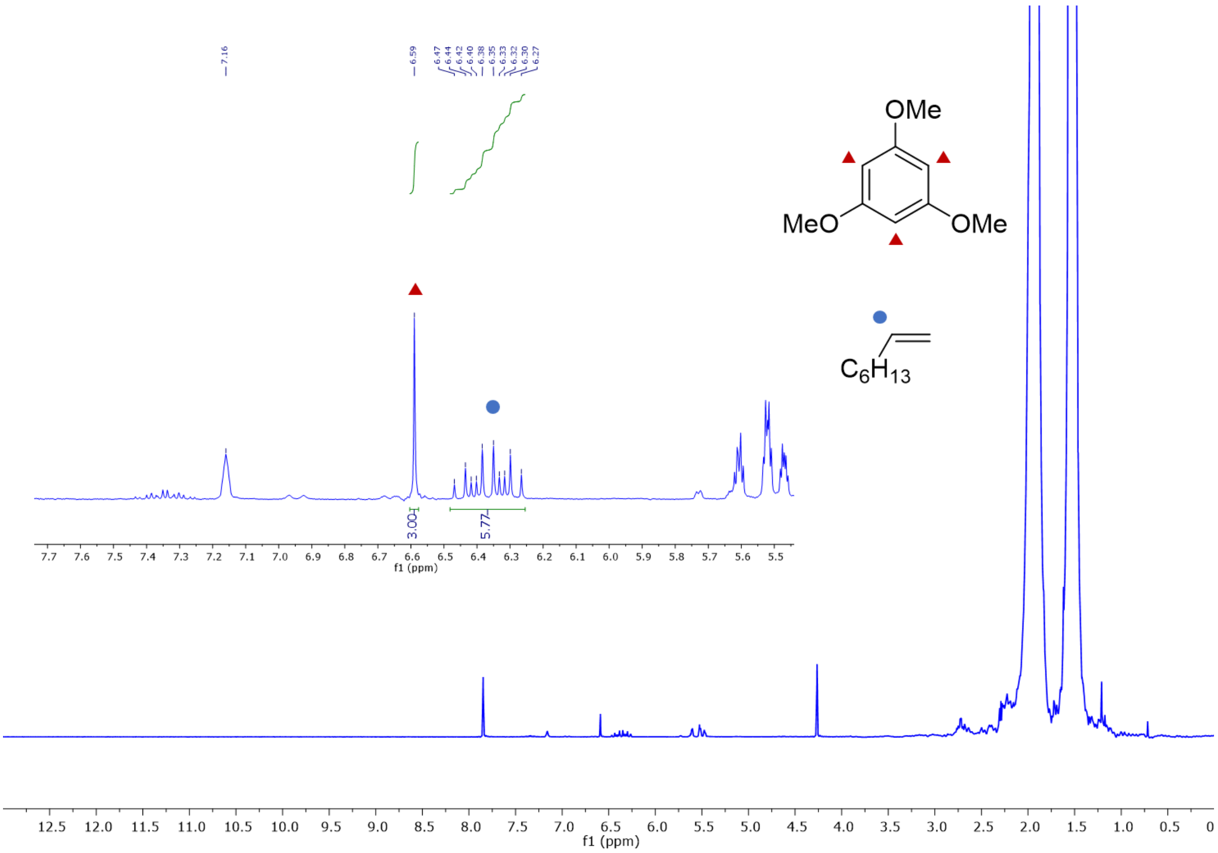

Figure SI 75: ^{1}H NMR spectrum of the hydrogenation of 1-octyne using standard conditions with 1,3,5-trimethoxybenzene (8.4 mg, 0.050 mmol) as internal standard (200 MHz, *n*-hexane with benzene-d_6 capillary). NMR yield: 48%.

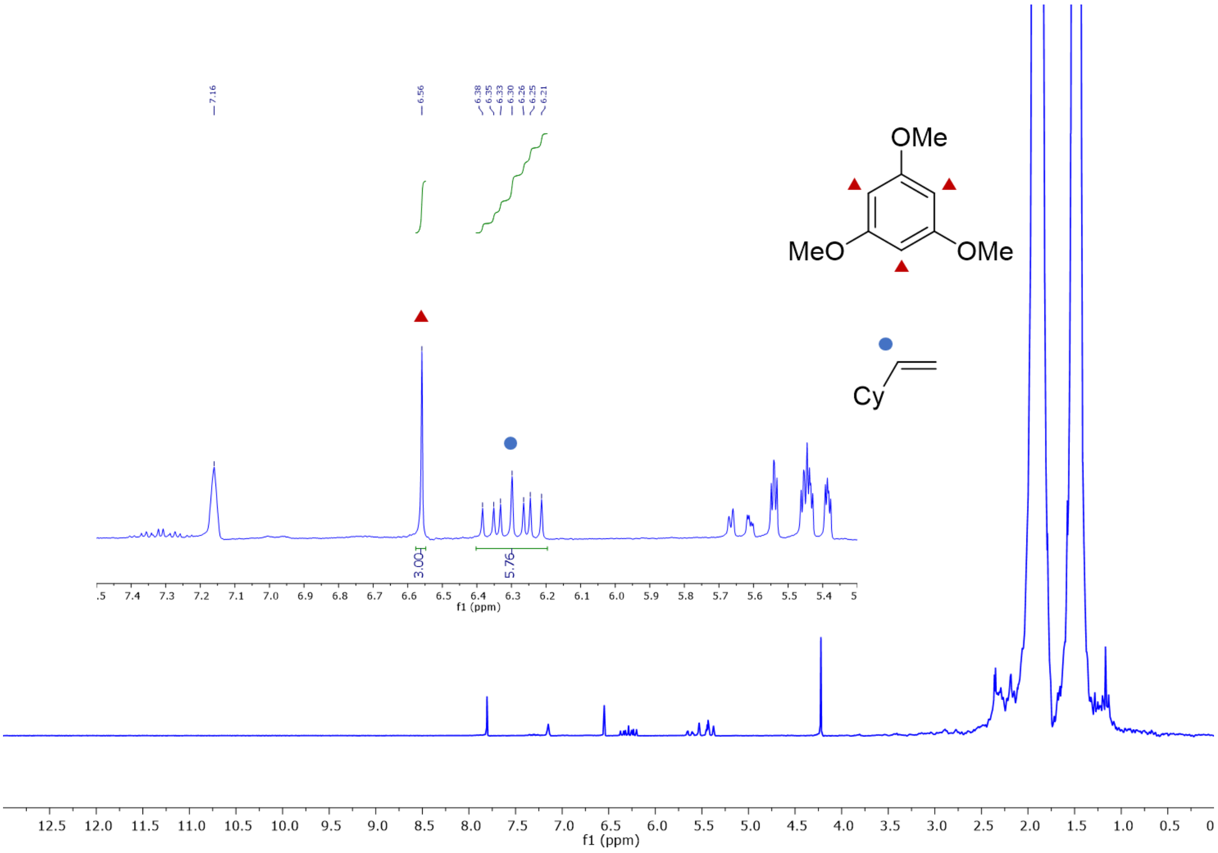

Figure SI 76: ^{1}H NMR spectrum of the hydrogenation of cyclohexylacetylene using standard conditions with 1,3,5-trimethoxybenzene (8.4 mg, 0.050 mmol) as internal standard (200 MHz, *n*-hexane with benzene-d_6 capillary). NMR yield: 48%.

Note: Because of the moderate yields for the hydrogenation of terminal alkynes using standard 5 mol% **5** and 6.5 mol% **6**, the catalyst loading was adapted to 10 mol% **5** and 13 mol% **6** for further experiments. The amount of substrate was therefore reduced from 0.6 mmol to 0.3 mmol.

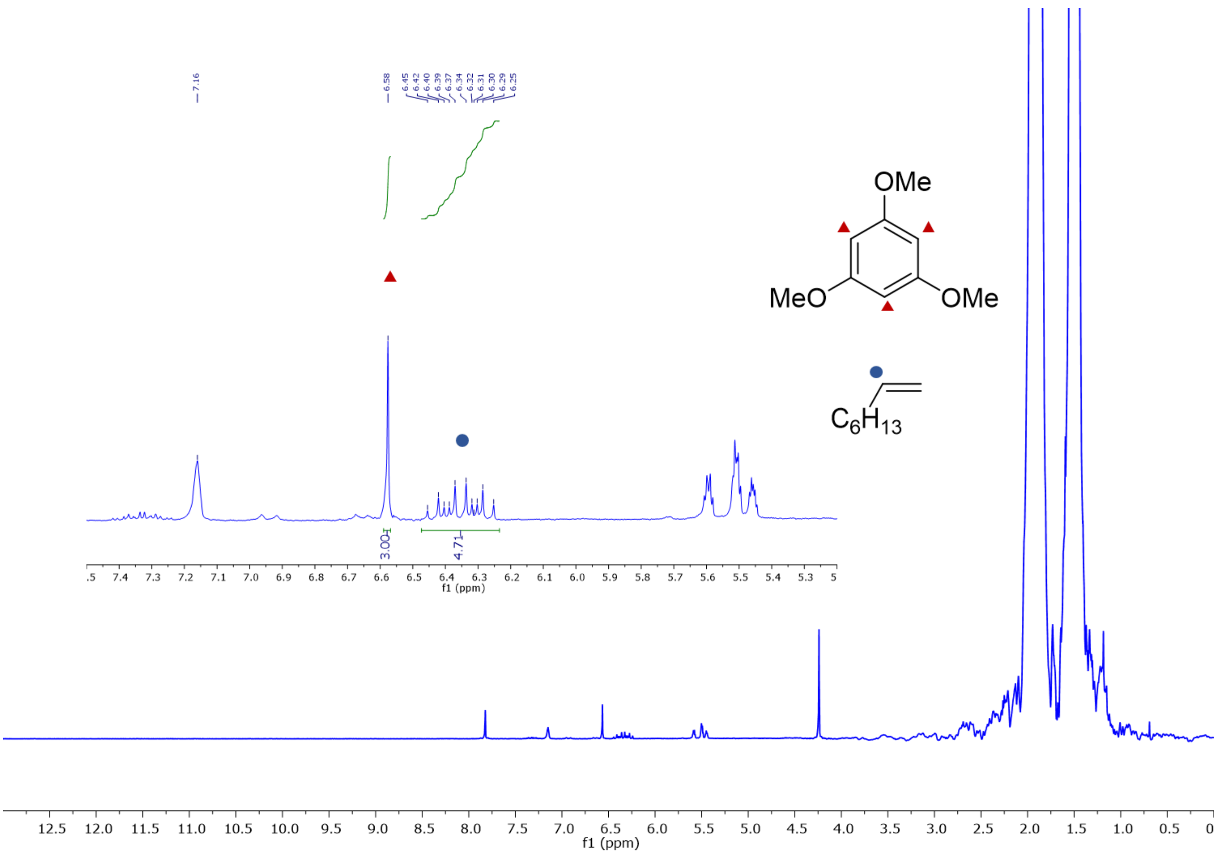

Figure SI 77: ^{1}H NMR spectrum of the hydrogenation of 1-octyne using standard conditions with 1,3,5-trimethoxybenzene (8.4 mg, 0.050 mmol) as internal standard (200 MHz, *n*-hexane with benzene-d_6 capillary, 10 mol% catalyst).

NMR yield: 78%.

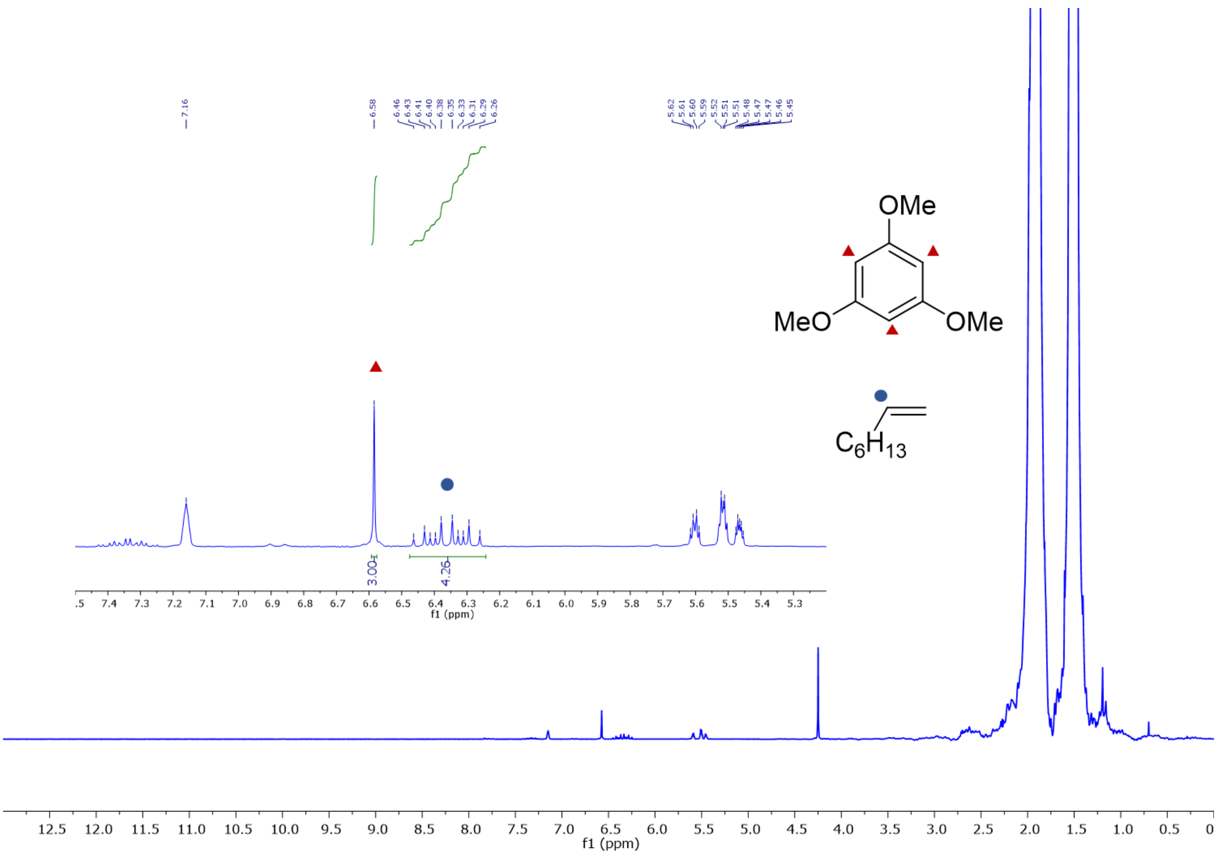

Figure SI 78: ^{1}H NMR spectrum of the hydrogenation of 1-octyne using standard conditions with 1,3,5-trimethoxybenzene (8.4 mg, 0.050 mmol) as internal standard (200 MHz, *n*-hexane with benzene-d_6 capillary). NMR yield: 71%.

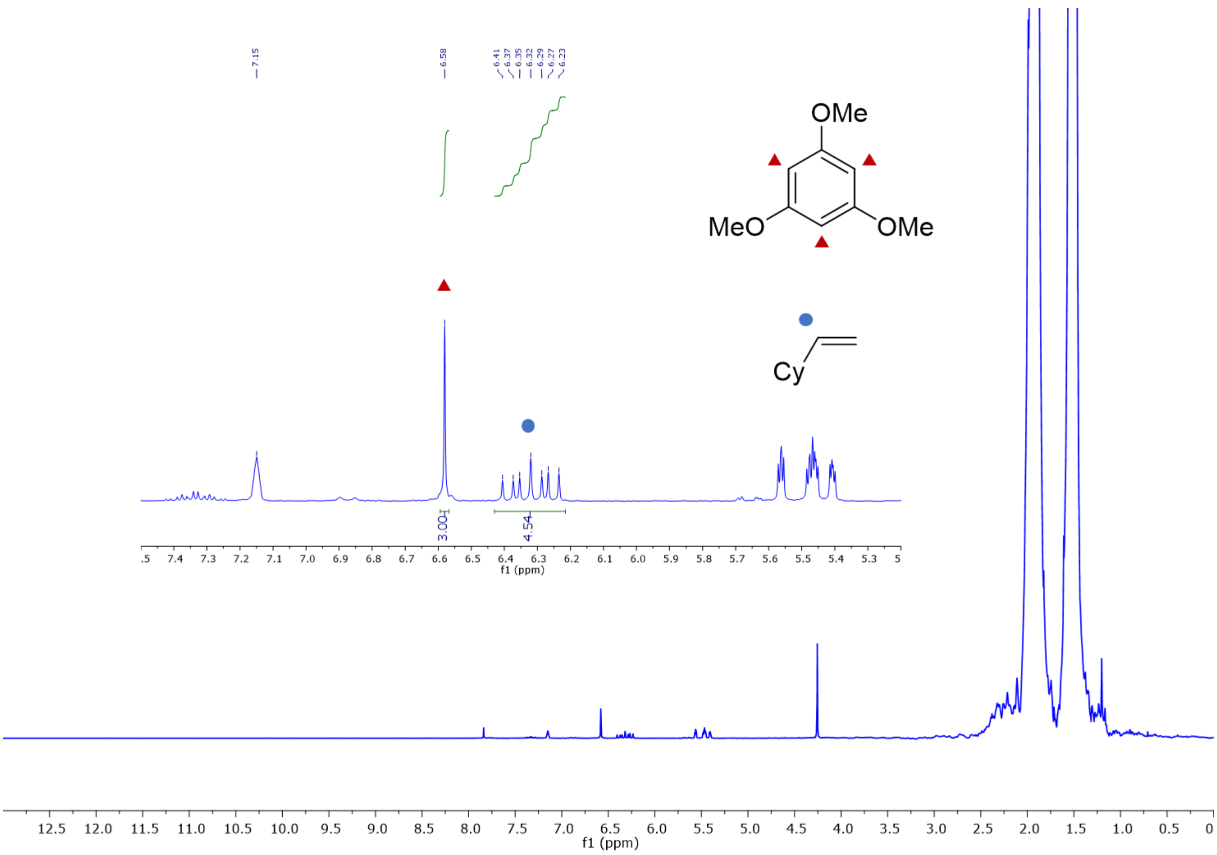

Figure SI 79: ^{1}H NMR spectrum of the hydrogenation of cyclohexyl acetylene using standard conditions with 1,3,5-trimethoxybenzene (8.4 mg, 0.050 mmol) as internal standard (200 MHz, *n*-hexane with benzene-d_6 capillary). NMR yield: 76%.

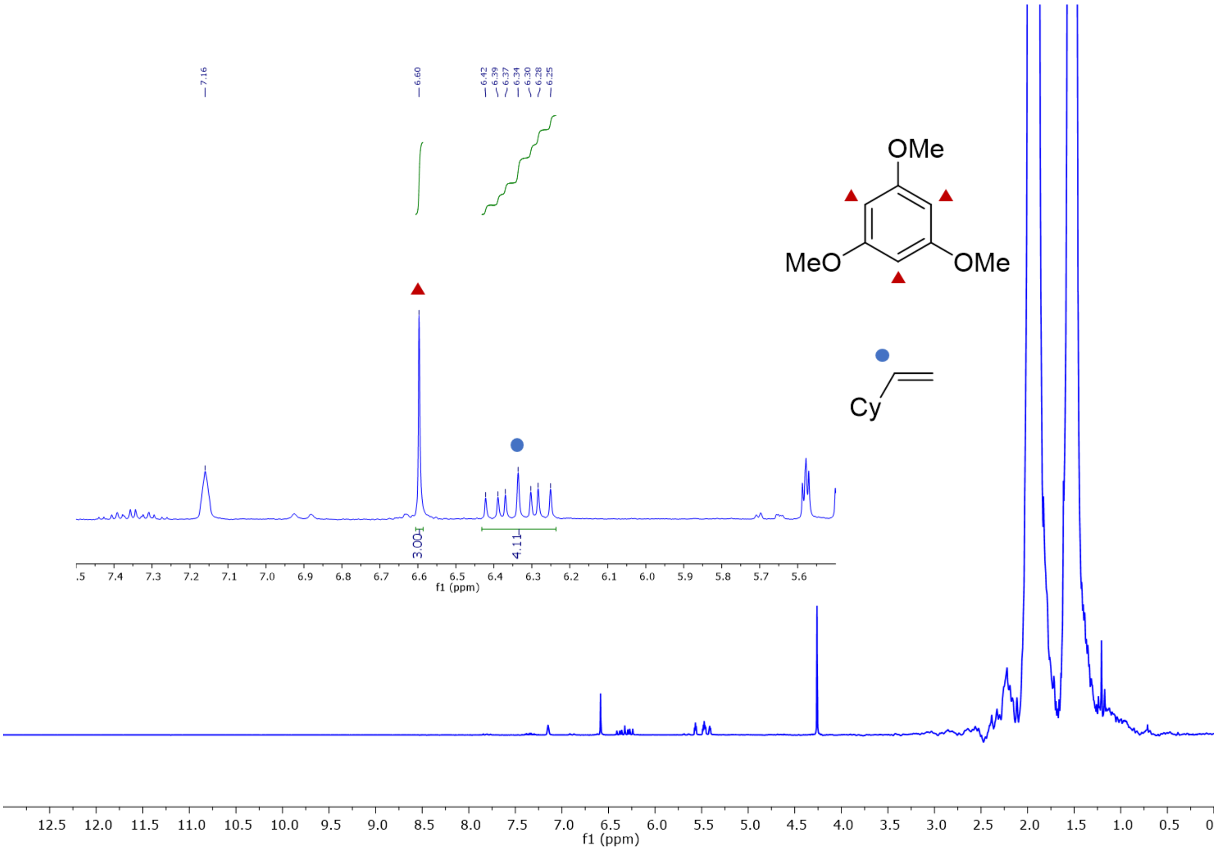

Figure SI 80: ^{1}H NMR spectrum of the hydrogenation of cyclohexyl acetylene using standard conditions with 1,3,5-trimethoxybenzene (8.4 mg, 0.050 mmol) as internal standard (200 MHz, *n*-hexane with benzene-d_6 capillary). NMR yield: 66%.

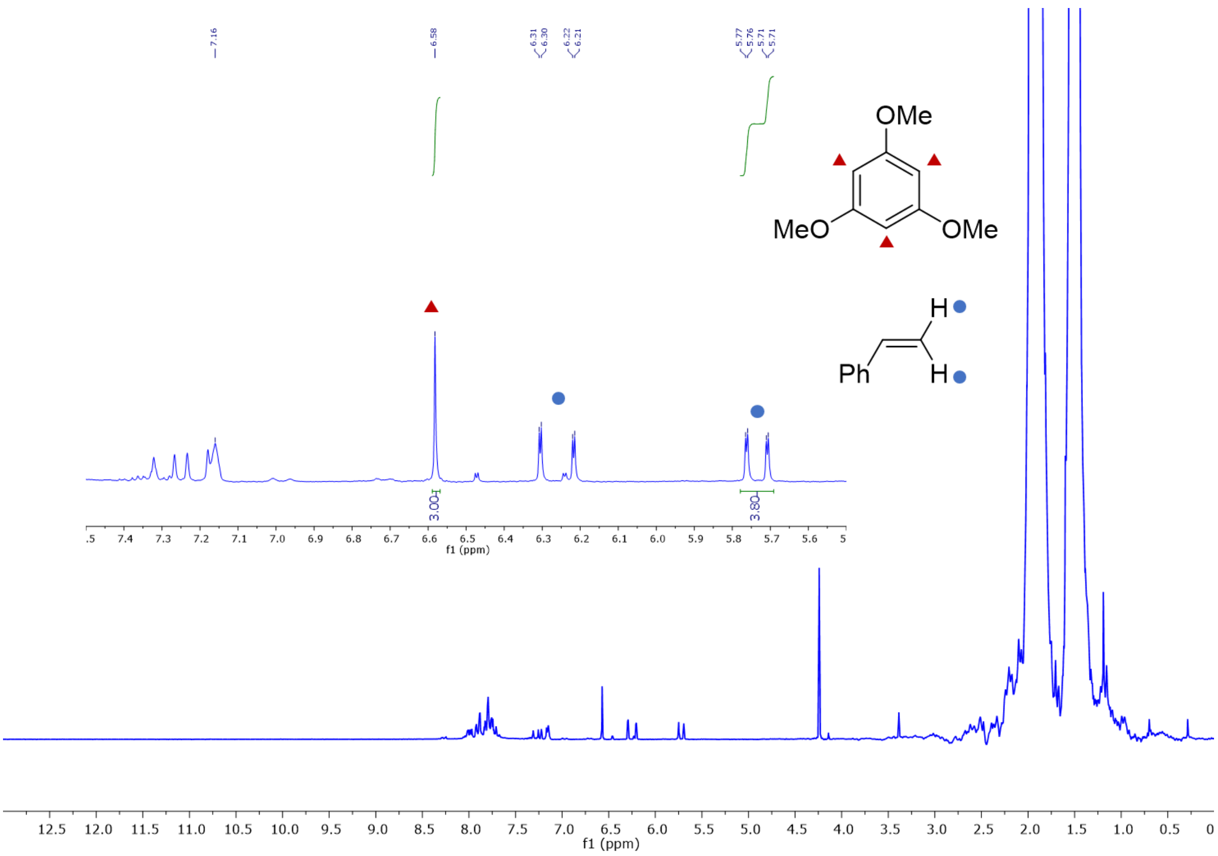

Figure SI 81: ^{1}H NMR spectrum of the hydrogenation of phenylacetylene using standard conditions with 1,3,5-trimethoxybenzene (8.4 mg, 0.050 mmol) as internal standard (200 MHz, *n*-hexane with benzene-d_6 capillary). NMR yield: 63%.

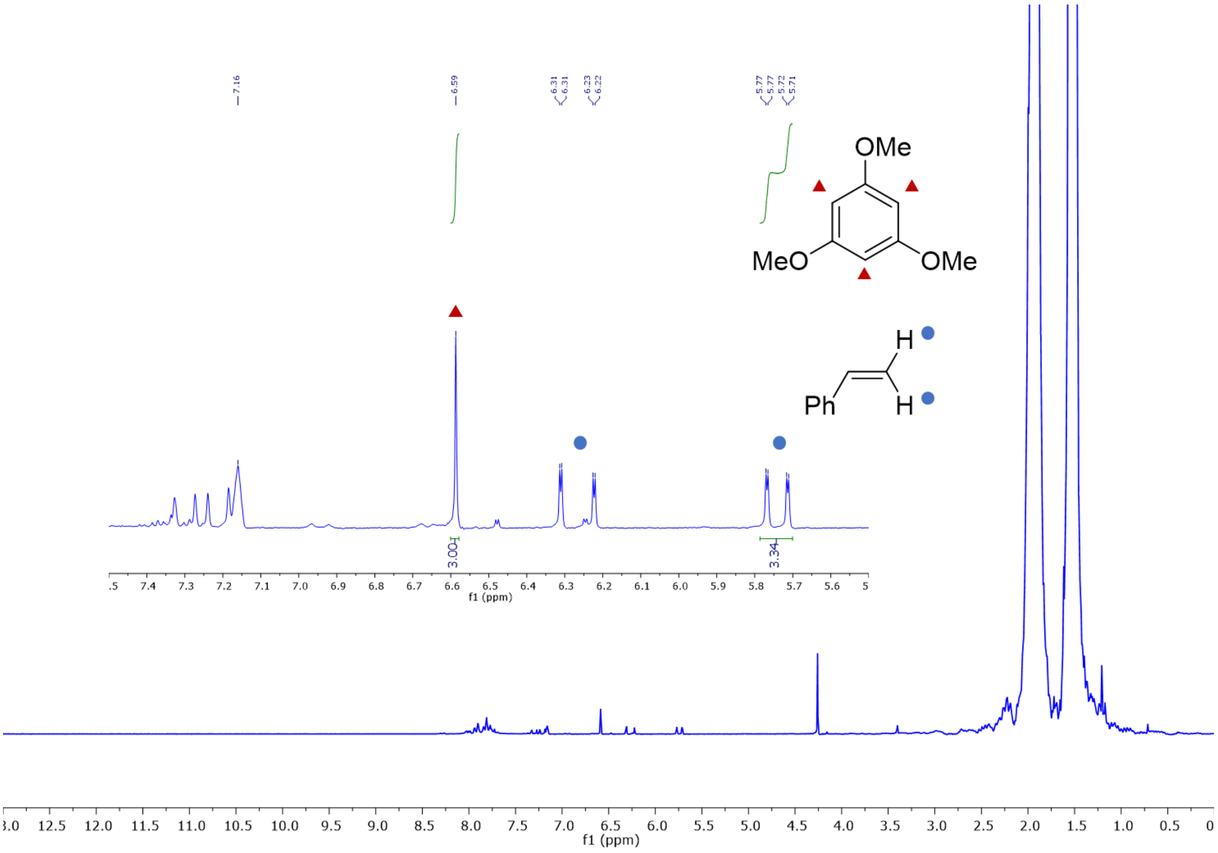

Figure SI 82: ^{1}H NMR spectrum of the hydrogenation of phenylacetylene using standard conditions with 1,3,5-trimethoxybenzene (8.4 mg, 0.050 mmol) as internal standard (200 MHz, *n*-hexane with benzene-d_6 capillary). NMR yield: 56%.

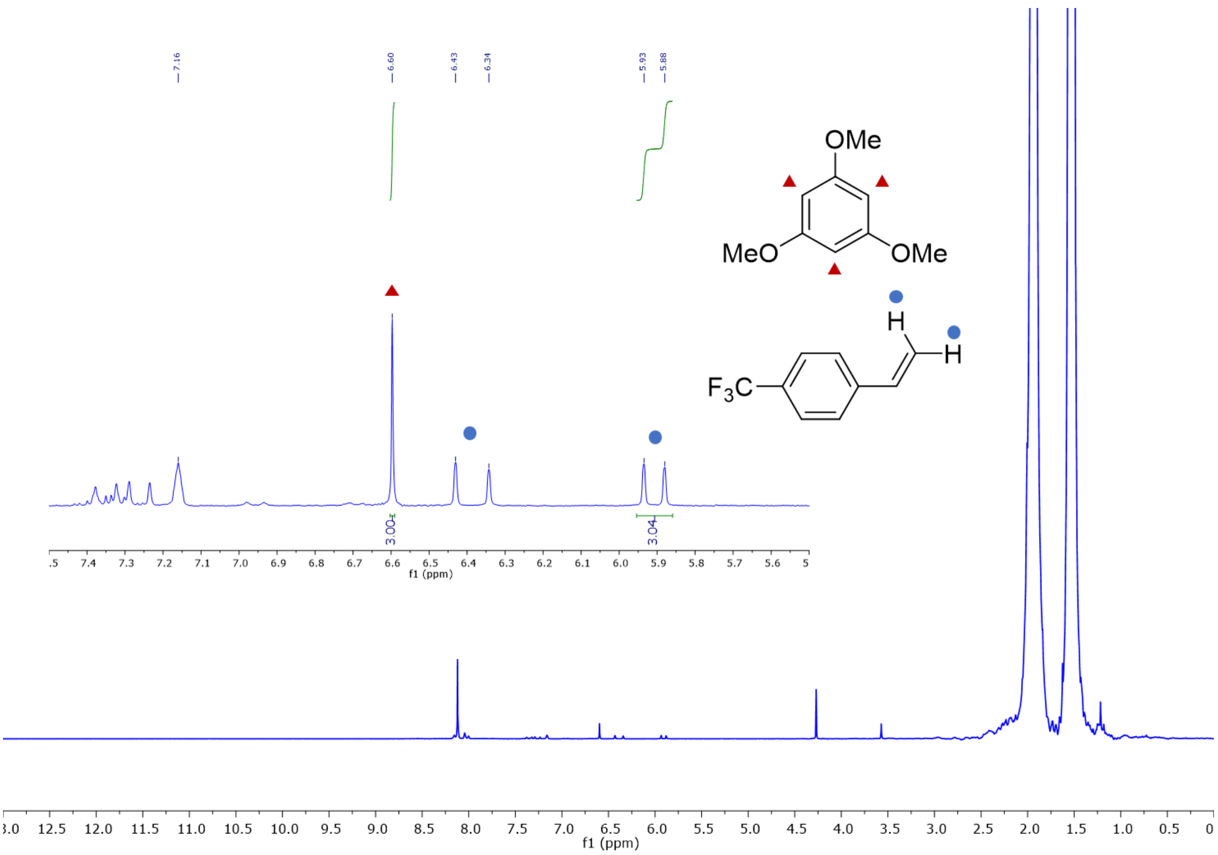

Figure SI 83: ^{1}H NMR spectrum of the hydrogenation of phenylacetylene using standard conditions with 1,3,5-trimethoxybenzene (8.4 mg, 0.050 mmol) as internal standard (200 MHz, *n*-hexane with benzene-d_6 capillary). NMR yield: 50%.

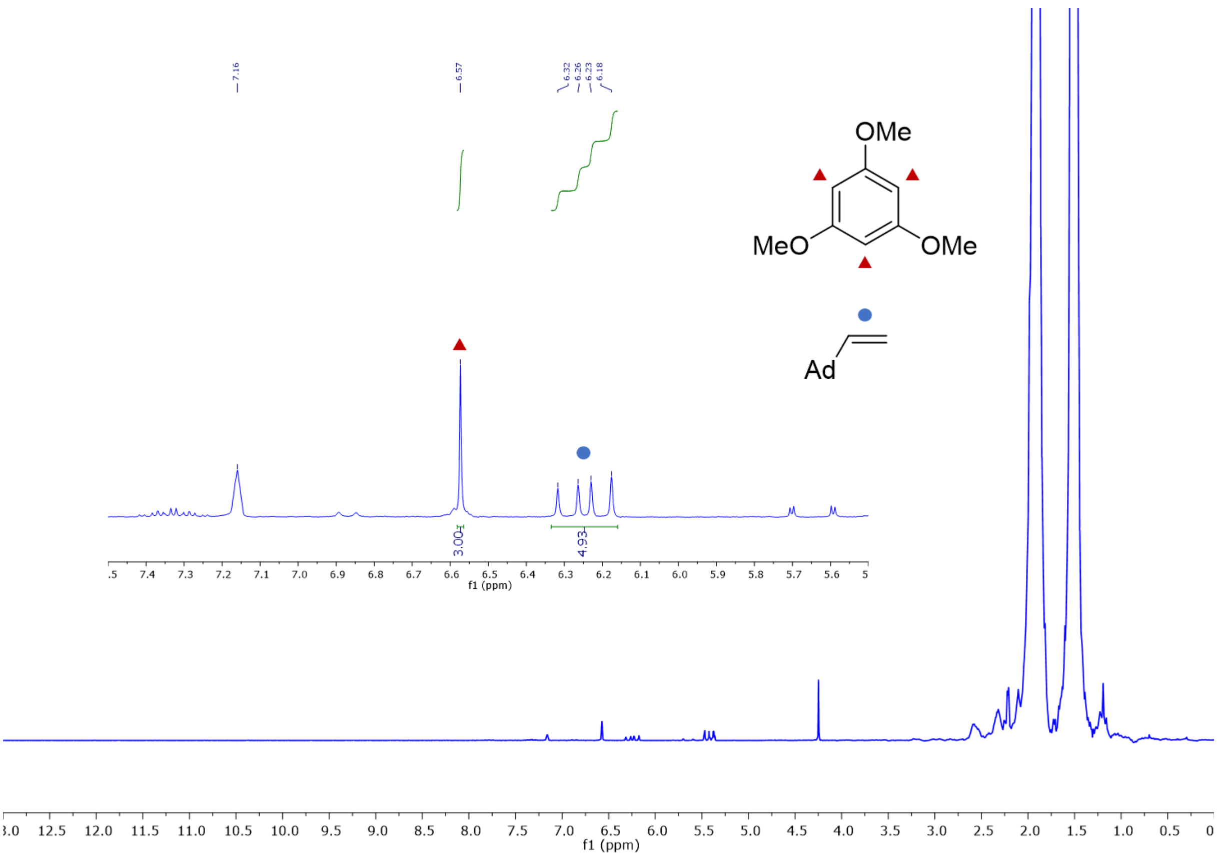

Figure SI 84: ^{1}H NMR spectrum of the hydrogenation of adamantyl acetylene with 1,3,5-trimethoxybenzene (8.4 mg, 0.050 mmol) as internal standard (200 MHz, *n*-hexane with benzene-d_6 capillary). NMR yield: 82%.

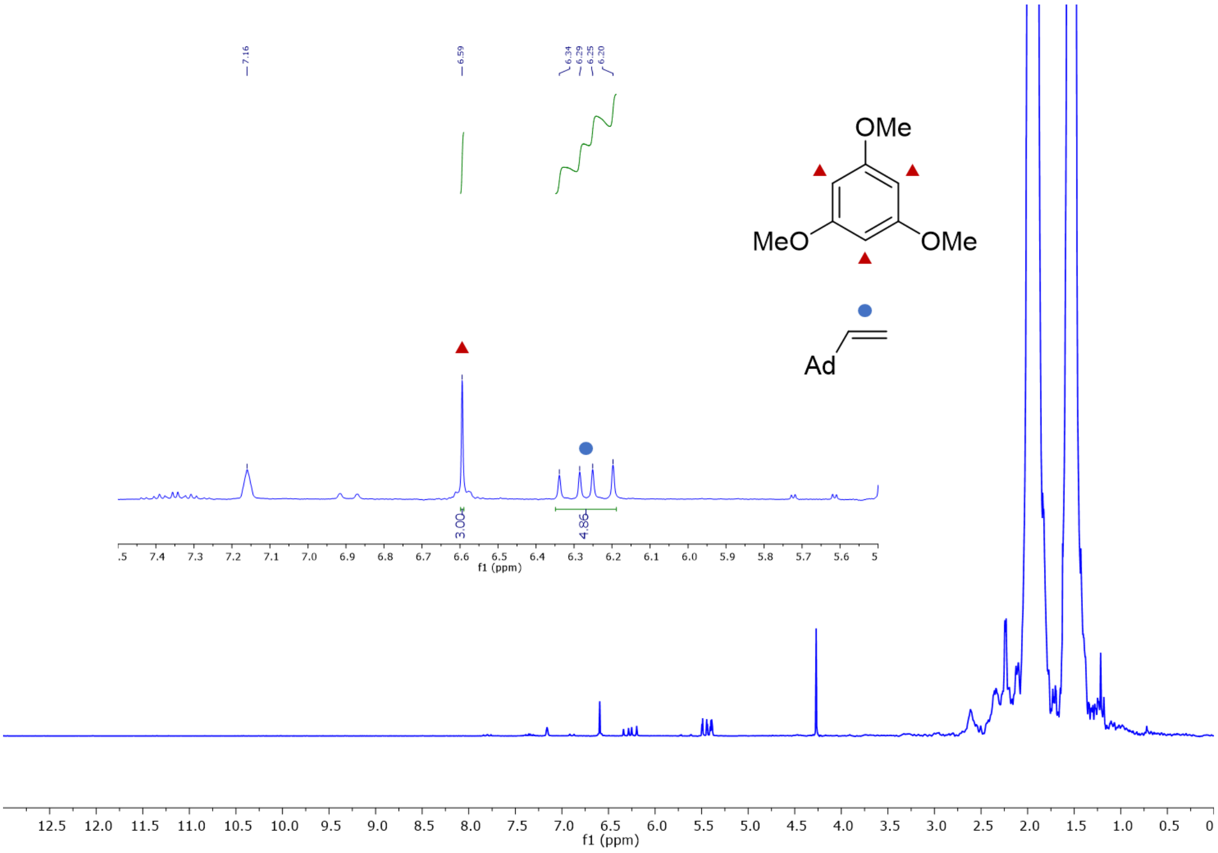

Figure SI 85: ^{1}H NMR spectrum of the hydrogenation of adamantyl acetylene using standard conditions with 1,3,5 trimethoxybenzene (8.4 mg, 0.050 mmol) as internal standard (200 MHz, *n*-hexane with benzene-d_6 capillary). NMR yield: 81%.

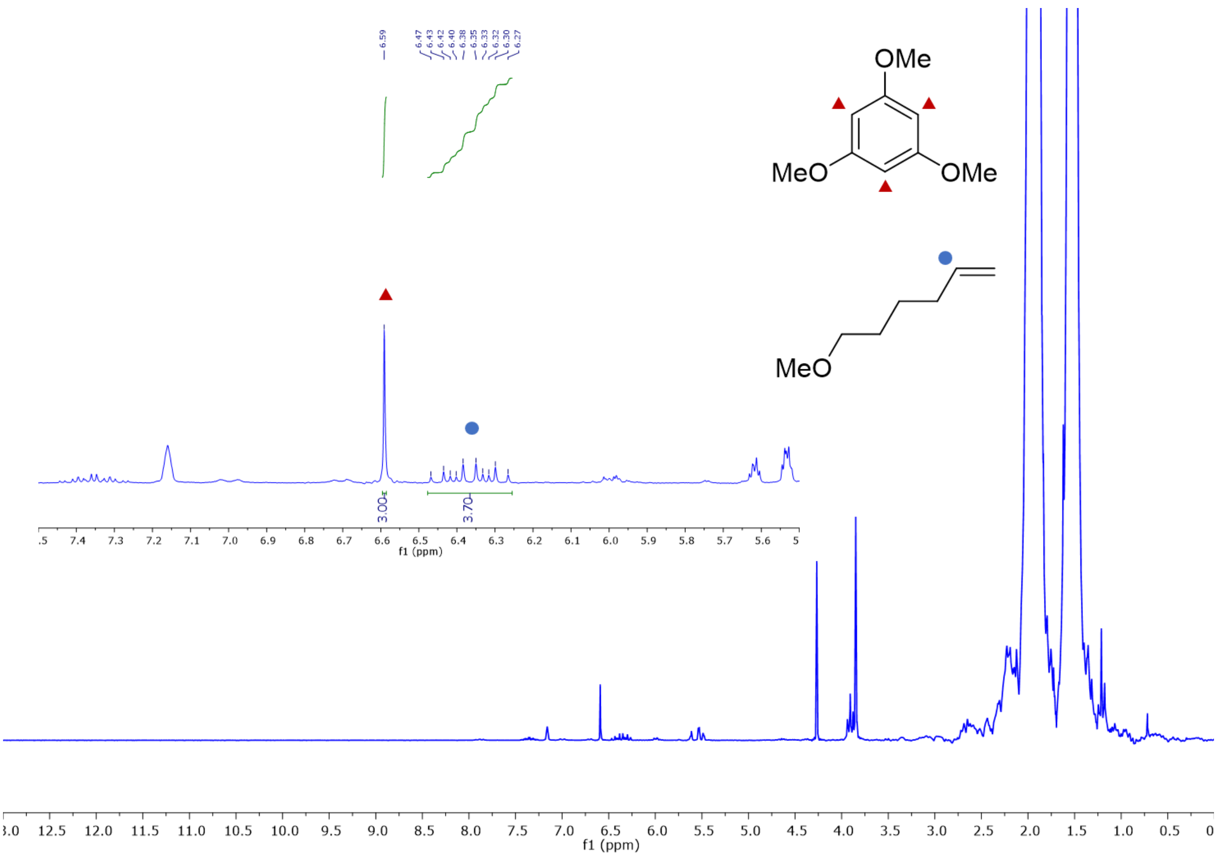

Figure SI 86: ^{1}H NMR spectrum of the hydrogenation of 6-Methoxy-1-hexyne using standard conditions with 1,3,5-trimethoxybenzene (8.4 mg, 0.050 mmol) as internal standard (200 MHz, *n*-hexane with benzene-d_6 capillary). NMR yield: 62%.

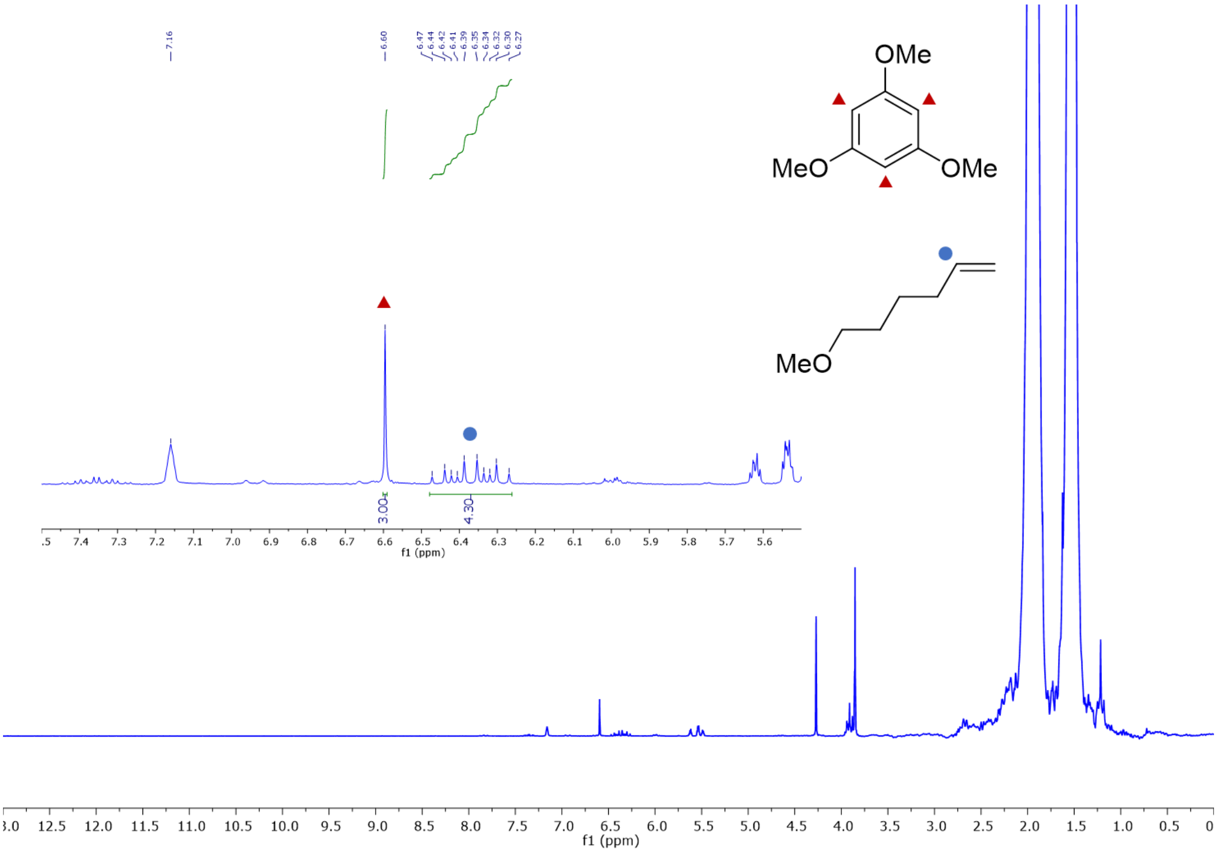

Figure SI 87: ^{1}H NMR spectrum of the hydrogenation of 6-Methoxy-1-hexyne using standard conditions with 1,3,5-trimethoxybenzene (8.4 mg, 0.050 mmol) as internal standard (200 MHz, *n*-hexane with benzene-d_6 capillary). NMR yield: 72%.

5 Tuning the E/Z by changing the reaction time

The isomerisation of alkenes by Piers Borane **6** was described by Liu *et al.* [8] Under standard reaction conditions, we also observed the formation *E*-alkene preferentially for hex-3-yne and a mixture of isomers for 4-methylpent-2-yne. Therefore, the reaction time of the hydrogenation of hex-3-yne and 4-methylpent-2-yne was shortened to 16 h.

While 4-methyl-2-pentyne yielded the *Z*-isomer exclusively as the hydrogenation product, 3-hexyne yielded an *E*/*Z* ratio of 51:49, using standard conditions and 16 h reaction time. Therefore, the reaction time was further shortened to 8 h yielding the *Z*-isomer exclusively as well, while maintaining the excellent NMR yield.

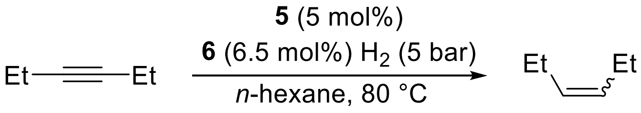

reaction time / h	*Z* / *E*
8	100:0
16	49:51
20	23:77

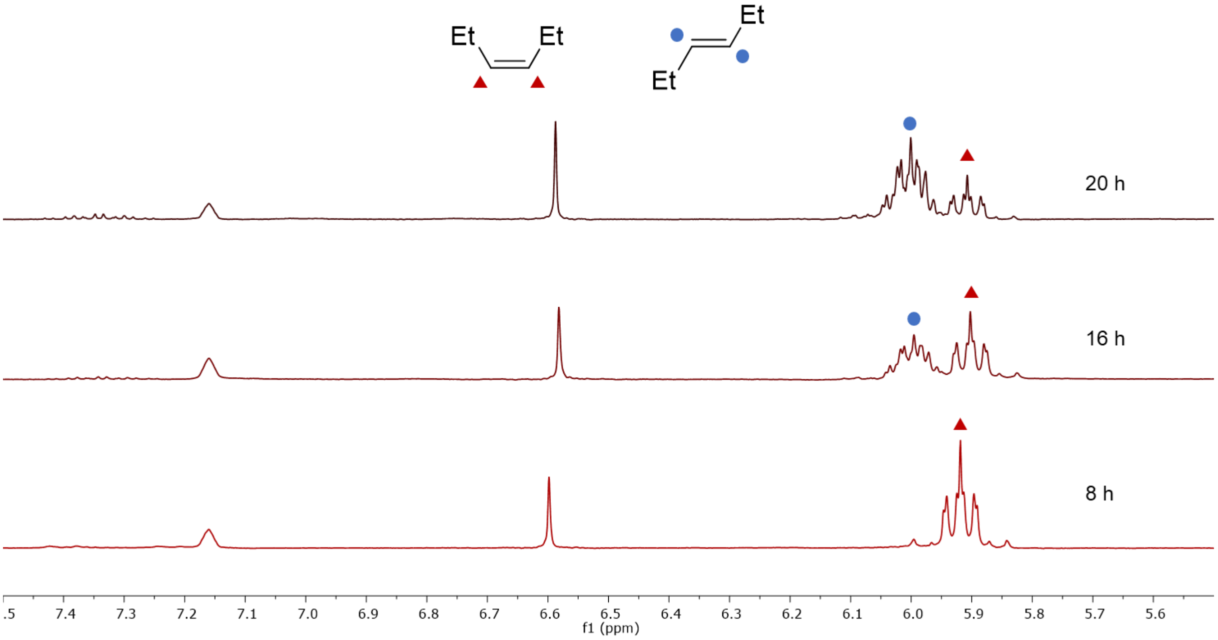

Figure SI 88: Excerpt of the ^{1}H NMR spectra of the hydrogenation of 3-hexyne using standard conditions and different reaction times. Isomerization from *Z* to *E* was observed over time.

While subsequent isomerization leads to the *E*-isomer being formed exclusively for 3-hexyne, no such trend was observed for 2-hexyne and 2-octyne.

5.1 Additional NMR spectra

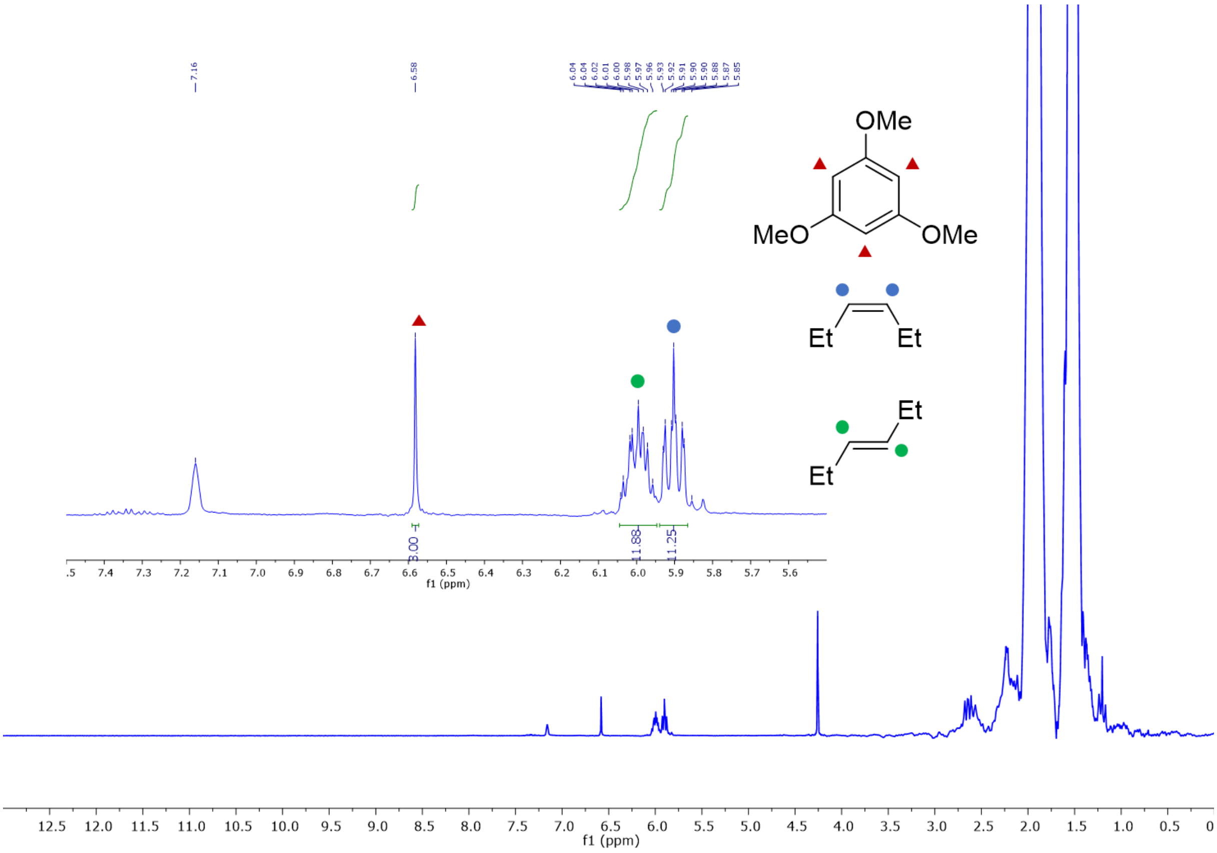

Figure SI 89: ^{1}H NMR spectrum of the hydrogenation of 3-hexyne at 16 h reaction time with 1,3,5-trimethoxybenzene (8.4 mg, 0.050 mmol) as internal standard (200 MHz, *n*-hexane with benzene-d_6 capillary, 5 mol% catalyst). NMR yield: 96%, *E*/*Z*=51:49.

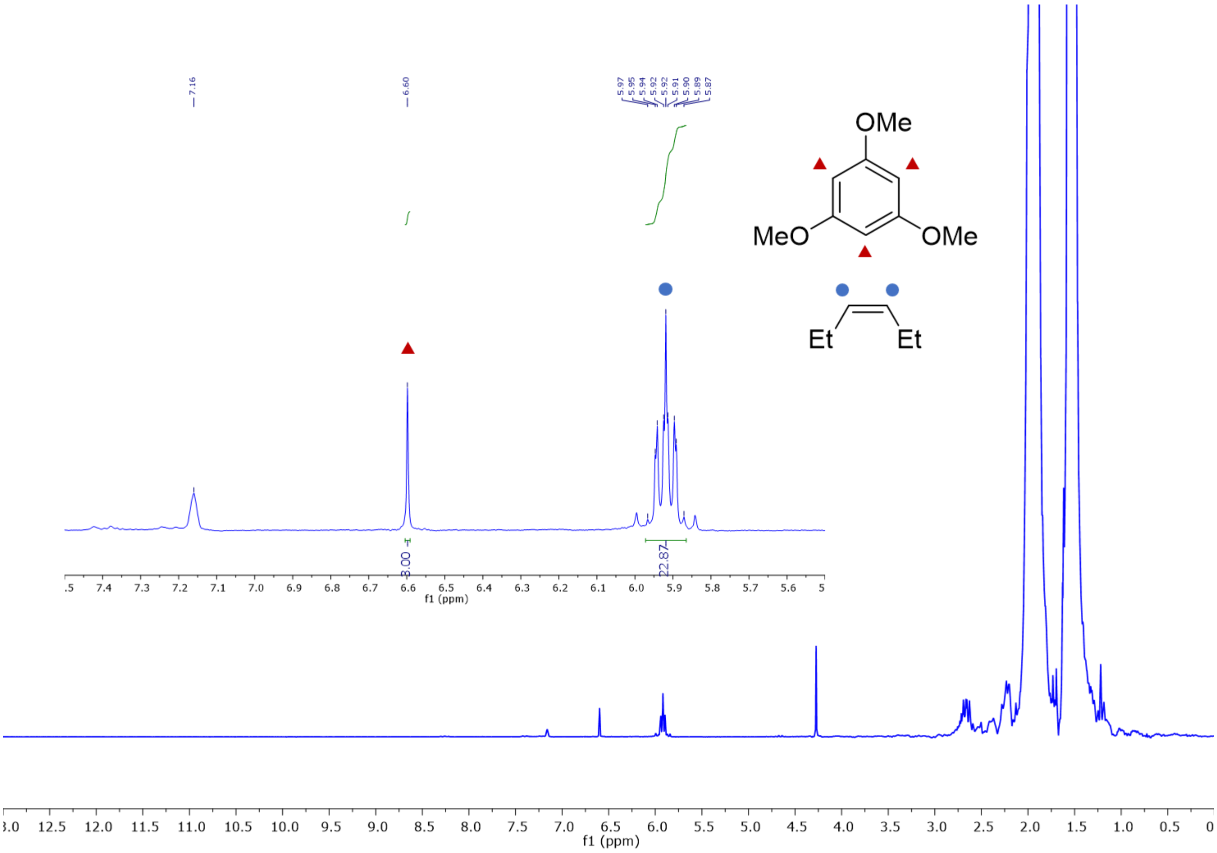

Figure SI 90: ^{1}H NMR spectrum of the hydrogenation of 3-hexyne at 8 h reaction time with 1,3,5-trimethoxybenzene (8.4 mg, 0.050 mmol) as internal standard (200 MHz, *n*-hexane with benzene-d_6 capillary, 5 mol% catalyst).

NMR yield: 95%, *Z*-isomer exclusively.

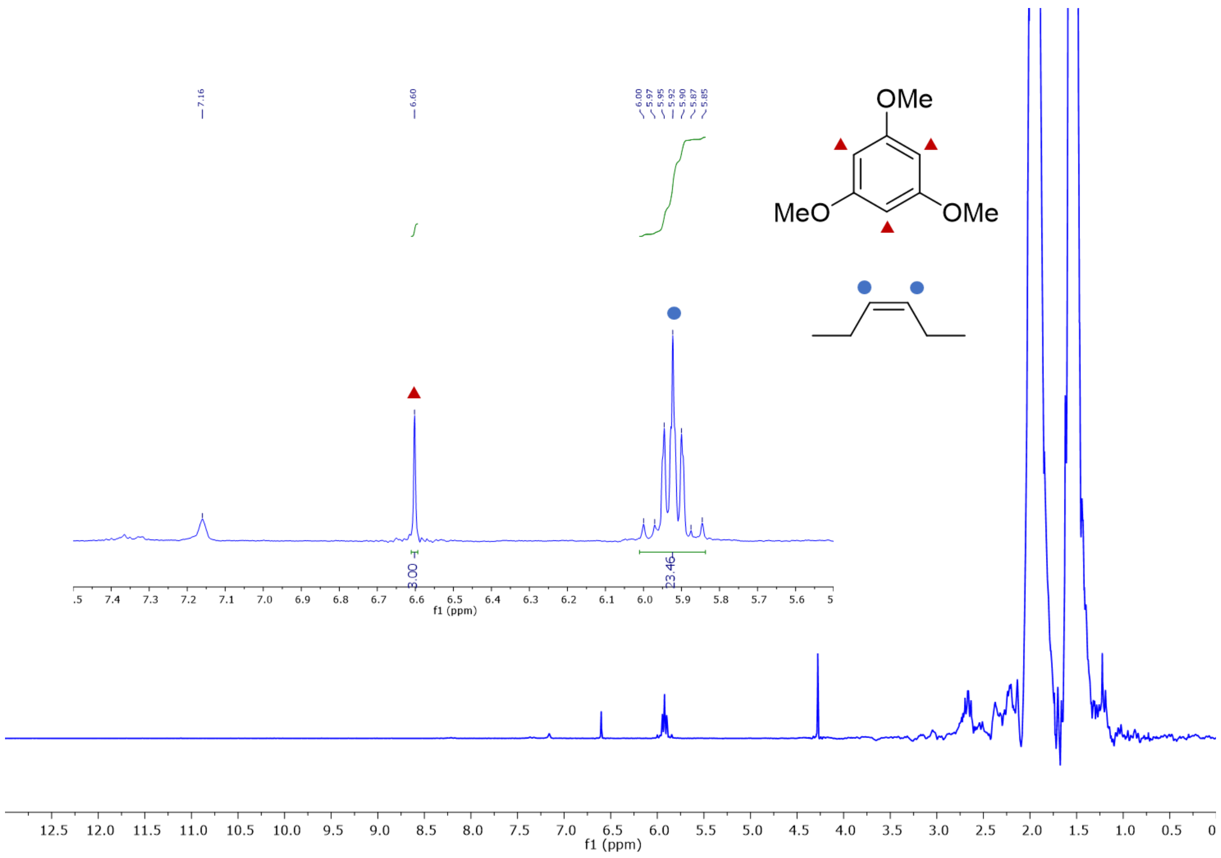

Figure SI 91: ^{1}H NMR spectrum of the hydrogenation of 3-hexyne at 8 h reaction time with 1,3,5-trimethoxybenzene (8.4 mg, 0.050 mmol) as internal standard (200 MHz, *n*-hexane with benzene-d_6 capillary, 5 mol% catalyst). NMR yield: 97%, *Z*-isomer exclusively.

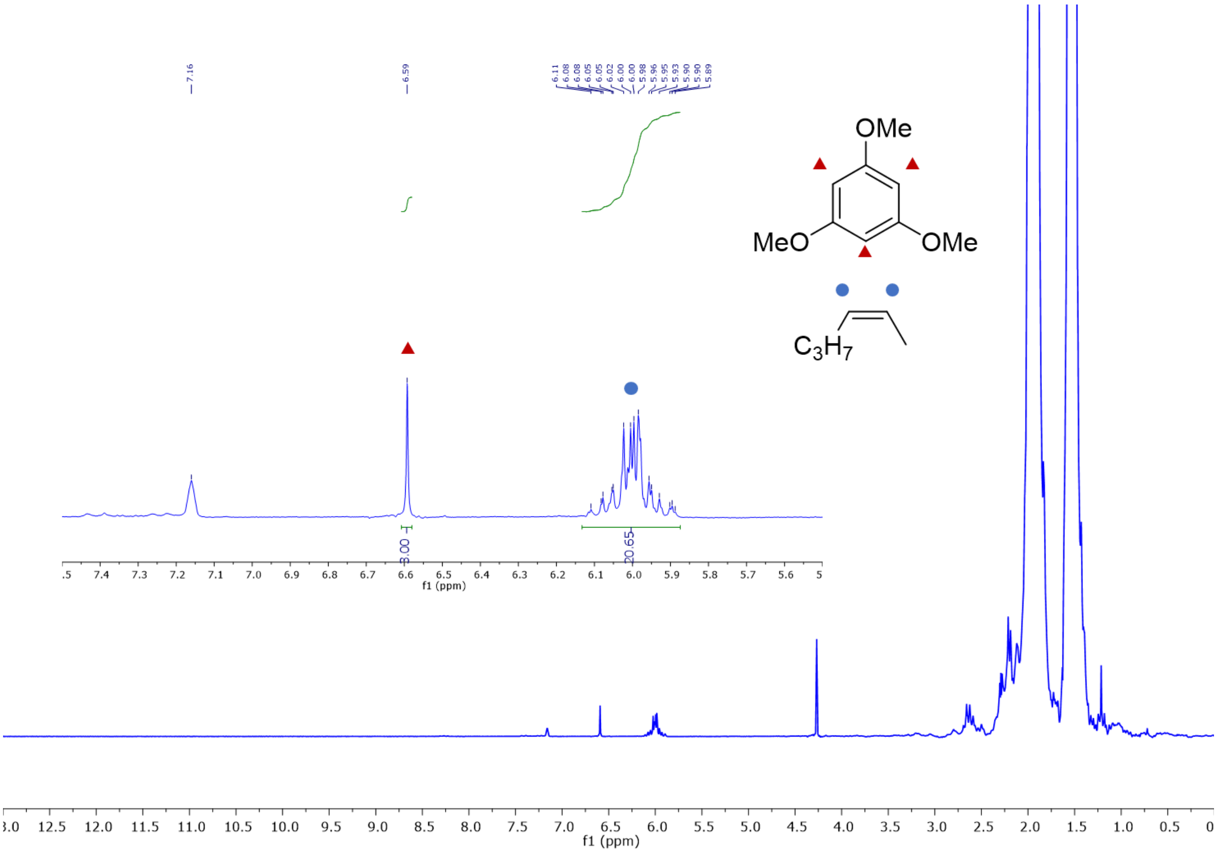

Figure SI 92: ^{1}H NMR spectrum of the hydrogenation of 2-hexyne at 8 h reaction time with 1,3,5-trimethoxybenzene (8.4 mg, 0.050 mmol) as internal standard (200 MHz, *n*-hexane with benzene-d_6 capillary, 5 mol% catalyst).

NMR yield: 86%

6 Computational details

All structures were fully optimized with the composite method PBEh-3c.[9] Solvent effects were considered implicitly using the SMD model for THF.[10] Thermodynamic properties were obtained at the same level of theory from a frequency computation. The computed free energies were corrected regarding the standard state by adding RT ln(c0s/c0g) (i.e., about 1.89 kcal mol-1) to energies of all structures. For final single point energy computations, the revDSD-PBEP86 functional with Grimmes D4 dispersion correction and the def2-QZVPP basis set was used in combination with the RIJK aproximation.[11]

3

C	-2.950310000	-3.070206000	-1.959316000
C	-1.610537000	-2.742968000	-1.979783000
C	-3.756608000	-2.625161000	-0.918699000
C	-1.150860000	-1.964862000	-0.926059000
B	0.691748000	-0.476015000	-0.473638000
C	-0.160002000	0.828191000	-0.277423000
C	-0.051943000	1.577067000	0.880290000
C	-1.025969000	1.295025000	-1.250137000
C	-0.775993000	2.737586000	1.080792000
C	-1.760836000	2.453556000	-1.090787000
C	-1.632207000	3.174484000	0.084630000
C	2.242109000	-0.463900000	-0.244383000
C	2.894262000	-1.506190000	0.400437000
C	3.026642000	0.598429000	-0.668830000
C	4.259321000	-1.497014000	0.620236000
C	4.394831000	0.633645000	-0.478648000
C	5.008587000	-0.422217000	0.173089000
F	-1.150250000	0.620164000	-2.391098000
F	0.757762000	1.162711000	1.852502000
F	-0.662336000	3.429255000	2.206220000
F	-2.330280000	4.284884000	0.256050000
F	-2.579653000	2.881357000	-2.042285000
F	2.463264000	1.617969000	-1.311499000
F	5.120111000	1.655239000	-0.912121000
F	6.315415000	-0.404098000	0.369285000
F	4.855392000	-2.498518000	1.252184000
F	2.201739000	-2.538809000	0.862350000
C	-3.190425000	-1.858626000	0.091020000
H	-4.805423000	-2.880032000	-0.904137000
H	-3.372678000	-3.671208000	-2.753714000
H	-0.947874000	-3.062150000	-2.771187000
N	-1.892307000	-1.551471000	0.066466000
O	0.169323000	-1.644486000	-0.897572000
C	-3.964513000	-1.318042000	1.281437000
C	-3.341656000	-1.878057000	2.565591000
H	-2.290179000	-1.605660000	2.646176000
H	-3.862605000	-1.483797000	3.440067000
H	-3.412081000	-2.966616000	2.599395000
C	-3.846707000	0.210833000	1.284830000
H	-2.805570000	0.522071000	1.358737000
H	-4.270190000	0.646745000	0.377956000
H	-4.383559000	0.630056000	2.137591000
C	-5.441276000	-1.703220000	1.232922000
H	-5.585142000	-2.784970000	1.255385000
H	-5.957067000	-1.292178000	2.101406000
H	-5.940211000	-1.309468000	0.345512000

	Hatree	kcal mol^{-1}
E PBEh-3c	-1956.742336	-1227874.405
G	0.25494324	159.9793051
E SMD PBEh-3c	-1956.75274	-1227880.934

E revDSD-PBEP86-D4/def2-QZVPP -1958.672435 -1229085.561

$TS_{3/4}$

C	-2.944737000	-2.993922000	2.156614000
C	-1.665400000	-2.511553000	2.301177000
C	-3.664758000	-2.767660000	0.982025000
C	-1.144397000	-1.794808000	1.225795000
B	0.523664000	-0.357419000	0.301894000
C	2.101336000	-0.418160000	0.089355000
C	2.938562000	0.640443000	0.402855000
C	2.718073000	-1.571442000	-0.372772000
C	4.312989000	0.573344000	0.253351000
C	4.085679000	-1.673591000	-0.542386000
C	4.885908000	-0.589845000	-0.226017000
C	-0.228399000	1.055597000	0.249901000
C	-0.101613000	1.874316000	-0.862467000
C	-1.028517000	1.539964000	1.270978000
C	-0.734314000	3.096124000	-0.978192000
C	-1.680092000	2.759880000	1.193744000
C	-1.531948000	3.539332000	0.062678000
F	1.979246000	-2.634683000	-0.687586000
F	2.436965000	1.771768000	0.893397000
F	5.082288000	1.606803000	0.570934000
F	6.198405000	-0.669368000	-0.377547000
F	4.635802000	-2.791153000	-1.000367000
F	-1.199664000	0.838490000	2.389943000
F	-2.442285000	3.185210000	2.193802000
F	-2.149091000	4.707274000	-0.025809000
F	-0.588367000	3.842755000	-2.065221000
F	0.659660000	1.476202000	-1.883572000
C	-3.070483000	-2.059925000	-0.044673000
H	-4.672746000	-3.141241000	0.889460000
H	-3.402202000	-3.551165000	2.963557000
H	-1.081035000	-2.663486000	3.196835000
N	-1.824109000	-1.598186000	0.114673000
O	0.089564000	-1.305834000	1.266454000
H	0.123837000	-0.889929000	-1.081635000
H	-0.672611000	-1.129000000	-0.820163000
C	-3.732759000	-1.733442000	-1.370303000
C	-5.165450000	-2.258542000	-1.432126000
H	-5.796087000	-1.819876000	-0.657168000
H	-5.609520000	-2.000478000	-2.393812000
H	-5.210870000	-3.344775000	-1.337327000
C	-3.752547000	-0.209070000	-1.545522000
H	-4.325777000	0.274106000	-0.752768000
H	-2.748263000	0.214941000	-1.535659000
H	-4.214257000	0.053634000	-2.498581000
C	-2.920862000	-2.373369000	-2.503966000
H	-1.899514000	-1.994455000	-2.543458000
H	-2.871683000	-3.457813000	-2.395971000
H	-3.385740000	-2.155178000	-3.466598000

	Hatree	kcal mol-1
E PBEh-3c	-1957.882548	-1228589.899
G	0.2704255	169.6945703
E SMD PBEh-3c	-1957.894124	-1228597.163
E revDSD-PBEP86-D4/def2-QZVPP	-1959.82542	-1229809.069

4

C	-2.739091000	-2.811881000	2.457404000
C	-1.487289000	-2.278516000	2.375026000
C	-3.655637000	-2.708819000	1.393862000
C	-1.107667000	-1.608068000	1.193204000
B	0.492171000	-0.298360000	-0.189974000
C	2.096500000	-0.440684000	-0.252190000
C	2.992238000	0.603133000	-0.408296000
C	2.660605000	-1.707201000	-0.205378000
C	4.363070000	0.415497000	-0.487317000
C	4.021362000	-1.938198000	-0.275873000
C	4.880144000	-0.862919000	-0.413733000
C	-0.112778000	1.194502000	-0.008462000
C	-0.911470000	1.807162000	-0.956121000
C	0.115012000	1.928366000	1.146136000
C	-1.461267000	3.067470000	-0.787894000
C	-0.408519000	3.189909000	1.355733000
C	-1.204286000	3.762148000	0.377638000
F	1.869979000	-2.781196000	-0.108458000
F	2.561343000	1.862305000	-0.507117000
F	5.183479000	1.451464000	-0.631707000
F	6.190496000	-1.060475000	-0.483949000
F	4.510472000	-3.173882000	-0.220275000
F	0.883907000	1.417955000	2.107387000
F	-0.160607000	3.858752000	2.476531000
F	-1.720539000	4.969899000	0.562027000
F	-2.236021000	3.605230000	-1.725728000
F	-1.211334000	1.174227000	-2.100342000
C	-3.284137000	-2.061483000	0.251779000
H	-4.639684000	-3.138921000	1.484335000
H	-3.037911000	-3.327788000	3.360325000
H	-0.769615000	-2.348145000	3.178784000
N	-2.029352000	-1.538603000	0.205660000
O	0.041449000	-1.092925000	1.057348000
H	0.032015000	-0.828635000	-1.189539000
H	-1.710481000	-1.096544000	-0.652040000
C	-4.147925000	-1.847527000	-0.974756000
C	-5.479519000	-2.582166000	-0.831614000
H	-6.065965000	-2.210391000	0.009585000
H	-6.074990000	-2.426051000	-1.730749000
H	-5.344846000	-3.658104000	-0.712353000
C	-3.424365000	-2.379891000	-2.220918000
H	-2.503383000	-1.840834000	-2.451294000
H	-3.178534000	-3.437542000	-2.120635000
H	-4.071055000	-2.268891000	-3.091496000
C	-4.426377000	-0.344623000	-1.133826000

H	-4.908632000	0.066504000	-0.246451000
H	-3.524707000	0.235502000	-1.328484000
H	-5.093983000	-0.184085000	-1.980969000

	Hatree	kcal mol^{-1}
E PBEh-3c	-1957.921182	-1228614.142
G	0.27842831	174.7164096
E SMD PBEh-3c	-1957.936266	-1228623.607
E revDSD-PBEP86-D4/def2-QZVPP	-1959.860733	-1229831.229

5

B	0.686072000	0.009062000	-0.668148000
C	2.188736000	-0.309035000	-0.451348000
C	3.190417000	0.657415000	-0.489098000
C	2.606134000	-1.628402000	-0.293666000
C	4.529371000	0.342878000	-0.370219000
C	3.935420000	-1.976532000	-0.151413000
C	4.897394000	-0.981417000	-0.194554000
C	-0.003746000	1.333871000	-0.247410000
C	-1.081838000	1.819555000	-0.982293000
C	0.343263000	2.052282000	0.893205000
C	-1.763877000	2.968859000	-0.632202000
C	-0.329749000	3.192410000	1.284414000
C	-1.383314000	3.652146000	0.510359000
F	1.706506000	-2.601245000	-0.247124000
F	2.876458000	1.931707000	-0.686584000
F	5.461129000	1.283212000	-0.426374000
F	6.172769000	-1.295787000	-0.069621000
F	4.298938000	-3.239111000	0.019218000
F	1.329092000	1.623662000	1.672015000
F	0.013406000	3.847156000	2.384150000
F	-2.028546000	4.747064000	0.865837000
F	-2.770424000	3.418909000	-1.366882000
F	-1.465419000	1.185477000	-2.082129000
H	0.017781000	-0.817670000	-1.197033000

	Hatree	kcal mol^{-1}
E PBEh-3c	-1478.122986	-927536.2158
G	0.07221061	45.31284378
E SMD PBEh-3c	-1478.125963	-927538.0841
E revDSD-PBEP86-D4/def2-QZVPP	-1479.656391	-928498.4418

6

C	-2.705975000	-2.875273000	2.429203000
C	-1.485239000	-2.291739000	2.382829000
C	-3.632752000	-2.767794000	1.358996000
C	-1.074699000	-1.527800000	1.227868000
C	-3.281083000	-2.056731000	0.255290000
H	-4.596556000	-3.244817000	1.427756000
H	-2.990688000	-3.442899000	3.306592000
H	-0.781691000	-2.374981000	3.198558000
N	-2.049880000	-1.479409000	0.231039000

O	-0.011983000	-0.960672000	1.069861000
H	-1.772150000	-0.947261000	-0.580685000
C	-4.149794000	-1.846557000	-0.971300000
C	-5.503454000	-2.536072000	-0.812344000
H	-6.063099000	-2.143116000	0.037202000
H	-6.105630000	-2.367828000	-1.705692000
H	-5.400073000	-3.614453000	-0.686954000
C	-3.447328000	-2.434648000	-2.203875000
H	-2.495846000	-1.948110000	-2.424615000
H	-3.251015000	-3.499497000	-2.075090000
H	-4.078168000	-2.313638000	-3.085695000
C	-4.392803000	-0.344145000	-1.177029000
H	-4.880688000	0.098336000	-0.307968000
H	-3.474042000	0.215249000	-1.360640000
H	-5.039061000	-0.185938000	-2.041583000

	Hatree	kcal mol^{-1}
E PBEh-3c	-479.7423749	-301042.8978
G	0.17810151	111.7603895
E SMD PBEh-3c	-479.7559576	-301051.4211
E revDSD-PBEP86-D4/def2-QZVPP	-480.1510918	-301299.3715

8

C	1.625630000	0.214672000	1.467576000
C	2.209614000	0.060962000	2.740485000
C	3.568899000	0.000336000	2.840361000
C	4.392350000	0.084128000	1.704534000
C	3.818155000	0.231757000	0.473838000
N	2.464525000	0.306550000	0.412746000
H	4.025029000	-0.121571000	3.813985000
H	1.558312000	-0.010930000	3.598779000
H	5.463490000	0.024041000	1.806798000
H	2.006396000	0.324840000	-0.498864000
N	0.814803000	-2.230857000	-0.295683000
C	0.222772000	-1.511527000	-1.224085000
C	0.849746000	-3.559762000	-0.399860000
C	-0.379959000	-2.068187000	-2.350839000
C	0.276232000	-4.206052000	-1.485250000
C	-0.343947000	-3.441332000	-2.467462000
H	-0.857685000	-1.436948000	-3.086641000
H	0.301386000	-5.281556000	-1.575543000
H	-0.801984000	-3.924787000	-3.320736000
O	0.246473000	-0.174785000	-1.086819000
O	0.364920000	0.271987000	1.329995000
B	-0.428380000	0.466372000	0.041497000
C	1.547325000	-4.280084000	0.744017000
C	1.593105000	-5.792191000	0.536065000
H	2.109052000	-6.264812000	1.372969000
H	0.594789000	-6.230174000	0.486302000
H	2.131291000	-6.067216000	-0.373086000
C	2.982093000	-3.751902000	0.857781000
H	2.991548000	-2.678110000	1.040596000

H	3.502074000	-4.239167000	1.684843000
H	3.551451000	-3.947989000	-0.053606000
C	0.790509000	-3.975740000	2.042210000
H	0.743812000	-2.904739000	2.231536000
H	-0.233715000	-4.349206000	2.000494000
H	1.285358000	-4.453361000	2.890280000
C	4.555741000	0.327898000	-0.846491000
C	4.026143000	-0.740987000	-1.815020000
H	2.987681000	-0.580540000	-2.107702000
H	4.613747000	-0.721026000	-2.733256000
H	4.100701000	-1.741799000	-1.389114000
C	6.053647000	0.106715000	-0.646031000
H	6.270522000	-0.876300000	-0.225530000
H	6.558714000	0.167137000	-1.609857000
H	6.500551000	0.865695000	-0.002451000
C	4.334892000	1.726869000	-1.441722000
H	4.680818000	2.507315000	-0.763085000
H	4.895237000	1.821972000	-2.372394000
H	3.289641000	1.924104000	-1.678482000
C	-0.487222000	2.082485000	-0.188081000
C	-1.144323000	2.859510000	0.755982000
C	0.065752000	2.782446000	-1.248055000
C	-1.267228000	4.233564000	0.662632000
C	-0.028296000	4.159119000	-1.375780000
C	-0.702495000	4.888140000	-0.416515000
C	-1.901139000	-0.187958000	0.252530000
C	-2.831080000	-0.007147000	-0.760521000
C	-2.325121000	-0.964562000	1.318880000
C	-4.105420000	-0.541125000	-0.734250000
C	-3.594218000	-1.518480000	1.382354000
C	-4.488642000	-1.304599000	0.352461000
F	0.749078000	2.156989000	-2.212485000
F	0.528841000	4.782979000	-2.409866000
F	-0.800529000	6.206258000	-0.524436000
F	-1.912169000	4.927880000	1.593797000
F	-1.697678000	2.277669000	1.820555000
F	-2.499596000	0.710159000	-1.838210000
F	-1.525460000	-1.228367000	2.351187000
F	-3.957731000	-2.258918000	2.425906000
F	-5.705238000	-1.831281000	0.403047000
F	-4.956140000	-0.335717000	-1.735004000

	Hatree	kcal mol^{-1}
E PBEh-3c	-2436.529561	-1528945.447
G	0.46069616	289.091217
E SMD PBEh-3c	-2436.549981	-1528958.261
E revDSD-PBEP86-D4/def2-QZVPP	-2438.868041	-1530412.865

TS$_{6/11}$

C	2.748485000	-3.120795000	1.481826000
C	1.403562000	-2.942644000	1.222439000
C	0.931062000	-1.679341000	0.902256000

C	1.755661000	-0.567349000	0.820418000
C	3.101013000	-0.795796000	1.086775000
B	1.283127000	0.883531000	0.345449000
C	-0.199429000	1.116356000	-0.206976000
C	-0.392723000	1.260816000	-1.573458000
C	-1.343279000	1.208541000	0.569515000
C	-1.633960000	1.494966000	-2.141383000
C	-2.599059000	1.448078000	0.045137000
C	-2.743361000	1.591736000	-1.323223000
F	-0.375300000	-1.576334000	0.668669000
F	3.213644000	-4.319799000	1.796303000
F	3.958506000	0.223265000	1.048485000
F	4.900305000	-2.202622000	1.672810000
F	0.576113000	-3.978329000	1.285646000
F	-1.263115000	1.056898000	1.893992000
F	-3.662985000	1.532977000	0.835313000
F	-3.939619000	1.817430000	-1.845413000
F	-1.767316000	1.627142000	-3.455536000
F	0.643297000	1.168335000	-2.406272000
C	1.442468000	1.675299000	2.306972000
C	1.581762000	2.535459000	1.460838000
H	2.117326000	1.357274000	-0.369021000
C	3.608107000	-2.037417000	1.417625000
C	1.755165000	3.729695000	0.646893000
H	1.740058000	4.612612000	1.284484000
H	2.703238000	3.693183000	0.112787000
H	0.953914000	3.816363000	-0.085933000
C	1.338571000	0.814634000	3.473949000
H	0.550254000	0.074086000	3.365075000
H	2.278613000	0.297012000	3.659599000
H	1.108173000	1.430673000	4.343345000

	Hatree	kcal mol^{-1}
E PBEh-3c	-1633.762793	-1025201.673
G	0.15663019	98.28693221
E SMD PBEh-3c	-1633.770697	-1025206.633
E revDSD-PBEP86-D4/def2-QZVPP	-1635.420559	-1026241.937

11

C	2.645866000	-3.311944000	0.647273000
C	1.384002000	-3.148394000	1.193704000
C	0.860232000	-1.873556000	1.295307000
C	1.556175000	-0.746921000	0.889549000
C	2.816415000	-0.950186000	0.350180000
B	0.913124000	0.684590000	0.995095000
C	-0.479358000	0.894685000	0.296278000
C	-0.699866000	0.499577000	-1.013903000
C	-1.554828000	1.456756000	0.966540000
C	-1.918993000	0.662686000	-1.644812000
C	-2.793242000	1.622016000	0.375400000
C	-2.969508000	1.224049000	-0.939149000
F	-0.350372000	-1.730089000	1.831630000

F	3.163302000	-4.524198000	0.535791000
F	3.519963000	0.088447000	-0.091150000
F	4.572025000	-2.378344000	-0.312788000
F	0.700680000	-4.205882000	1.609703000
F	-1.414445000	1.828073000	2.236136000
F	-3.807171000	2.150474000	1.046965000
F	-4.145420000	1.382464000	-1.523088000
F	-2.092332000	0.292461000	-2.906039000
F	0.300562000	-0.029941000	-1.714364000
C	1.605974000	1.822887000	1.753717000
C	2.482408000	1.472184000	2.716937000
H	2.710225000	0.419110000	2.852345000
C	3.174779000	2.382286000	3.667603000
H	2.970341000	2.083453000	4.697890000
H	4.257471000	2.307041000	3.538273000
H	2.890174000	3.425371000	3.553451000
C	1.250617000	3.268460000	1.509143000
H	0.647687000	3.390346000	0.610673000
H	0.686657000	3.701091000	2.337120000
H	2.144467000	3.878304000	1.371965000
C	3.368322000	-2.211427000	0.218429000

	Hatree	kcal mol^{-1}
E PBEh-3c	-1633.83123	-1025244.618
G	0.15896408	99.75147036
E SMD PBEh-3c	-1633.837673	-1025248.661
E revDSD-PBEP86-D4/def2-QZVPP	-1635.477396	-1026277.603

12

C	1.505918000	0.103403000	1.319820000
C	1.963597000	0.099868000	2.652145000
C	3.287705000	0.317750000	2.898504000
C	4.199363000	0.540983000	1.853477000
C	3.754109000	0.543134000	0.561514000
N	2.426485000	0.335374000	0.352798000
H	3.646049000	0.317524000	3.919462000
H	1.245389000	-0.075087000	3.439241000
H	5.242082000	0.702455000	2.072401000
H	2.090825000	0.238767000	-0.605347000
O	0.288302000	-0.116817000	1.043148000
B	-0.466261000	0.251284000	-0.255789000
C	4.620197000	0.790743000	-0.659700000
C	4.258452000	-0.182200000	-1.791278000
H	3.258669000	-0.025690000	-2.199955000
H	4.951300000	-0.040963000	-2.620761000
H	4.332301000	-1.222251000	-1.470419000
C	6.097907000	0.601920000	-0.313855000
H	6.304368000	-0.395890000	0.075397000
H	6.697394000	0.735821000	-1.214087000
H	6.451881000	1.336052000	0.410585000
C	4.403683000	2.237500000	-1.128495000
H	4.640260000	2.951052000	-0.338665000

H	5.055781000	2.449610000	-1.976792000
H	3.382401000	2.420128000	-1.455749000
C	-0.237563000	1.876963000	-0.360861000
C	-0.730685000	2.698237000	0.646627000
C	0.482749000	2.535625000	-1.340455000
C	-0.557024000	4.069117000	0.671478000
C	0.686012000	3.906344000	-1.356564000
C	0.157580000	4.679937000	-0.344012000
C	-2.021172000	-0.162522000	0.013810000
C	-3.107712000	0.575302000	-0.427087000
C	-2.338094000	-1.367669000	0.625338000
C	-4.422217000	0.176375000	-0.254907000
C	-3.637669000	-1.799590000	0.823979000
C	-4.689077000	-1.018759000	0.383047000
F	1.073252000	1.854309000	-2.335148000
F	1.405157000	4.472921000	-2.322140000
F	0.344451000	5.992617000	-0.335235000
F	-1.058461000	4.804765000	1.657131000
F	-1.410715000	2.165203000	1.662018000
F	-2.926166000	1.734910000	-1.070304000
F	-1.372969000	-2.188441000	1.043093000
F	-3.884409000	-2.959732000	1.426645000
F	-5.942006000	-1.419101000	0.561522000
F	-5.425117000	0.930208000	-0.695669000
C	0.004776000	-0.623105000	-1.527358000
C	0.835328000	-1.671704000	-1.445646000
H	1.260464000	-1.941892000	-0.481907000
C	-0.725705000	-0.335333000	-2.819638000
H	-0.065985000	-0.322805000	-3.687916000
H	-1.480254000	-1.103450000	-3.002698000
H	-1.251350000	0.617562000	-2.804950000
C	1.191205000	-2.624889000	-2.544336000
H	2.263450000	-2.833931000	-2.557570000
H	0.691263000	-3.586178000	-2.399739000
H	0.913454000	-2.261296000	-3.531895000

	Hatree	kcal mol^{-1}
E PBEh-3c	-2113.61519	-1326313.611
G	0.36549595	229.3521808
E SMD PBEh-3c	-1633.770697	-1025206.633
E revDSD-PBEP86-D4/def2-QZVPP	-1635.420559	-1026241.937

TS$_{12/3+\textit{cis}\text{-butene}}$

C	1.803149000	-0.237490000	1.296272000
C	2.417799000	-0.714497000	2.459812000
C	3.789165000	-0.709058000	2.491332000
C	4.527965000	-0.251809000	1.396006000
C	3.855379000	0.182189000	0.272692000
N	2.515757000	0.175180000	0.259703000
H	4.307218000	-1.056491000	3.375801000
H	1.817236000	-1.052392000	3.291838000
H	5.605792000	-0.238117000	1.441699000

H	1.472799000	0.114709000	-0.837447000
O	0.496437000	-0.206358000	1.203792000
B	-0.219891000	0.370667000	0.061303000
C	4.529019000	0.685358000	-0.991598000
C	4.257193000	-0.312926000	-2.125183000
H	3.191149000	-0.424120000	-2.326453000
H	4.732799000	0.026316000	-3.047288000
H	4.657648000	-1.299864000	-1.888332000
C	6.039965000	0.821970000	-0.813042000
H	6.517820000	-0.134864000	-0.597037000
H	6.486041000	1.202022000	-1.732808000
H	6.294523000	1.520351000	-0.014396000
C	3.949993000	2.057152000	-1.359405000
H	4.113346000	2.783463000	-0.562088000
H	4.430771000	2.437628000	-2.262217000
H	2.877232000	2.011854000	-1.546905000
C	-0.174505000	2.003266000	-0.020522000
C	-1.008359000	2.667610000	-0.913494000
C	0.559921000	2.840770000	0.809856000
C	-1.095374000	4.043195000	-1.019213000
C	0.503815000	4.223765000	0.737083000
C	-0.326194000	4.830496000	-0.183924000
C	-1.767698000	-0.117910000	0.234412000
C	-2.583327000	-0.742235000	-0.695138000
C	-2.373372000	0.154421000	1.456101000
C	-3.892219000	-1.111189000	-0.430646000
C	-3.677430000	-0.193740000	1.758276000
C	-4.442190000	-0.839754000	0.805759000
F	1.364674000	2.354141000	1.753242000
F	1.238635000	4.971356000	1.553081000
F	-0.387804000	6.151639000	-0.263847000
F	-1.904169000	4.608933000	-1.908327000
F	-1.784697000	1.964797000	-1.746836000
F	-2.149543000	-1.020879000	-1.928826000
F	-1.700926000	0.810293000	2.401301000
F	-4.201093000	0.090775000	2.945696000
F	-5.693912000	-1.185849000	1.072997000
F	-4.623218000	-1.718595000	-1.360689000
C	0.488406000	-0.369232000	-1.387129000
C	0.419455000	0.312181000	-2.557627000
H	0.538997000	1.390204000	-2.503845000
C	0.536746000	-1.880848000	-1.330431000
H	0.150102000	-2.363296000	-2.225232000
H	1.569606000	-2.214241000	-1.205308000
H	-0.021627000	-2.276787000	-0.483489000
C	0.239033000	-0.206704000	-3.929899000
H	-0.677026000	0.219554000	-4.347486000
H	1.052313000	0.138208000	-4.572117000
H	0.176368000	-1.288704000	-3.991468000

H_2

H	0.000000000	0.000000000	0.371229000

H	0.000000000	0.000000000	-0.371229000

	Hatree	kcal mol^{-1}
E PBEh-3c	-1.168032918	-732.9517524
G	-0.0012677	-0.795493793
E SMD PBEh-3c	-1.167593129	-732.6757808
E revDSD-PBEP86-D4/def2-QZVPP	-1.171742422	-735.2795012

2-butyne

C	-1.092318000	-0.000006000	-0.481513000
C	-1.092272000	0.000008000	0.719896000
C	-1.092375000	-0.000011000	-1.935393000
H	-0.591738000	-0.884533000	-2.330384000
H	-2.108730000	0.008620000	-2.330318000
H	-0.576737000	0.875823000	-2.330439000
C	-1.092327000	0.000002000	2.173776000
H	-2.108704000	-0.008653000	2.568654000
H	-0.576730000	-0.875858000	2.568814000
H	-0.591724000	0.884515000	2.568821000

	Hatree	kcal mol^{-1}
E PBEh-3c	-155.6246044	-97655.91772
G	0.0585281	36.72693877
E SMD PBEh-3c	-155.6312037	-97660.05884
E revDSD-PBEP86-D4/def2-QZVPP	-155.7503581	-97734.82936

***cis*-butene**

C	-1.988973000	0.540100000	-1.115632000
H	-1.858778000	1.512920000	-0.651826000
C	-1.990036000	-0.520944000	-0.313159000
H	-1.860666000	-0.339574000	0.749293000
C	-2.146814000	0.563476000	-2.600785000
H	-1.275293000	1.018173000	-3.076481000
H	-2.278757000	-0.425998000	-3.034251000
H	-3.009361000	1.167591000	-2.889804000
C	-2.149506000	-1.956053000	-0.695361000
H	-1.280682000	-2.539475000	-0.383451000
H	-3.015249000	-2.396570000	-0.196421000
H	-2.277128000	-2.103331000	-1.766059000

	Hatree	kcal mol^{-1}
E PBEh-3c	-156.8716807	-98438.46992
G	0.08369802	52.52130268
E SMD PBEh-3c	-156.8769041	-98441.74765
E revDSD-PBEP86-D4/def2-QZVPP	-156.9913723	-98513.57752

13

C	3.805708000	-3.611228000	-0.197935000
C	2.578810000	-3.223910000	-0.651985000
C	4.343436000	-3.096824000	0.995034000
C	1.845471000	-2.280486000	0.092416000
B	-0.050005000	-0.647254000	0.262541000

C	-1.470760000	-0.690884000	-0.517851000
C	-1.810105000	0.139478000	-1.573621000
C	-2.432435000	-1.626288000	-0.162617000
C	-3.034402000	0.079511000	-2.219289000
C	-3.664988000	-1.719663000	-0.782660000
C	-3.969180000	-0.853445000	-1.816159000
C	0.788868000	0.747343000	0.053398000
C	0.287081000	1.898602000	0.647037000
C	1.970823000	0.918607000	-0.645580000
C	0.915306000	3.127710000	0.584098000
C	2.640201000	2.129970000	-0.724902000
C	2.109195000	3.242330000	-0.104974000
F	-2.182899000	-2.508182000	0.804772000
F	-0.945442000	1.043946000	-2.036984000
F	-3.313866000	0.902965000	-3.225116000
F	-5.145737000	-0.927578000	-2.424624000
F	-4.553781000	-2.632950000	-0.401831000
F	2.544212000	-0.102647000	-1.298084000
F	3.784876000	2.228002000	-1.395873000
F	2.734281000	4.410712000	-0.172640000
F	0.387447000	4.195841000	1.172426000
F	-0.873820000	1.850033000	1.299101000
C	3.608097000	-2.211220000	1.729114000
H	5.323823000	-3.401448000	1.323569000
H	4.385426000	-4.320591000	-0.773650000
H	2.160014000	-3.589221000	-1.577637000
N	2.387579000	-1.856328000	1.252419000
O	0.723142000	-1.838941000	-0.315209000
H	1.783697000	-1.263502000	1.829560000
C	4.011510000	-1.592449000	3.051677000
C	5.472302000	-1.901257000	3.373112000
H	5.743251000	-1.425258000	4.315314000
H	5.651901000	-2.970545000	3.492288000
H	6.148873000	-1.519990000	2.607124000
C	3.121759000	-2.181067000	4.157136000
H	2.063292000	-1.975848000	3.991655000
H	3.244684000	-3.262074000	4.231318000
H	3.390505000	-1.745522000	5.120554000
C	3.830775000	-0.067605000	2.990005000
H	4.403556000	0.371453000	2.172285000
H	2.789555000	0.239208000	2.871908000
H	4.183005000	0.378211000	3.920593000
C	-0.227696000	-0.830526000	1.826803000
C	-0.343982000	-0.891311000	3.030132000
C	-0.588660000	-0.926141000	4.467182000
H	-0.173116000	-1.860145000	4.871410000
C	-2.097999000	-0.929453000	4.751947000
H	-2.539535000	-0.034709000	4.303008000
H	-2.560851000	-1.785408000	4.257962000
C	0.094255000	0.246747000	5.183968000
H	1.171895000	0.217182000	5.001698000
H	-0.267599000	1.181698000	4.745689000

C	-2.374655000	-0.951129000	6.250367000
H	-3.452023000	-0.923210000	6.426999000
H	-2.018840000	-1.897302000	6.672992000
C	-0.191944000	0.224169000	6.680871000
H	0.268381000	-0.665351000	7.125747000
H	0.277488000	1.085643000	7.161094000
C	-1.690462000	0.210963000	6.962043000
H	-2.129929000	1.153707000	6.619891000
H	-1.872792000	0.157744000	8.037685000

	Hatree	kcal mol^{-1}
E PBEh-3c	-2268.097061	-1423252.452
G	0.43594326	273.5585371
E SMD PBEh-3c	-2268.11828	-1423265.768
E revDSD-PBEP86-D4/def2-QZVPP	-2270.272684	-1424617.677

TS$_{13/3}$

C	3.092247000	-2.943100000	-2.269895000
C	1.775292000	-2.558104000	-2.292812000
C	3.918831000	-2.586802000	-1.201910000
C	1.311344000	-1.797522000	-1.214488000
B	-0.536447000	-0.452493000	-0.282794000
C	-2.135884000	-0.626722000	-0.399012000
C	-2.974721000	0.318686000	-0.966294000
C	-2.749747000	-1.800329000	0.012440000
C	-4.344778000	0.142124000	-1.075294000
C	-4.113175000	-2.011681000	-0.070675000
C	-4.917358000	-1.028300000	-0.617120000
C	0.042619000	1.061686000	-0.330969000
C	-0.416486000	2.003852000	0.579565000
C	1.015635000	1.517501000	-1.206616000
C	0.069544000	3.292740000	0.662602000
C	1.533100000	2.802626000	-1.156892000
C	1.061452000	3.693749000	-0.214032000
F	-2.021081000	-2.790522000	0.530035000
F	-2.483928000	1.456461000	-1.456866000
F	-5.109451000	1.082182000	-1.619616000
F	-6.227217000	-1.212512000	-0.707336000
F	-4.655095000	-3.143713000	0.369033000
F	1.499756000	0.732209000	-2.168657000
F	2.474589000	3.185330000	-2.012696000
F	1.545272000	4.926413000	-0.155580000
F	-0.405680000	4.145696000	1.564440000
F	-1.397851000	1.679156000	1.428387000
C	3.383043000	-1.856930000	-0.160365000
H	4.957761000	-2.877969000	-1.203659000
H	3.497549000	-3.521722000	-3.089824000
H	1.110796000	-2.808028000	-3.107165000
N	2.095606000	-1.489425000	-0.196430000
O	0.056180000	-1.393959000	-1.205124000
H	1.070923000	-1.144284000	0.955205000
C	4.161482000	-1.414106000	1.066738000

C	5.649931000	-1.733836000	0.939145000
H	6.179267000	-1.378966000	1.824098000
H	5.836086000	-2.806365000	0.862562000
H	6.099878000	-1.245559000	0.073256000
C	3.606222000	-2.145055000	2.296582000
H	2.555152000	-1.914574000	2.470100000
H	3.698497000	-3.227071000	2.191113000
H	4.158628000	-1.848193000	3.189995000
C	3.993887000	0.099837000	1.247966000
H	4.374338000	0.646543000	0.383800000
H	2.950241000	0.381846000	1.385911000
H	4.548884000	0.435783000	2.125581000
C	-0.001209000	-0.909211000	1.353850000
C	-0.521102000	-0.903971000	2.465907000
C	-1.191945000	-0.880929000	3.743517000
H	-0.911288000	-1.802381000	4.272451000
C	-0.793096000	0.330943000	4.597196000
H	0.284494000	0.318897000	4.772338000
H	-1.010196000	1.243645000	4.037329000
C	-2.719882000	-0.934192000	3.516385000
H	-3.019856000	-0.079102000	2.905566000
H	-2.977578000	-1.835141000	2.958883000
C	-1.550403000	0.335991000	5.919271000
H	-1.246915000	-0.528552000	6.519779000
H	-1.272805000	1.221202000	6.494694000
C	-3.456028000	-0.906740000	4.850938000
H	-4.531651000	-0.903286000	4.663803000
H	-3.242499000	-1.828851000	5.401798000
C	-3.057507000	0.296826000	5.696141000
H	-3.581420000	0.273244000	6.653810000
H	-3.373829000	1.215924000	5.192285000

	Hatree	kcal mol^{-1}
E PBEh-3c	-2268.05852	-1423228.268
G	0.43256407	271.4380633
E SMD PBEh-3c	-2268.077204	-1423239.992
E revDSD-PBEP86-D4/def2-QZVPP	-2270.233488	-1424593.081

TS$_{3/14}$

C	2.956138000	-2.912940000	-2.334786000
C	1.674365000	-2.425522000	-2.321735000
C	3.815039000	-2.688506000	-1.255670000
C	1.273075000	-1.695480000	-1.196257000
B	-0.471315000	-0.268222000	-0.168690000
C	-2.081621000	-0.406324000	-0.218909000
C	-2.914650000	0.534688000	-0.801044000
C	-2.713240000	-1.538639000	0.272839000
C	-4.291832000	0.392877000	-0.854897000
C	-4.083659000	-1.716865000	0.243303000
C	-4.879391000	-0.738126000	-0.322863000
C	0.124886000	1.240888000	-0.269415000
C	-0.280001000	2.199165000	0.650127000

C	1.059889000	1.673482000	-1.196089000
C	0.211688000	3.488480000	0.681425000
C	1.579915000	2.958800000	-1.199871000
C	1.155905000	3.870615000	-0.254270000
F	-1.992790000	-2.531822000	0.803299000
F	-2.409800000	1.633708000	-1.361671000
F	-5.049725000	1.327820000	-1.417567000
F	-6.196218000	-0.890442000	-0.363136000
F	-4.640082000	-2.815869000	0.745364000
F	1.506001000	0.862569000	-2.156102000
F	2.479634000	3.321234000	-2.108131000
F	1.643746000	5.103558000	-0.245260000
F	-0.210886000	4.359443000	1.593093000
F	-1.206114000	1.888573000	1.563672000
C	3.346529000	-1.979852000	-0.169000000
H	4.825977000	-3.064242000	-1.284197000
H	3.309511000	-3.472487000	-3.191197000
H	0.987473000	-2.575393000	-3.142060000
N	2.092417000	-1.507395000	-0.173797000
O	0.053099000	-1.209188000	-1.148441000
H	1.169583000	-1.075676000	0.972813000
C	4.162804000	-1.678009000	1.075982000
C	5.612419000	-2.135635000	0.926017000
H	6.172341000	-1.885228000	1.827718000
H	5.691530000	-3.215370000	0.788943000
H	6.110701000	-1.646832000	0.087325000
C	3.537503000	-2.413230000	2.269457000
H	2.513382000	-2.092850000	2.461223000
H	3.525261000	-3.492244000	2.108307000
H	4.115734000	-2.217757000	3.174286000
C	4.146372000	-0.165905000	1.333832000
H	4.576154000	0.383909000	0.495296000
H	3.137166000	0.211611000	1.495688000
H	4.734208000	0.068899000	2.222857000
C	0.113983000	-0.724229000	1.391943000
C	-0.334535000	-0.659334000	2.538157000
C	-0.903323000	-0.610553000	3.819628000
C	-0.740959000	0.523146000	4.627686000
C	-1.656939000	-1.698590000	4.282454000
C	-1.313249000	0.556937000	5.883907000
C	-2.230564000	-1.651378000	5.538022000
C	-2.055750000	-0.527604000	6.336687000
H	-0.170151000	1.362809000	4.256500000
H	-1.787984000	-2.560131000	3.642218000
H	-1.190070000	1.429227000	6.510799000
H	-2.816630000	-2.486321000	5.896382000
H	-2.506175000	-0.494406000	7.319953000

	Hatree	kcal mol^{-1}
E PBEh-3c	-2264.4278	-1420949.957
G	0.36026993	226.0728036
E SMD PBEh-3c	-2264.448482	-1420962.934

E revDSD-PBEP86-D4/def2-QZVPP -2266.610437 -1422319.582

14

C	3.655420000	-3.726915000	-0.161604000
C	2.438655000	-3.323673000	-0.629599000
C	4.201028000	-3.196292000	1.020437000
C	1.720532000	-2.351806000	0.092078000
B	-0.140674000	-0.685076000	0.240973000
C	-1.512731000	-0.622593000	-0.617106000
C	-1.840519000	0.366404000	-1.529270000
C	-2.452872000	-1.632442000	-0.464960000
C	-3.033023000	0.374571000	-2.235246000
C	-3.650843000	-1.663171000	-1.153610000
C	-3.943353000	-0.645422000	-2.043344000
C	0.793071000	0.661221000	0.145138000
C	0.433767000	1.783546000	0.880540000
C	1.942563000	0.803738000	-0.614306000
C	1.173856000	2.951221000	0.902725000
C	2.718339000	1.951906000	-0.614644000
C	2.332502000	3.032692000	0.152106000
F	-2.209365000	-2.644358000	0.367170000
F	-1.000707000	1.370989000	-1.784644000
F	-3.303824000	1.348609000	-3.098543000
F	-5.087418000	-0.655160000	-2.714380000
F	-4.518565000	-2.654526000	-0.974829000
F	2.372579000	-0.187344000	-1.405397000
F	3.825501000	2.020997000	-1.348129000
F	3.060989000	4.140932000	0.163223000
F	0.784265000	3.992220000	1.630433000
F	-0.691857000	1.776223000	1.592801000
C	3.482995000	-2.279269000	1.732908000
H	5.175391000	-3.511137000	1.357080000
H	4.222022000	-4.460624000	-0.719583000
H	2.016009000	-3.699810000	-1.549128000
N	2.267795000	-1.916261000	1.246666000
O	0.611255000	-1.893128000	-0.331839000
H	1.682666000	-1.301516000	1.813744000
C	3.903657000	-1.621633000	3.031276000
C	5.350021000	-1.974657000	3.371909000
H	5.636063000	-1.471823000	4.295430000
H	5.485027000	-3.044724000	3.535581000
H	6.045167000	-1.652306000	2.595390000
C	2.989407000	-2.119421000	4.160898000
H	1.942023000	-1.859940000	4.005159000
H	3.052012000	-3.202231000	4.274339000
H	3.290091000	-1.665877000	5.105888000
C	3.786774000	-0.094786000	2.897443000
H	4.392406000	0.284026000	2.073194000
H	2.761259000	0.247375000	2.743917000
H	4.137440000	0.377981000	3.814974000
C	-0.422516000	-0.961256000	1.775086000
C	-0.580173000	-1.133447000	2.963503000

C	-0.774221000	-1.304664000	4.363790000
C	-0.951956000	-2.577291000	4.911730000
C	-0.773905000	-0.192749000	5.210266000
C	-1.119700000	-2.731711000	6.277364000
C	-0.944659000	-0.354233000	6.574320000
C	-1.115395000	-1.622689000	7.111945000
H	-0.963836000	-3.437970000	4.256390000
H	-0.646996000	0.793416000	4.783505000
H	-1.260244000	-3.721218000	6.691846000
H	-0.948152000	0.513564000	7.220496000
H	-1.250093000	-1.746080000	8.178320000

	Hatree	kcal mol^{-1}
E PBEh-3c	-2264.465303	-1420973.49
G	0.36689016	230.2270609
E SMD PBEh-3c	-2264.487861	-1420987.646
E revDSD-PBEP86-D4/def2-QZVPP	-2266.65021	-1422344.54

Cyclohexylacetylene

C	-0.180365000	-0.832059000	1.833905000
C	-0.370912000	-0.883655000	3.018623000
C	-0.617758000	-0.917937000	4.454821000
H	-0.190329000	-1.850782000	4.845232000
C	-2.121606000	-0.923416000	4.760373000
H	-2.572766000	-0.027145000	4.323476000
H	-2.593525000	-1.778943000	4.273527000
C	0.076579000	0.252279000	5.165670000
H	1.148810000	0.221481000	4.963097000
H	-0.294170000	1.190744000	4.742084000
C	-2.376874000	-0.952133000	6.262922000
H	-3.451639000	-0.926062000	6.455717000
H	-2.014161000	-1.900176000	6.675055000
C	-0.188203000	0.220409000	6.666581000
H	0.279606000	-0.670668000	7.099431000
H	0.289091000	1.079022000	7.144062000
C	-1.682069000	0.205999000	6.970659000
H	-2.127134000	1.150754000	6.640902000
H	-1.848685000	0.146089000	8.048532000
H	-0.005895000	-0.796925000	0.785986000

	Hatree	kcal mol^{-1}
E PBEh-3c	-311.3289959	-195361.9025
G	0.15337512	96.24434486
E SMD PBEh-3c	-311.3380095	-195367.5587
E revDSD-PBEP86-D4/def2-QZVPP	-311.5740831	-195515.6971

Phenylacetylene

C	-0.433973000	-0.976382000	1.777906000
C	-0.581840000	-1.121230000	2.962001000
C	-0.765077000	-1.291329000	4.365410000
C	-0.943680000	-2.566928000	4.903877000
C	-0.768347000	-0.184553000	5.216167000

C	-1.122264000	-2.728782000	6.267023000
C	-0.947461000	-0.353695000	6.578376000
C	-1.124731000	-1.624367000	7.107345000
H	-0.941380000	-3.425881000	4.246663000
H	-0.630100000	0.804659000	4.801345000
H	-1.259985000	-3.721224000	6.675088000
H	-0.948644000	0.510073000	7.229790000
H	-1.264536000	-1.753665000	8.172348000
H	-0.301756000	-0.850733000	0.730563000

	Hatree	kcal mol^{-1}
E PBEh-3c	-307.6955129	-193081.8574
G	0.08290172	52.02161687
E SMD PBEh-3c	-307.7059633	-193088.4152
E revDSD-PBEP86-D4/def2-QZVPP	-307.9494335	-193241.1951

7 References

[1] L. Hintermann, T. T. Dang, A. Labonne, T. Kribber, L. Xiao, P. Naumov, *Chem. Eur. J.* **2009**, *15*, 7167.

[2] Y. Soltani, L. C. Wilkins, R. L. Melen, *Angew. Chem. Int. Ed.* **2017**, *56*, 11995.

[3] L. E. Longobardi, T. C. Johnstone, R. L. Falconer, C. A. Russell, D. W. Stephan, *Chem. Eur. J.* **2016**, *22*, 12665.

[4] A. B. Smith, G. R. Ott, *J. Am. Chem. Soc.* **1998**, *120*, 3935.

[5] H. Ehrhorn, J. Schlösser, D. Bockfeld, M. Tamm, *Beilstein J. Org. Chem.* **2018**, *14*, 2425.

[6] M. Hasenbeck, T. Müller, U. Gellrich, *Catal. Sci. Technol.* **2019**, *9*, 2438.

[7] U. Gellrich, *Angew. Chem. Int. Ed.* **2018**, *57*, 4779.

[8] Y. Liu, L. Hu, H. Chen, H. Du, *Chem. Eur. J.* **2015**, *21*, 3495.

[9] a) S. Grimme, J. Antony, S. Ehrlich, H. Krieg, *J. Chem. Phys.* **2010**, *132*, 154104; b) S. Grimme, J. G. Brandenburg, C. Bannwarth, A. Hansen, *J. Chem. Phys.* **2015**, *143*, 54107; c) S. Grimme, S. Ehrlich, L. Goerigk, *J. Comp. Chem.* **2011**, *32*, 1456; d) H. Kruse, S. Grimme, *J. Chem. Phys.* **2012**, *136*, 154101; e) F. Weigend, *Phys. Chem. Chem. Phys.* **2006**, *8*, 1057.

[10] A. V. Marenich, C. J. Cramer, D. G. Truhlar, *J. Phys. Chem. B* **2009**, *113*, 6378.

[11] a) G. Santra, N. Sylvetsky, J. M. L. Martin, *J. Phys. Chem. A* **2019**, *123*, 5129; b) E. Caldeweyher, C. Bannwarth, S. Grimme, *J. Chem. Chem. Phys.* **2017**, *147*, 34112; c) E. Caldeweyher, S. Ehlert, A. Hansen, H. Neugebauer, S. Spicher, C. Bannwarth, S. Grimme, *J. Chem. Phys.* **2019**, *150*, 154122; d) F. Weigend, R. Ahlrichs, *Phys. Chem. Chem. Phys.* **2005**, *7*, 3297; e) A. Hellweg, C. Hättig, S. Höfener, W. Klopper, *Theor. Chem. Acc.* **2007**, *117*, 587; f) F. Weigend, *J. Chem. Theory Comput.* **2008**, *29*, 167.
